# Supplementary material for: Rickettsial DNA and a trans-splicing rRNA group I intron in the unorthodox mitogenome of the fern Haplopteris ensiformis
Source: Commun Biol. 2023 Mar 20;6:296. doi: 10.1038/s42003-023-04659-8 (PMC10027690; doi:10.1038/s42003-023-04659-8)
Supplement: Supplementary file 2 — Supplementary Information [file 42003_2023_4659_MOESM2_ESM.pdf]

Supplementary Information

Rickettsial DNA invasions and a scrambled rRNA cluster with a *trans*-splicing group I intron: The highly unorthodox mitogenome of the fern *Haplopteris ensiformis*

Simon Zumkeller, Monika Polsakiewicz and Volker Knoop*

Supplementary figure 1. The *accD* gene example for chloroplast RNA editing in *Haplopteris ensiformis*.

a. Sequence alignment of the *H. ensiformis accD* gene below its homologue in *Psilotum nudum* shown as one selected example reference out of 22 used for prediction of RNA editing. Alignment was created by PREPACT [90] with identical nucleotides and amino acid shown in grey font and predicted C-to-U RNA editing in blue and reverse U-to-C editing in red. Codons framed by boxes were confirmed as editing sites. The stippled rectangle highlights potential reverse edit accDeC580FL remaining unconfirmed but strongly suggested by the chloroplast editome references in PREPACT 3.0 with the exception of *Ginkgo biloba*. The remaining cases are weak predictions only that are not supported by the majority of other editome references. **b**. The list of expected and observed *accD* edits including those in the 5' and 3'-UTRs and the respective editing frequencies observed with additional remarks.

a

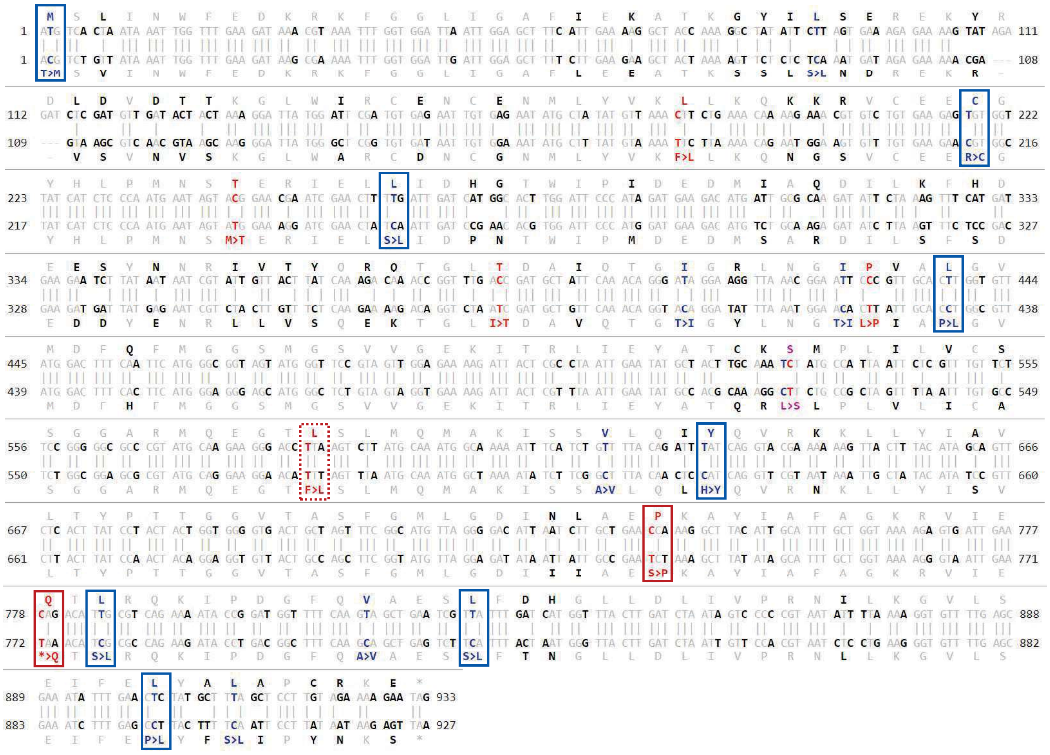

b

Edit	Percentage	Comment
accDeU-186	*21.5 %*	*5'-UTR*
accDeU-1	*54.3 %*	*5'-UTR*
accDeU2TM	89.3 %	Start codon creation
accDeU211RC	91.3 %	Also in *Adiantum capillus-veneris*
accDeU257SL	93.5 %	Also in several other taxa (I in seed plants)
accDeU431PL	93.6 %	Also in *A. capillus-veneris* and *Anthoceros angustus*
accDeC580FL	0 %	Strongly predicted by 21 of 22 references
accDeU625HY	6.4%	Variably H or Y elsewhere
accDeU657SS	1.0 %	Silent
accDeC730SP	61.0 %	Also in *A. capillus-veneris* and *Anthoceros angustus*
accDeC772*Q (UAA)	74.2 %	Also in *A. capillus-veneris* and *Anthoceros angustus*
accDeU779SL	94.1 %	Not yet reported
accDeU821SL	91.9 %	Also in *Amborella trichopoda* and *Ophioglossum vulgatum*
accDeU895PL|PS	1.2 %	Likely collateral upstream edit
accDeU896PL|PL	88.6 %	Not yet reported
accDeC+36	*8.5 %*	*3'-UTR*
accDeU+69	*4.9 %*	*3'-UTR*

Supplementary figure 2. Nomenclature extension for multiple edits affecting single codons.

The pipe symbol (|) is added for editing site labels when multiple non-silent C-to-U or U-to-C edits affecting single codons. The respective codon change considering the individual edit alone is given, as usual, at the end. The ultimate codon change outcome when taking also the neighboring non-silent change is additionally indicated before the pipe symbol. **a**. All possible ways of converting YYN (Pro, Leu, Ser and Phe) codons are shown with individual changes in the first or second codon position in the top or bottom lines, respectively. C-to-U editing is shown in blue and U-to-C editing in red. First position edits of CUR or UUR Leucine codons are silent when considered individually but are factually not when accompanied by editing in second codon position (LS|LL and LP|LL, blue shading). We name these events “primary silents”. *Vice versa*, apparent non-silent 1st position edits of CCR proline or UCR serine codons (PL|PS and SL|SP, green shading) may ultimately appear silent when the 2nd position is edited, too. We name these events “secondary silents”. For silent edits in codons also affected by non-silent edits, the codon identities before and after the non-silent edit(s) are shown before and after the underline. **b**. The example shows the exceptional case of five RNA editing events in a row causing Leu-to-Phe exchanges in two successive *nad1* codons. The respective RNA editing efficiencies are indicated. Silent sites are mostly edited with low efficiencies, as here exemplarily seen for nad1eU702LL_SS edited to only 15%. Notable exceptions are found for efficiently edited silent sites downstream of a Thr-to-Ile codon conversion in *nad4* (**c**) or for three closely spaced silent edits in the amino-terminal part of the *nad5* coding sequence (**d**).

a.

```
     / UCR PL|PS \                  / UCY PF|PS \
CCR                 UUR      CCY                   UUY
P    \ CUR PL|PL /  L        P     \ CUY PF|PL /   F

     / UUR LS|LL \                  / UUY LS|LF \
CUR                 UCR      CUY                   UCY
L    \ CCR LS|LP /  S        L     \ CCY LS|LP /   S

     / CCR SL|SP \                  / CCY SL|SP \
UCR                 CUR      UCY                   CUY
S    \ UUR SL|SL /  L        S     \ UUY SL|SF /   L

     / CUR LP|LL \                  / CUY FP|FL \
UUR                 CCR      UUY                   CCY
L    \ UCR LP|LS /  P        F     \ UCY FP|FS /   P
```

b.

```
L>S L>S
CUC CUA
UCU UCA
||| ||
||| |nad1eC704LS|LP 81%
||| nad1eU703LS|LL 93%
||nad1eU702LL_SS 15%
|nad1eC701LS|LP 91%
nad1eU700LS|LF 95%
```

c.

```
T>I  L
ACC CUA
AUU UUA
 || |
 || nad4eU46LL 79%
 |nad4eU45TT_II 92%
 nad4eU44TI 96%
```

d.

```
 P   L   L
CCC CUG CUC
CCU UUG CUU
  |  |     |
  |  |     nad5eU33LL 88%
  | nad5eU28LL 83%
 nad5eU27PP 85%
```

Supplementary figure 3. Prediction of mitochondrial RNA editing.

RNA editing sites were predicted using the "commons" function of PREPACT 3.0[90] with the selection of reference editomes shown on top (the alga *Chara vulgaris*, the liverwort *Marchantia paleacea*, the moss *Physcomitrella patens*, the lycophytes *Isoetes engelmannii* and *Selaginella moellendorffii*, the eusporangiate ferns *Ophioglossum californicum* and *Psilotum nudum* and the angiosperms *Cocos nucifera* and *Liriodendron tulipifera*). Black font indicates prediction from genomically encoded conserved codons, red font indicates known editing events in the respective reference and single letters indicate a deviating amino acid in a given reference editome. Examples are shown for the *atp9* gene (a) and the *atp6* gene (b). **a.** All 15 RNA editing events predicted for *atp9* in *Haplopteris ensiformis* were confirmed with editing efficiencies between 92% for atp9eU65AV and nearly 100% for atp9eU83SF. Few edits are shared with angiosperms, here exemplarily represented by *Cocos nucifera* and *Liriodendron tulipifera*, but many edits are shared in the lycophytes *Isoetes engelmannii* and *Selaginella moellendorffii* or the eusporangiate ferns *Ophioglossum californicum* and *Psilotum nudum*. Edit atp9eU194TM may have been missed or is undetectable in the analysis of the *O. californicum* editome. Some edits in *Haplopteris* have not yet been reported in any of the reference editomes, to our knowledge, e.g. atp9eU95TI, atp9eC116LP, atp9eC130*Q. **b.** Like in *atp9*, strongly predicted RNA edits were confirmed in *atp6* with editing efficiencies in the range between 59% for atp6eU713PL and 98% for atp6eU365PL and many are shared, particularly in the lycophyte editomes. Interestingly, this includes edit atp6eU662TI, exclusively shared with the eusporangiate editomes. Conversely, the isolated edits atp6eU382LF and atp6eU412HY in *Selaginella* and atp6eU421HY in *Isoetes* changing conserved codon identities and not reported elsewhere were also not identified in *Haplopteris ensiformis*. Additionally, we identified six unpredictable silent editing sites (see Suppl. Tab. 2) with efficiencies as low as 7% for atp6eU81II(TT) and two unpredicted edits atp6eU133HY and atp6eU245SF, likewise with only 8% and 6% efficiency, respectively. *Vice versa*, exceptions for predicted but unconfirmed sites are atp6eU197TI, atp6eC631FL and atp6eU716TI.

a

Chara vulgaris	Cocos nucifera	Isoetes engelmannii	Liriodendron tulipifera	Marchantia paleacea	Ophioglossum californicum	Physcomitrella patens	Psilotum nudum	Selaginella moellendorffii
C	A	A	A	A	A	A	A	atp9eU35AV
atp9eU65AV	atp9eU65AV	atp9eU65AV	atp9eU65AV	atp9eU65AV	atp9eU65AV	I	atp9eU65AV	atp9eU65AV
atp9eU83SF	atp9eU83SF	atp9eU83SF	atp9eU83SF	atp9eU83SF	atp9eU83SF	atp9eU83SF	atp9eU83SF	atp9eU83SF
atp9eU88PS	atp9eU88PS	atp9eU88PS	atp9eU88PS	atp9eU88PS	atp9eU88PS	atp9eU88PS	atp9eU88PS	atp9eU88PS
atp9eU92SL	atp9eU92SL	atp9eU92SL	atp9eU92SL	atp9eU92SL	atp9eU92SL	*atp9eU92SL*	atp9eU92SL	atp9eU92SL
atp9eU95TI	atp9eU95TI	atp9eU95TI	atp9eU95TI	atp9eU95TI	atp9eU95TI	atp9eU95TI	atp9eU95TI	atp9eU95TI
atp9eU100PS	atp9eU100PS	G	atp9eU100PS	atp9eU100PS	atp9eU100PS	atp9eU100PS	atp9eU100PS	atp9eU100PS
atp9eC109*R	atp9eC109*R	atp9eC109*R	atp9eC109*R	atp9eC109*R	atp9eC109*R	atp9eC109*R	atp9eC109*R	atp9eC109*R
atp9eC116LP	atp9eC116LP	atp9eC116LP	atp9eC116LP	atp9eC116LP	atp9eC116LP	atp9eC116LP	atp9eC116LP	atp9eC116LP
atp9eC130*Q	atp9eC130*Q	atp9eC130*Q	atp9eC130*Q	atp9eC130*Q	atp9eC130*Q	atp9eC130*Q	atp9eC130*Q	atp9eC130*Q
atp9eC146VA	atp9eC146VA	atp9eC146VA	atp9eC146VA	atp9eC146VA	atp9eC146VA	atp9eC146VA	atp9eC146VA	atp9eC146VA
atp9eU149TI	atp9eU149TI	atp9eU149TI	atp9eU149TI	atp9eU149TI	atp9eU149TI	atp9eU149TI	atp9eU149TI	atp9eU149TI
atp9eU185SF	atp9eU185SF	atp9eU185SF	atp9eU185SF	atp9eU185SF	atp9eU185SF	atp9eU185SF	atp9eU185SF	atp9eU185SF
atp9eU194TM	atp9eU194TM	atp9eU194TM	atp9eU194TM	atp9eU194TM	T	atp9eU194TM	atp9eU194TM	atp9eU194TM
atp9eU206SL	atp9eU206SL	atp9eU206SL	atp9eU206SL	atp9eU206SL	atp9eU206SL	atp9eU206SL	atp9eU206SL	atp9eU206SL
atp9eU221SF	-	atp9eU221SF	atp9eU221SF	atp9eU221SF	atp9eU221SF	atp9eU221SF	atp9eU221SF	atp9eU221SF

b

Chara vulgaris	Cocos nucifera	Isoetes engelmannii	Liriodendron tulipifera	Marchantia paleacea	Ophioglossum californicum	Physcomitrella patens	Psilotum nudum	Selaginella moellendorffii
atp6eU2TM	T	atp6eU2TM	T	atp6eU2TM	atp6eU2TM	atp6eU2TM	atp6eU2TM	-
atp6eU17PL	atp6eU17PL	atp6eU17PL	atp6eU17PL	atp6eU17PL	atp6eU17PL	atp6eU17PL	atp6eU17PL	atp6eU17PL
atp6eC80IT	atp6eC80IT	atp6eC80IT	atp6eC80IT	atp6eC80IT	atp6eC80IT	atp6eC80IT	atp6eC80IT	atp6eC80IT
atp6eU95SF	atp6eU95SF	atp6eU95SF	atp6eU95SF	atp6eU95SF	atp6eU95SF	atp6eU95SF	atp6eU95SF	atp6eU95SF
atp6eU104PL	atp6eU104PL	atp6eU104PL	atp6eU104PL	atp6eU104PL	atp6eU104PL	atp6eU104PL	atp6eU104PL	V
atp6eC178*Q	atp6eC178*Q	atp6eC178*Q	atp6eC178*Q	atp6eC178*Q	atp6eC178*Q	atp6eC178*Q	atp6eC178*Q	atp6eC178*Q
atp6eU197TI	atp6eU197TI	atp6eU197TI	atp6eU197TI	atp6eU197TI	atp6eU197TI	atp6eU197TI	atp6eU197TI	atp6eU197TI
atp6eU212PL	atp6eU212PL	R	atp6eU212PL	atp6eU212PL	atp6eU212PL	atp6eU212PL	atp6eU212PL	H
atp6eC229*Q	atp6eC229*Q	atp6eC229*Q	atp6eC229*Q	atp6eC229*Q	*	atp6eC229*Q	atp6eC229*Q	atp6eC229*Q
atp6eC253*Q	atp6eC253*Q	atp6eC253*Q	atp6eC253*Q	atp6eC253*Q	atp6eC253*Q	atp6eC253*Q	atp6eC253*Q	atp6eC253*Q
atp6eU271PS	L	atp6eU271PS	L	Y	atp6eU271PS	atp6eU271PS	atp6eU271PS	atp6eU271PS
atp6eU272SF	atp6eU272PL	atp6eU272SF	atp6eU272PL	Y	atp6eU272SF	atp6eU272SF	atp6eU272SF	atp6eU272SF
atp6eU293PL	atp6eU293PL	atp6eU293PL	atp6eU293PL	atp6eU293PL	atp6eU293PL	atp6eU293PL	atp6eU293PL	A
atp6eU295LF	atp6eU295LF	atp6eU295LF	atp6eU295LF	atp6eU295LF	atp6eU295LF	atp6eU295LF	atp6eU295LF	L
atp6eU298RC	atp6eU298RC	atp6eU298RC	atp6eU298RC	atp6eU298RC	atp6eU298RC	atp6eU298RC	atp6eU298RC	A
atp6eU305SL	atp6eU305SL	atp6eU305SL	atp6eU305SL	atp6eU305SL	atp6eU305SL	atp6eU305SL	atp6eU305SL	atp6eU305SL
atp6eC307*Q	atp6eC307*Q	atp6eC307*Q	atp6eC307*Q	atp6eC307*Q	atp6eC307*Q	atp6eC307*Q	atp6eC307*Q	atp6eC307*Q
atp6eC343YH	atp6eC343YH	atp6eC343YH	atp6eC343YH	atp6eC343YH	atp6eC343YH	atp6eC343YH	atp6eC343YH	atp6eC343YH
F	atp6eU359SL	atp6eU359SL	atp6eU359SL	atp6eU359SL	atp6eU359SL	atp6eU359SL	atp6eU359SL	atp6eU359SL
atp6eU365PL	atp6eU365PL	atp6eU365PL	atp6eU365PL	atp6eU365PL	atp6eU365PL	atp6eU365PL	atp6eU365PL	atp6eU365PL
I	I	I	I	I	L	I	I	atp6eU382LF
V	atp6eU389TI	atp6eU389TI	atp6eU389TI	atp6eU389TI	atp6eU389TI	atp6eU389TI	atp6eU389TI	atp6eU389TI
atp6eU404SF	atp6eU404SF	atp6eU404SF	atp6eU404SF	atp6eU404SF	atp6eU404SF	atp6eU404SF	atp6eU404SF	atp6eU404SF
atp6eC406*Q	atp6eC406*Q	atp6eC406*Q	atp6eC406*Q	atp6eC406*Q	atp6eC406*Q	atp6eC406*Q	atp6eC406*Q	atp6eC406*Q
H	H	H	N	H	N	H	N	atp6eU412HY
H	H	atp6eU421HY	H	H	S	H	H	H
atp6eU424LF	atp6eU424LF	atp6eU424LF	atp6eU424LF	atp6eU424LF	atp6eU424LF	atp6eU424LF	atp6eU424LF	atp6eU424LF
atp6eC461LP	atp6eC461LP	A	atp6eC461LP	atp6eC461LP	atp6eC461LP	atp6eC461LP	atp6eC461LP	A
atp6eC529CR	atp6eC529CR	atp6eC529CR	atp6eC529CR	atp6eC529CR	atp6eC529CR	atp6eC529CR	atp6eC529CR	atp6eC529CR
atp6eC631FL	atp6eC631FL	atp6eC631FL	atp6eC631FL	atp6eC631FL	atp6eC631FL	atp6eC631FL	I	atp6eC631FL
atp6eC635VA	G	atp6eC635VA	G	atp6eC635VA	atp6eC635VA	atp6eC635VA	atp6eC635VA	atp6eC635VA
atp6eU640LF	L	atp6eU640LF	L	atp6eU640LF	L	atp6eU640LF	L	atp6eU640LF
atp6eU644SL	F	atp6eU644SL	F	F	S	atp6eU644SL	S	atp6eU644SL
atp6eU659SL	atp6eU659SL	atp6eU659SL	atp6eU659SL	atp6eU659SL	atp6eU659SL	atp6eU659SL	atp6eU659SL	atp6eU659SL
T	T	T	T	T	atp6eU662TI	T	T	atp6eU662TI
atp6eU668SL	atp6eU668SL	atp6eU668SL	atp6eU668SL	atp6eU668SL	atp6eU668SL	atp6eU668SL	atp6eU668SL	atp6eU668SL
atp6eU674SL	atp6eU674SL	atp6eU674SL	atp6eU674SL	atp6eU674SL	atp6eU674SL	atp6eU674SL	atp6eU674SL	atp6eU674SL
atp6eC691*Q	atp6eC691*Q	atp6eC691*Q	atp6eC691*Q	atp6eC691*Q	atp6eC691*Q	atp6eC691*Q	atp6eC691*Q	atp6eC691*Q
atp6eC707IT	atp6eC707IT	atp6eC707IT	atp6eC707IT	atp6eC707IT	atp6eC707IT	atp6eC707IT	atp6eC707IT	atp6eC707IT
atp6eU713PL	atp6eU713PL	atp6eU713PL	atp6eU713PL	atp6eU713PL	atp6eU713PL	atp6eU713PL	atp6eU713PL	P
atp6eU716TI	atp6eU716TI	atp6eU716TI	atp6eU716TI	L	atp6eU716TI	atp6eU716TI	atp6eU716TI	atp6eU716TI
atp6eU728SL	atp6eU728SL	atp6eU728SL	atp6eU728SL	atp6eU728SL	atp6eU728SL	atp6eU728SL	atp6eU728SL	P

Supplementary figure 4. Mitochondrial RNA editing in *Haplopteris ensiformis:* the *rpl6-rps13-rps11* case.

The alignment exemplarily shows RNA editing heavily affecting the *rpl6-rps13-rps11* co-transcript including the removal of seven stop codons within the first 20 codons of the *rps11* reading frame. An internal PCR amplicon covers 46 editing sites from rpl6eC106*R to rps11eC136*Q. Synthesis of cDNA was primed either with random hexamers (n6) or with specific primers covering the end of *rps11* including the stop codon generation and two edits in the 3'-UTR in an edited (P+) or unedited (P-) version. The alignment displays edits (C-to-U in blue and U-to-C in red) clearly revealed in the RT-PCRs primed by the three different approaches and the results from the RNA-Seq data (RRM). Silent codon edits shown below the protein alignment were exclusively identified in the latter.

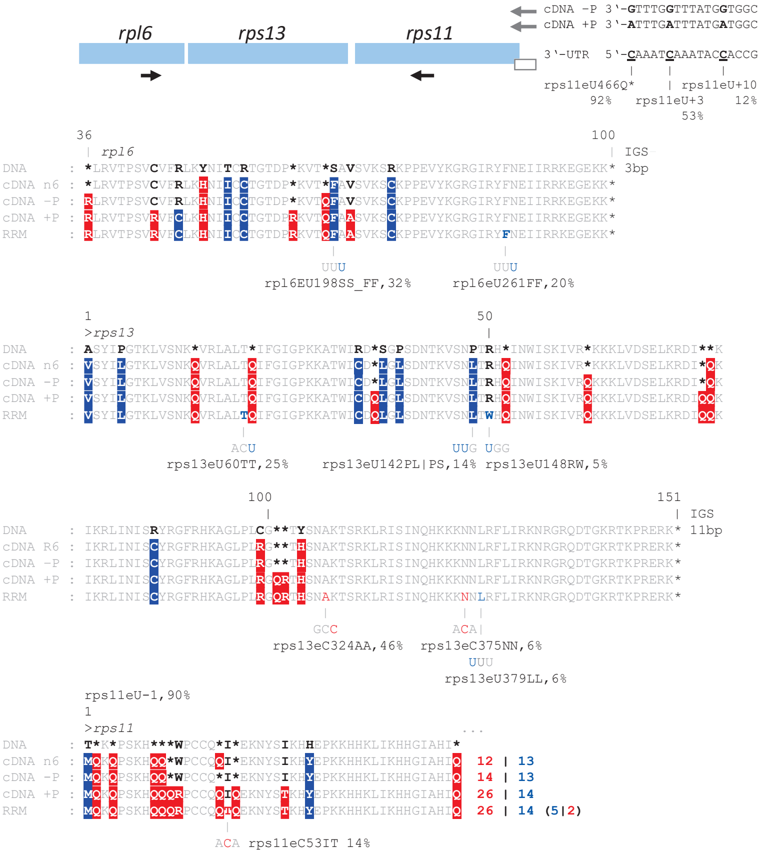

cDNA -P 3'-GTTTGGTTTATGGTGGC
cDNA +P 3'-ATTTGATTTATGATGGC
rpl6
rps13
rps11
3'-UTR 5'-CAAATCAAATACCACCG
rps11eU466Q* 92%
rps11eU+3 53%
rps11eU+10 12%
36
100
rpl6
IGS 3bp
DNA : *LRVTPSVCVFRLKYNITCRTGTDP*KVT*SAVSVKSRKPPEVYKGRGIRYFNEIIRRKEGEKK*
cDNA n6 : *LRVTPSVCVFRLKHNIICCTGTDP*KVT*FAVSVKSCKPPEVYKGRGIRYFNEIIRRKEGEKK*
cDNA -P : RLRVTPSVCVFRLKHNIICCTGTDP*KVTQFAVSVKSCKPPEVYKGRGIRYFNEIIRRKEGEKK*
cDNA +P : RLRVTPSVRVFCLKHNIICCTGTDPRKVTQFAASVKSCKPPEVYKGRGIRYFNEIIRRKEGEKK*
RRM : RLRVTPSVRVFCLKHNIICCTGTDPRKVTQFAASVKSCKPPEVYKGRGIRYFNEIIRRKEGEKK*
UUU
rpl6EU198SS_FF,32%
UUU
rpl6eU261FF,20%
1
50
>rps13
DNA : ASYIPGTKLVSNK*VRLALT*IFGIGPKKATWIRD*SGPSDNTKVSNPTRH*INWISKIVR*KKKLVDSELKRDI**K
cDNA n6 : VSYILGTKLVSNKQVRLALTQIFGIGPKKATWICD*LGLSDNTKVSNLTRHQINWISKIVR*KKKLVDSELKRDI*QK
cDNA -P : VSYILGTKLVSNKQVRLALTQIFGIGPKKATWICD*LGLSDNTKVSNLTRHQINWISKIVRQKKKLVDSELKRDI*QK
cDNA +P : VSYILGTKLVSNKQVRLALTQIFGIGPKKATWICDQLGLSDNTKVSNLTRHQINWISKIVRQKKKLVDSELKRDIQQK
RRM : VSYILGTKLVSNKQVRLALTTQIFGIGPKKATWICDQLGLSDNTKVSNLTWHQINWISKIVRQKKKLVDSELKRDIQQK
ACU
rps13eU60TT,25%
UUG UGG
rps13eU142PL|PS,14%
rps13eU148RW,5%
100
151
IGS 11bp
DNA : IKRLINISRYRGFRHKAGLPLCG**TYSNAKTSRKLRISINQHKKKNNLRFLIRKNRGRQDTGKRTKPRERK*
cDNA R6 : IKRLINISCYRGFRHKAGLPLRG**THSNAKTSRKLRISINQHKKKNNLRFLIRKNRGRQDTGKRTKPRERK*
cDNA -P : IKRLINISCYRGFRHKAGLPLRG**THSNAKTSRKLRISINQHKKKNNLRFLIRKNRGRQDTGKRTKPRERK*
cDNA +P : IKRLINISCYRGFRHKAGLPLRGQRTHSNAKTSRKLRISINQHKKKNNLRFLIRKNRGRQDTGKRTKPRERK*
RRM : IKRLINISCYRGFRHKAGLPLRGQRTHSNAKTSRKLRISINQHKKKNNLRFLIRKNRGRQDTGKRTKPRERK*
GCC
rps13eC324AA,46%
ACA
rps13eC375NN,6%
UUU
rps13eU379LL,6%
rps11eU-1,90%
1
>rps11
...
DNA : T*K*PSKH***WPCCQ*I*EKNYSIKHHEPKKHHKLIKHHGIAHI*
cDNA n6 : MQKQPSKHQQ*WPCCQQI*EKNYSIKHYEPKKHHKLIKHHGIAHIQ 12 | 13
cDNA -P : MQKQPSKHQQ*WPCCQ*I*EKNYSIKHYEPKKHHKLIKHHGIAHIQ 14 | 13
cDNA +P : MQKQPSKHQQQRPCCQQIQEKNYSTKHYEPKKHHKLIKHHGIAHIQ 26 | 14
RRM : MQKQPSKHQQQRPCCQQTQEKNYSTKHYEPKKHHKLIKHHGIAHIQ 26 | 14 (5|2)
ACA rps11eC53IT 14%

Supplementary figure 5. Electrophoretic gels of PCR experiments on *Haplopteris ensiformis* mitochondrial DNA used in figure 4 and 7

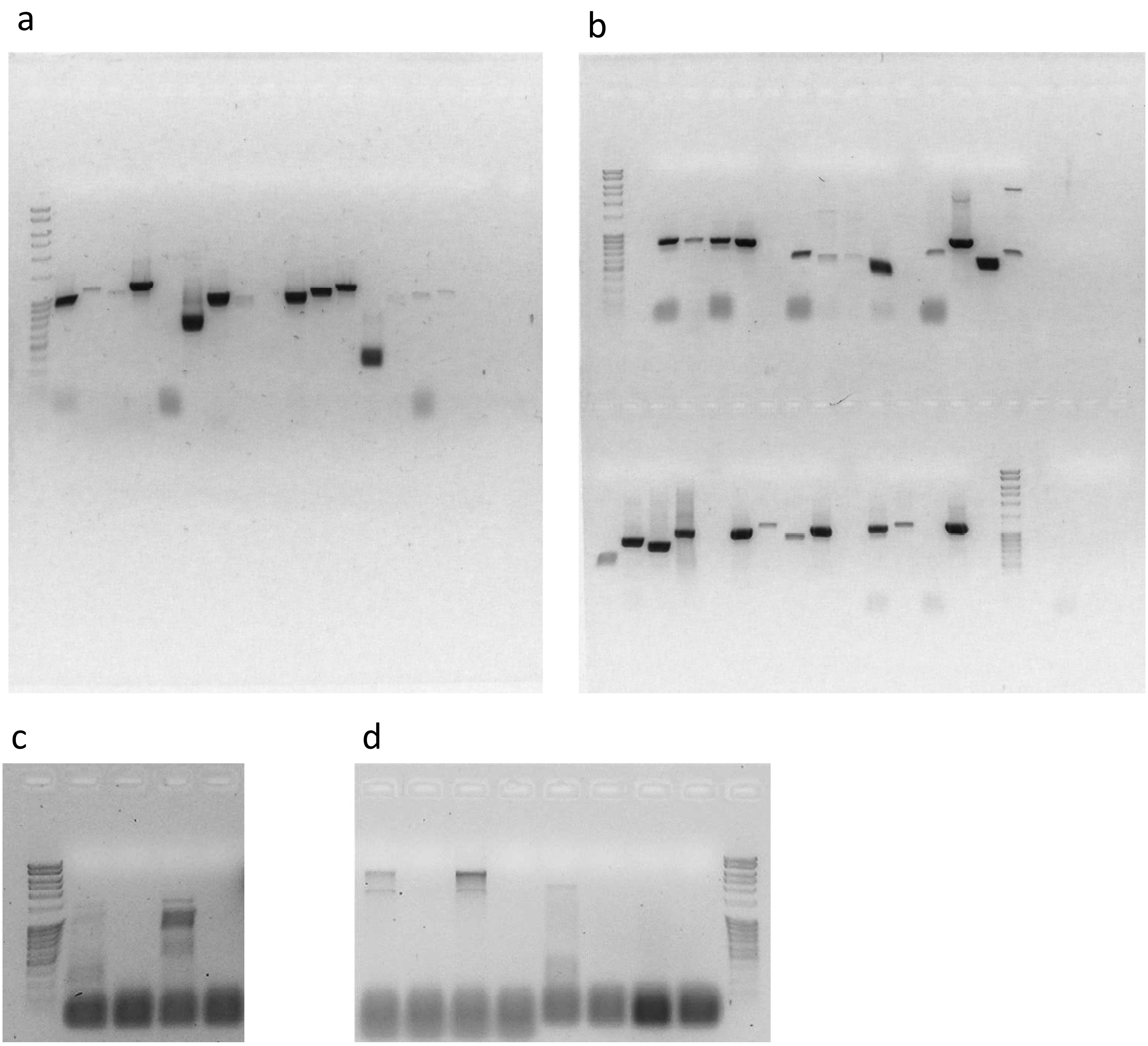
a
b
c
d
