# Supplementary material for: Rickettsial DNA and a trans-splicing rRNA group I intron in the unorthodox mitogenome of the fern Haplopteris ensiformis
Source: Commun Biol. 2023 Mar 20;6:296. doi: 10.1038/s42003-023-04659-8 (PMC10027690; doi:10.1038/s42003-023-04659-8)
Supplement: Supplementary file 3 — Description of Additional Supplementary Files [file 42003_2023_4659_MOESM3_ESM.pdf]

Description of Additional Supplementary Files

File name: Supplementary Data 1
Description: Chloroplast RNA editing in Haplopteris ensiformis.

File name: Supplementary Data 2
Description: Mitochondrial RNA editing in Haplopteris ensiformis.

File name: Supplementary Data 3
Description: Annotated features in the Haplopteris ensiformis mitogenome chromosomes.

File name: Supplementary Data 4
Description: Bacterial protein sequence similarities in the Haplomitrium ensiformis mitogenome.

File name: Supplementary Data 5
Description: Bacterial nucleotide sequence similarities in the Haplopteris ensiformis mitogenome.

File name: Supplementary Data 6
Description: Sequencing, assembly and mapping read statistics of the Haplopteris ensiformis organellar genome project PRJNA862965.

File name: Supplementary Data 7
Description: List of all primers/oligonucleotides used for PCR experiments.
