# Supplementary material for: Rickettsial DNA and a trans-splicing rRNA group I intron in the unorthodox mitogenome of the fern Haplopteris ensiformis
Source: Commun Biol. 2023 Mar 20;6:296. doi: 10.1038/s42003-023-04659-8 (PMC10027690; doi:10.1038/s42003-023-04659-8)
Supplement: Supplementary file 7 — Supplementary Data 4 [file 42003_2023_4659_MOESM7_ESM.docx]

### Supplementary data 4. Bacterial protein sequence similarities in the *Haplomitrium ensiformis* mitogenome.

Shown are the top similar alignments indicating length of the native protein entry, the numbers of identical and similar residues and total alignment length separated by slashes followed by percentages of identical and similar residues, respectively.

#### [A](#Suppl_Fig_1A_1st_reference). HscA-RmuC

**Fe-S protein assembly chaperone HscA [*Caedimonas varicaedens*], Part 1**
Sequence ID: [WP_062141505.1](https://www.ncbi.nlm.nih.gov/protein/WP_062141505.1?report=genbank&log$=protalign&blast_rank=1&RID=CZK231NX014) Length: 595, 134/149/163, 83/91 %

Query 1 RSAQVPIDQIKGIVLVGGATRIPLVLKKVKQFFNQTPLTDINPDEVVALGAALQAETLTQ 180

R AQ+ +DQIKG+VLVGGATRIPLV +KV++FF Q PLT+INPDEVVALGAALQAE LT+

Sbjct 305 RDAQISLDQIKGVVLVGGATRIPLVRQKVERFFEQVPLTNINPDEVVALGAALQAEALTE 364

Query 181 GSNRllldvsplslglETYGGIVEKIIPRNSPIPATYEQDFTTFQDNQTGISLHVVQGER 360

GS RLLLDV+PLSLGLETYGG+VEKII RNSPIPA++ QDFTTFQDNQTG+SLH+VQGER

Sbjct 365 GSQRLLLDVAPLSLGLETYGGVVEKIILRNSPIPASHWQDFTTFQDNQTGMSLHIVQGER 424

Query 361 ELAHDCPSLAHFELRGIPPMKAGIARIRVTFTIDADGILTVS 486

EL DC SLAHFELRGIP MKAG+ARIRVTFTIDADGILTVS

Sbjct 425 ELVQDCRSLAHFELRGIPLMKAGMARIRVTFTIDADGILTVS 466

**Fe-S protein assembly chaperone HscA [*Caedimonas varicaedens*], Part 2**Sequence ID: [WP_062141505.1](https://www.ncbi.nlm.nih.gov/protein/WP_062141505.1?report=genbank&log$=protalign&blast_rank=1&RID=CZK231NX014) Length: 595, 70/96/119, 59/80 %

Query 802 STGIHQSIEVKPSYGLSEEDLLKIIEESHVYGYLDMEERLLRETRVEAERLFHAVregle 981

+TGIHQ I+VKPSYGL+EEDLL++IEESH + DM+ERLLRE RVEAERL HAV+E L+

Sbjct 471 TTGIHQYIDVKPSYGLTEEDLLRMIEESHSHARSDMDERLLREARVEAERLLHAVKEALK 530

Query 982 ddadlleenerrGIEQAINELATVLNSSHRGLIKEKIDEIKKMTHSWAERRLNRSMSKA 1158

+D+DLLEE+E+ ++Q L +VLNS R I+ K + ++++T+SWAERR+N++M KA

Sbjct 531 NDSDLLEESEKIEVKQEAEALISVLNSVKRDDIERKTERLRQVTNSWAERRMNQAMRKA 589

**DNA recombination protein RmuC [*Caedimonas varicaedens*], Part 1**
Sequence ID: [WP_062141503.1](https://www.ncbi.nlm.nih.gov/protein/WP_062141503.1?report=genbank&log$=protalign&blast_rank=2&RID=CZK231NX014) Length: 475, 111/128/142, 78/90 %

Query 1198 MNIQQYEFAQLLIGIALGGSVGFLWMLRIKKLAHRTFDNLNEEMRNLRERFILSEDKAQR 1377

MNIQQYE AQ+L G LGGS+GF +++R+K+LA TFD LNEEM+N+RER ILSEDKAQR

Sbjct 1 MNIQQYELAQVLTGAILGGSIGFFFIMRVKRLAQLTFDRLNEEMQNVRERLILSEDKAQR 60

Query 1378 HDNLVEDKNQLFERLSEARTKHAEVEVLLIQERKQLQEKFKVIEDARQELSNAFQALSAQ 1557

++ + E+KNQLFERLS+AR K+AEV+VLL QERKQ QEKFKVIE+ARQELSNAFQALSAQ

Sbjct 61 YETMTEEKNQLFERLSDARAKYAEVDVLLGQERKQFQEKFKVIEEARQELSNAFQALSAQ 120

Query 1558 ALERNNRSFLDLAKSTLEKFHE 1623

ALERNNRSFLDLAKSTLEKFHE

Sbjct 121 ALERNNRSFLDLAKSTLEKFHE 142

#### [B](#Suppl_Fig_1B_1st_reference). DNA recombination protein RmuC [*Caedimonas varicaedens*], Part 2

Sequence ID: [WP_062141503.1](https://www.ncbi.nlm.nih.gov/protein/WP_062141503.1?report=genbank&log$=protalign&blast_rank=1&RID=9941M5FE016) Length: 475, 188/193/200, 94/96%

Query 1 AKSTLEKFHETAKGDLQLKEKAISELVSPIRESLHNVDQKIGEIEKIRLSAYEVLRHQVG 180

AKSTLEKFHETAKGDLQLKEKAISELVSPIRESLHNVDQKIGEIEKIRLSAYEVLRHQVG

Sbjct 133 AKSTLEKFHETAKGDLQLKEKAISELVSPIRESLHNVDQKIGEIEKIRLSAYEVLRHQVG 192

Query 181 DLILSQKELRLETANLVKALRTPHVRGRWGEIQLRRVVEMSGMSIHCDFIEQKTLEGEEV 360

DLILSQKELR ETANLVKALRTPHVRGRWGE+QLRRVVEMSGMS+HCDFIEQ TLEGEE+

Sbjct 193 DLILSQKELRSETANLVKALRTPHVRGRWGEMQLRRVVEMSGMSVHCDFIEQTTLEGEEI 252

Query 361 KLRPDMIVRLPGGKRLIIDAKAPLSAYLDALEAKEEVLRQDLLKDHARQVRTHILSLSTH 540

KLRPDMIVRLPGGKRLIIDAKAPL+AYLDALEAKEE LRQD LKDHARQVRTHILSLST

Sbjct 253 KLRPDMIVRLPGGKRLIIDAKAPLAAYLDALEAKEETLRQDFLKDHARQVRTHILSLSTR 312

Query 541 AYWNQFYKSGETPEFVVMFL 600

AYW+Q KSGETPEFVVMFL

Sbjct 313 AYWDQLSKSGETPEFVVMFL 332

#### C. Hypothetical protein BGO77_01970 [*Caedibacter sp. 37-49*] Transglycosylase SLT domain-containing protein

Sequence ID: [OJX11001.1](https://www.ncbi.nlm.nih.gov/protein/OJX11001.1?report=genbank&log$=protalign&blast_rank=1&RID=D03B7GTN014) Length: 642, 136/201/308, 44/65 %

Query 1 ALSHFKKLEPVVKTPISCSRVGYWLGRTYEAMGKAKEARTSYQKAAQYKGTFYGQKAFSK 180

A HF +L V +P S S+ YW GR +A +EA YQKAA Y T+YGQ A

Sbjct 334 AFHHFNQLYQKVTSPYSRSKAAYWAGRAAQAKKLPQEAHLWYQKAAYYPATYYGQLALKA 393

Query 181 LGHTDKEIILETLRFTPAQQAKFESHKAVKLIRLLAKEEIDEHILSFAYVFAKQTASPLE 360

L + + LE+L F ++ KFE+H+ V+ ++LL K + + +LSF Y++A QT+S E

Sbjct 394 L-NKPQTFRLESLTFPISEVRKFENHEFVRAVKLLHKAGLTDELLSFGYMYA-QTSSSHE 451

Query 361 RKQILALVHELAPHYGVEIAQVIAPYQSTLYSEAFPRLKPIYLKHMEKVDAALAHAVIRK 540

+L+L +AP + V +A ++ +T Y EA+P++ ++ A+ HA+IRK

Sbjct 452 ALMMLSLASTIAPQFAVGMADSMSILHNTHYKEAYPQVCRDNNSAQACLEDAMIHALIRK 511

Query 541 ESKFNPKIVSWAGAQGLMQLMPETAQLMADQCGVTCSEKQLLTDPLLNVKLGTLYLKEQL 720

ESKFNP+ S AGA G+MQ++P TA+++A + GV +E +L +D N+ +GT YLKE+L

Sbjct 512 ESKFNPRAKSEAGACGMMQVVPSTAKMVAQKMGVHFNEARLKSDMNYNMLIGTAYLKERL 571

Query 721 EKYDHSFPLTLASYNAGPGTVSRWLDRFPDPRHPSINTIDWIEVLPYSETRDYIHRVLEN 900

+ Y S LTLA+YNAGPG+V +W++R+ DPR P ++TIDW+E +PYSETR+YI RV+EN

Sbjct 572 QDYQGSIVLTLAAYNAGPGSVKKWIERYGDPRDPKVDTIDWVEKIPYSETRNYIQRVMEN 631

Query 901 YTIYKAIL 924

Y IY+AI

Sbjct 632 YIIYQAIF 639

#### G. SerS, Serine tRNA ligase [*Caedimonas varicaedens*]

Sequence ID: [WP_062139602.1](https://www.ncbi.nlm.nih.gov/protein/WP_062139602.1?report=genbank&log$=protalign&blast_rank=1&RID=D05EYUBN014) Length: 429, 268/297/317, 85/93 %

Query 1 EGQTDADNMEVRRWGSPRTFDFTPLPHYEIGEKLQEMDFENAAKIAGARFLILKGNLARL 180

EG+TD++N+E+R+WG+PRTFDFTPLPHYEIGEKLQ MDF+ A KI+GARF++LKG LARL

Sbjct 113 EGRTDSENIEIRKWGTPRTFDFTPLPHYEIGEKLQGMDFDTATKISGARFVVLKGALARL 172

Query 181 ERALGQFMLDLHTQ*FGYQEVSPPLLVRDETVYGVGQLPKFKENLFQTTDGRWLISTAEV 360

ERALGQFMLDLHTQ FGYQEVSPPLLVRD+ VYGVGQLPKF+E+LFQT+DGRWLISTAEV

Sbjct 173 ERALGQFMLDLHTQEFGYQEVSPPLLVRDDAVYGVGQLPKFREDLFQTSDGRWLISTAEV 232

Query 361 SLTNLVREKIVDEGVLPLRFTALTPCFRSEAGSAGRDTRGMIRQHQFHKVELVSIVHPDA 540

SLTNLVRE+I+DEG LPLRFTA TPCFRSEAG+AGRDTRGMIRQHQFHKVELVSIVHPDA

Sbjct 233 SLTNLVRERILDEGSLPLRFTAFTPCFRSEAGAAGRDTRGMIRQHQFHKVELVSIVHPDA 292

Query 541 AEEEHQRMVNAAETVLQLLKIPYRIMLLSSGDTGTQSRRTYDLDVWLPG*NLYREISSCS 720

AE EHQRMVNAAETVLQ L+IPYR+MLLS GDT SRRTYDL+VWLPG N+YREISSCS

Sbjct 293 AETEHQRMVNAAETVLQRLEIPYRVMLLSCGDTSGASRRTYDLEVWLPGENMYREISSCS 352

Query 721 NCGDYQARRMQARFRPKNGAGNKNSPEFVFTINGSGVAVGRALIAVIENSQNSDGSITIP 900

NCGDYQARRMQARFRPKN G+KN+ EFVFT+NGSGVAVGRALIAVIEN QNSDGS+TIP

Sbjct 353 NCGDYQARRMQARFRPKNDTGSKNTTEFVFTLNGSGVAVGRALIAVIENYQNSDGSMTIP 412

Query 901 DILRPYMKGMERISLHD 951

+ LRPYMKGM+RIS+HD

Sbjct 413 EALRPYMKGMKRISIHD 429

#### SurE, 5'/3'-nucleotidase [*Caedimonas varicaedens*]

Sequence ID: [WP_062139600.1](https://www.ncbi.nlm.nih.gov/protein/WP_062139600.1?report=genbank&log$=protalign&blast_rank=3&RID=D05EYUBN014) Length: 258, 246/252/258, 95/97 %

Query 947 MIKIMDQKPRILISNDDGIHAPGLKVLEDIASTITDDIWIVAPEWEQSGAAHFLSIGRPL 1126

MIKIM QKPRILISNDDGIHA GLKVLEDIAS++TDDIWIVAPEWEQSGAAH LSIGRPL

Sbjct 1 MIKIMHQKPRILISNDDGIHAAGLKVLEDIASSMTDDIWIVAPEWEQSGAAHSLSIGRPL 60

Query 1127 RFRELGPKRYTVNGTPTDCVMIAVNKLMTACRPDLMLSGVNHGANLGEDVTYSGTVAAAM 1306

RFRELGPKRYTVNGTPTDCVMIAVNKLMT CRPDLMLSGVNHGANLGEDVTYSGTVAAAM

Sbjct 61 RFRELGPKRYTVNGTPTDCVMIAVNKLMTECRPDLMLSGVNHGANLGEDVTYSGTVAAAM 120

Query 1307 EATLLGIPAIALSQSLITDHENGYDPARHYGPILIKKLISQTWPRHVLININFPDISVDK 1486

EATLLGIPAIALSQSL+TDHEN YDPARHYGP+L+KKLISQTWPRHVLININFPDISVDK

Sbjct 121 EATLLGIPAIALSQSLLTDHENAYDPARHYGPLLVKKLISQTWPRHVLININFPDISVDK 180

Query 1487 IQGARVVHQGLRNINDNLVKWKDPHGKPFFWIGGNRDDSPTEEETDLEAIHQGSISITPL 1666

IQGARVV QGLRNINDNLVKWKDPHGKPFFWIGGNRDDSPTEEETDLEAIHQG+ISITPL

Sbjct 181 IQGARVVRQGLRNINDNLVKWKDPHGKPFFWIGGNRDDSPTEEETDLEAIHQGAISITPL 240

Query 1667 HLDLTHHSTLETLKLAFI 1720

HLDLTHHSTLETLKLAFI

Sbjct 241 HLDLTHHSTLETLKLAFI 258

#### J. MurA, UDP-N-acetylglucosamine 1-carboxyvinyltransferase [Rickettsiaceae bacterium]

Sequence ID: [RYE05808.1](https://www.ncbi.nlm.nih.gov/protein/RYE05808.1?report=genbank&log$=protalign&blast_rank=39&RID=D0CNY9V7014)Length: 422, 287/341/420, 68/81 %

Query 1278 MDSiiikggkpligKVNISGAKNaalpiitaallAEGNLNLINIPKLTDIYTMKTLLQNH 1099

MDSI+IKGG PL G V I+GAKNAALPI+ AALL + L NIPKLTD+ TM +LL++H

Sbjct 1 MDSIVIKGGTPLQGIVKINGAKNAALPIMAAALLTADRIVLTNIPKLTDVETMNSLLRHH 60

Query 1098 GILIDIKDLGDYYALSINSNKVDNFVAPYDIVRKMRASIWVLGPLLARFGKAQVSLPGGC 919

G ++ D + L I+ +K+ +F+APY+IVRKMRASIWVLGPLLARFG+A++SLPGGC

Sbjct 61 GAIVSSYDQAESLELHIDCSKITDFIAPYEIVRKMRASIWVLGPLLARFGEAKISLPGGC 120

Query 918 AIGARQVDLHIDVLRAMGADIEITHGYIKAKSKGRLKGVHFNFNKISVGATINAIMAATL 739

AIGARQVD+HI VL AM A IEI GYIKA SKG+LKG HFNF++ISVGATI AI+AA+L

Sbjct 121 AIGARQVDMHISVLEAMNAHIEIEQGYIKATSKGQLKGTHFNFHRISVGATITAILAASL 180

Query 738 AKGETVLLNCAREPEIIDLCKCLTAMGANIDGDSTGEIKITGVESLKGANYKVMPDRIEA 559

A+GET L NCA+EPEI+DLCK L MGA I G T EIKI GV SLKG Y VMPDRIEA

Sbjct 181 AEGETSLTNCAKEPEIVDLCKYLCKMGAKIKGAGTAEIKIMGVSSLKGVTYDVMPDRIEA 240

Query 558 GTYMIAAAITKGKLDILGIDYHIIENLGLKLIDAGVEVSPLENGVRLKYSGNIKSVDIQT 379

GTYMIAAAITKG+LDI+GIDY I+ENL LKL+++G V+ +G+ +++SG IK DI T

Sbjct 241 GTYMIAAAITKGELDIVGIDYKIVENLVLKLLESGTIVTRTNSGINVRHSGVIKPTDINT 300

Query 378 EAYPGFSTDLQAQFMSLMTLAEGAAVITENIF*NRFMHIPELCRMGANITISGHSAMIRG 199

E YPGF+TDLQAQFMSL+T+A+GA+VITENIF NR+MH+PEL RMGANI I H+A+I+G

Sbjct 301 EEYPGFATDLQAQFMSLLTIADGASVITENIFENRYMHVPELNRMGANIIIKEHNAIIKG 360

Query 198 VEHLSGAEVMASDlrasvslvlaglaAKGETIIRRVYHLDRGYQQLEQKLSHCGADIKRI 19

V++L GAEVMASDLRAS L+LA LAA GET I RVYHLDRGYQ E KLS CGADI RI

Sbjct 361 VKYLKGAEVMASDLRASSCLILAALAATGETRIGRVYHLDRGYQAFEHKLSSCGADIMRI 420

#### Acyl-CoA carboxylase subunit beta [Rickettsiaceae bacterium]

Sequence ID: [RYE05809.1](https://www.ncbi.nlm.nih.gov/protein/RYE05809.1?report=genbank&log$=protalign&blast_rank=14&RID=D0CNY9V7014)Length: 514, 453/485/514, 88/94 %

Query 1503 MNQTILPDQDLLEEKRSIARAGGGHDRIALQHKKGKLTARQRVEAFLDPDSFEETGMFVE 1682

MNQ +L QDLLEEKRSIARAGGG +RIALQHKKG+LTAR+R+E LDPDSFEETGMFVE

Sbjct 1 MNQNVLQGQDLLEEKRSIARAGGGLERIALQHKKGRLTARERIEVLLDPDSFEETGMFVE 60

Query 1683 HRCDNFGMKDKKFAGDGVVTGHGTINGRLVFIYSQDFTVLGGSLGEYHAKKICNVIDSAL 1862

HRCDNFGMKDKK AGDGVVTGHGTINGRLVFIYSQDFTVLGGSLGEYHAKKIC+V+DSAL

Sbjct 61 HRCDNFGMKDKKTAGDGVVTGHGTINGRLVFIYSQDFTVLGGSLGEYHAKKICSVVDSAL 120

Query 1863 QVGAPVIGINDSGGARIQEGVDALGGYGELFQRNVIASGVIPQITLIMGPCAGGAVYSPA 2042

Q GAPVIGINDSGGARIQEGVDALGGYGELFQRNV+ASGVIPQI+LIMGPCAGGAVYSPA

Sbjct 121 QAGAPVIGINDSGGARIQEGVDALGGYGELFQRNVLASGVIPQISLIMGPCAGGAVYSPA 180

Query 2043 LTDFIFMVRDSSYMFVTGPDVVKTVTGEEVSQEKLGGARMHTTKSGVADLAFKNDIEALL 2222

LTDFIFMVRDSSYMFVTGP+VVK VTGEEVSQEKLGGAR+HTTKSG+ADL+FKNDIEALL

Sbjct 181 LTDFIFMVRDSSYMFVTGPEVVKAVTGEEVSQEKLGGARIHTTKSGIADLSFKNDIEALL 240

Query 2223 ETRRFFNFLPLSNRAPLPTRYTKDPADRVDMSLNTLVPLAANKSYDMKELIQRIVDEG*F 2402

ETRR FNFLPLSNRAPLP+R TKDPADRVDMSLNTLVP NKSYDMKELI+RIVDEG F

Sbjct 241 ETRRLFNFLPLSNRAPLPSRCTKDPADRVDMSLNTLVPNVPNKSYDMKELIKRIVDEGDF 300

Query 2403 FELQPDFAKNILIGFGYMEGRSIGFVANQPLYLAGCLDINASRKAARFIRFCDAFNIPIV 2582

FEL +FAKNI+IGFGYMEGR +GFVANQPLYLAGCLDIN+SRKAARFIRFCDAFNIPI+

Sbjct 301 FELHAEFAKNIIIGFGYMEGRPVGFVANQPLYLAGCLDINSSRKAARFIRFCDAFNIPII 360

Query 2583 SLVDVPGFLPGTAQEHDGIIKHGAKLLYAYAEATVPKITVITRKAYGGAYIVMNSKHLRG 2762

SLVDVPGFLPGT QE++G+IKHGAKLLYAYAEATVPKITVITRKAYGGAYIVMNSKHLRG

Sbjct 361 SLVDVPGFLPGTNQEYNGLIKHGAKLLYAYAEATVPKITVITRKAYGGAYIVMNSKHLRG 420

Query 2763 DVNYAWVNPEIAVMGAEGAAEIIFKEDCKDSDLKKKKIQEYKDTVTSLFVAASRGYLDDI 2942

DVNYAW+N EIAVMGAEGAAEIIFK +CKD + KK IQEYKDT+TS F+AASRGY+DDI

Sbjct 421 DVNYAWINSEIAVMGAEGAAEIIFKNECKDPEQKKILIQEYKDTITSPFIAASRGYIDDI 480

Query 2943 IKPQNTRWRICKALNFLQGKNTQMPWKKHDNLPL 3044

IKPQNTRWR+CKALNFL+ K Q+PWKKHDNLPL

Sbjct 481 IKPQNTRWRLCKALNFLREKKVQVPWKKHDNLPL 514

#### Acetyl/propionyl/methylcrotonyl-CoA carboxylase subunit alpha [Rickettsiaceae bacterium]

Sequence ID: [RYE05810.1](https://www.ncbi.nlm.nih.gov/protein/RYE05810.1?report=genbank&log$=protalign&blast_rank=1&RID=D0CNY9V7014) Length: 665, 571/626/665, 86/94 %

Query 3077 MNKPLFDKVLIANRGEISLRIMRSLKKMGIKSVAVYSEADTGSKHVQEADEAYYVGNSPA 3256

M+KPLFDKVLIANRGEI+LRIMRSLKKMGIKSVA+YSEADT S HVQ ADEAYYVGNSPA

Sbjct 1 MSKPLFDKVLIANRGEIALRIMRSLKKMGIKSVAIYSEADTNSMHVQYADEAYYVGNSPA 60

Query 3257 TESYLSIKNIVNAARASGAQAVHPGYGFLAENFNFANILKREGITLIGPSAQAIKQMGDK 3436

TESYLSIKNI+NA RASGAQAVHPGYGFLAEN NFA+ LKREGITLIGPSA AIKQMGDK

Sbjct 61 TESYLSIKNIINAIRASGAQAVHPGYGFLAENANFASALKREGITLIGPSAGAIKQMGDK 120

Query 3437 IEAKKIATEAGVTTVPGYMGIIGNVNQAISIAEEIGFPVIVKaaaggggrgMRVVKNSSE 3616

IEAKKIATEAGVTTVPGYMGII N+ QAISIAEEIGFPVIVKAAAGGGGRGMRVVKNS+E

Sbjct 121 IEAKKIATEAGVTTVPGYMGIIRNITQAISIAEEIGFPVIVKAAAGGGGRGMRVVKNSAE 180

Query 3617 MAAAFESAKLEAENSFNDGRVFIEKLIENPRHIEIQLIADQFGNAVCLGERECSIQRHHQ 3796

MA AFESAK+EAENSF+DGRVFIE+LIE+PRHIEIQL+ADQFGNAVCLGERECSIQRHHQ

Sbjct 181 MAQAFESAKIEAENSFDDGRVFIERLIESPRHIEIQLLADQFGNAVCLGERECSIQRHHQ 240

Query 3797 KIIEEAPSSFITEEIRQKMYAEVIALTHKVGYYSAGTVEFIMDPDKHYYFMEMNTRLQVE 3976

KIIEEAPS FITEEIR++MYAEVIAL++KVGYYSAGTVEFIMD K YYFMEMNTRLQVE

Sbjct 241 KIIEEAPSVFITEEIRKQMYAEVIALSNKVGYYSAGTVEFIMDSKKQYYFMEMNTRLQVE 300

Query 3977 HPVTELVTGIDIVEEMIKIAAGEKLSFTQDDIKLKGWAIESRICSEDPTRGFLPSSGRIT 4156

HPVTELVTGIDI+EEMIKIAAGEKLSFTQ+DIK+KGWAIE+RICSEDPTRGFLPSSGRIT

Sbjct 301 HPVTELVTGIDIIEEMIKIAAGEKLSFTQEDIKIKGWAIEARICSEDPTRGFLPSSGRIT 360

Query 4157 GYAEPLKSPHIRIDSGVSIGGEVSMFYDPMIAKLCTYHDTREQAIEIMQTALSSYVIQGI 4336

YAEPLKSP+IR+DSGV GGEVSMFYDPMIAKLCTYHD R QAIE M++ALSSYVIQGI

Sbjct 361 EYAEPLKSPNIRVDSGVGAGGEVSMFYDPMIAKLCTYHDDRAQAIECMRSALSSYVIQGI 420

Query 4337 SHNISFLEAVMSHPRFISGNINTGFIAEEYPAGFSGASLTSEITDVFLATAIYTYITEQK 4516

SHNISFLEAV+SH RFI GNINTGFIAEEYPAGFSGA+LTSEITDVFL+TAI+ YITEQK

Sbjct 421 SHNISFLEAVISHQRFIDGNINTGFIAEEYPAGFSGATLTSEITDVFLSTAIFAYITEQK 480

Query 4517 RAASIEGQMVDQASKIGTRWVVSIDDRLFPVLIKPVPDGYNIRQGYTRIYIRSNWNLGSH 4696

RA+SI GQ++DQ S IGTRWVVSIDD+LFPVLIKPV +GYNIRQGYTRIYIRSNW++GSH

Sbjct 481 RASSISGQIIDQVSNIGTRWVVSIDDKLFPVLIKPVQEGYNIRQGYTRIYIRSNWHIGSH 540

Query 4697 LFSAVVNGRKVNVKIENISTGYRLTHSGITVKTFVRSPLMSELESIMPVKLQLDDLTELT 4876

LFSA +NGRKVNVKIE+I TGY+LTHSGITVK +VRSP MSELES+M K+ ++D ELT

Sbjct 541 LFSATINGRKVNVKIESILTGYKLTHSGITVKAYVRSPRMSELESVMLGKITVNDQAELT 600

Query 4877 APLAGQIIAVKVQEGDEVIVSQEIMILTAMKMENIILAECAGKIAKIFVKDKDQVSAGQV 5056

APLAGQIIAVK++E +EV+V QE+++LTAMKMENIILAE +GKIAKIF+++KDQVSAGQV

Sbjct 601 APLAGQIIAVKIKEQEEVVVGQELIVLTAMKMENIILAERSGKIAKIFIQEKDQVSAGQV 660

Query 5057 LLEFE 5071

L+EFE

Sbjct 661 LMEFE 665

#### A. C15:645..2066, virB8 and virB9

virB8 family protein [Rickettsia endosymbiont of Culicoides newsteadi]
Sequence ID: [WP_094649370.1](https://www.ncbi.nlm.nih.gov/protein/WP_094649370.1?report=genbank&log$=protalign&blast_rank=1&RID=8VTAFGF9014) Length: 231 133/177/231 (58/76 %)

Query 645 MEPILNSVQEYIKFGEYFKDAKKWYNFKYIYPLSQRSFLLIIRSILLTLFIGLALNISYL 824

M+ + +SVQEYIK GEYF DA++WYNF+Y+YPL RSFLLI + L LF+G+ ++++ L

Sbjct 1 MDKVSSSVQEYIKSGEYFVDARRWYNFEYLYPLVHRSFLLIFTVVFLALFLGVVVSVNSL 60

Query 825 FPMVNQVRYLLQAQS*SSANIIRADLVKNDPLASIADIMVRNYLIHREKYDYDNLKGQFI 1004

P V QVRY + A+S SA I A+ +K++ + SIADIM++NY+IHRE YDYD L+ QF+

Sbjct 61 LPTVRQVRYAISAESLKSATITNANHIKSNAINSIADIMIKNYVIHRESYDYDFLRPQFM 120

Query 1005 FIQNNSTIIVFRKLFNYMNIDNPSSPVMCYQQYTRRSINILSFTYKKADKAEIIFSSIAK 1184

FIQN+ST I+FR+ N+MNIDN SPVM YQ+ RRS+NILS Y K +KAE+IF+S+AK

Sbjct 121 FIQNSSTRIIFRQFANFMNIDNSLSPVMRYQRSLRRSVNILSVVYHKNNKAEVIFTSVAK 180

Query 1185 TAGSKIFENMVWQATINFDIDKINLGLPSDSRFNFAVTNYCLKILQDKMSK 1337

+ + I ENMVWQATINF+ID IN+ LP +S FNF VT Y LK+++DK K

Sbjct 181 NSANDILENMVWQATINFEIDAINIHLPPNSNFNFVVTGYKLKLIEDKSKK 231

P-type conjugative transfer protein VirB9 [Rickettsiaceae bacterium]
Sequence ID: [RYE05781.1](https://www.ncbi.nlm.nih.gov/protein/RYE05781.1?report=genbank&log$=protalign&blast_rank=3&RID=8VTAFGF9014) Length: 248

Query 1356 ILIFYANSSFAIRKSRPTSIDSRIRIMVYNPDNVFKYTGYYGYQASIELLKDEEI*HFY- 1532

I++ + N+S AIR+SRPT IDSRIRIMVYNPD+VFKY GYY YQASIEL K EEI

Sbjct 10 IILLFVNTSQAIRESRPTPIDSRIRIMVYNPDDVFKYVGYYNYQASIELAKSEEIISISM 69

Query 1533 -----WRYYRLANCSLR**NFIKLMEQDATTNMTLITNKLTYFFELYVEETTDMRDPDIL 1697

W+ N FIK ME DATTNMTLITNK TYFFELY EET+D+RDPD+

Sbjct 70 GDTTSWQIVPSGNRI-----FIKPMEHDATTNMTLITNKRTYFFELYAEETSDIRDPDMA 124

Query 1698 FNVRFVYPD--EEEHLRSFSATSSSAPDLTHPEKSNFSYSISSHEEIAPIKVFDDGEFTY 1871

FNVRF+YPD EEEHLR+F A++ + PDL+HPEK NF+YSIS E++APIK+FDDGEFTY

Sbjct 125 FNVRFIYPDEEEEEHLRTF-ASALAGPDLSHPEKYNFNYSISGSEQVAPIKIFDDGEFTY 183

Query 1872 L*FRDKNAELSSNFC--H**RAARVNGQL*IIKRRK*HCYFRASISKIIHKAW*KIICVF 2045

L FRDKNA+L + F R + VN + I K+ ++ KI+CVF

Sbjct 184 LQFRDKNAQLPAIFAVDEDRRESMVNYR--ISTENNTIVIVEQVFQKLAIRSGKKIVCVF 241

Query 2046 NEVFKSY 2066

NE FK Y

Sbjct 242 NESFKPY 248

#### B. C15:3263..4537: murA

UDP-N-acetylglucosamine 1-carboxyvinyltransferase [Rickettsia endosymbiont of Culicoides newsteadi] Sequence ID: [WP_094648791.1](https://www.ncbi.nlm.nih.gov/protein/WP_094648791.1?report=genbank&log$=protalign&blast_rank=1&RID=8VYVG9B8016) Length: 424

Query 4537 MDSIIIKGGKPLIGKVNISGAKNAALPIITAALLAEGNLNLINIPKLTDIYTMKTLLQNH 4358

MDS+IIKGG PL G ++ISGAKN+ALPI+TAALL + L++ NIPKLTDI TMK LL+N

Sbjct 1 MDSLIIKGGIPLEGNISISGAKNSALPIMTAALLTD-KLSINNIPKLTDILTMKKLLENL 59

Query 4357 GILIDIKDLGDYYALSINSNKVDNFVAPYDIVRKMRASIWVLGPLLARFGKAQVSLPGGC 4178

G ++ + D DY ++ I+S+ ++NF APYDIVRKMRASIWVLGPLL+RF KA+VSLPGGC

Sbjct 60 GSVVTVTDHQDYLSMDIDSSNINNFTAPYDIVRKMRASIWVLGPLLSRFSKAKVSLPGGC 119

Query 4177 AIGARQVDLHIDVLRAMGADIEITHGYIKAKSKGRLKGVHFNFNKISVGATINAIMAATL 3998

AIGARQVDLHI L+AMGADI++ HGYI AK GRLK VHF F +ISVGATIN+IMAATL

Sbjct 120 AIGARQVDLHIAALQAMGADIDVDHGYINAKITGRLKAVHFVFKQISVGATINSIMAATL 179

Query 3997 AKGETVLLNCAREPEIIDLCKCLTAMGANIDGDSTGEIKITGVESLKGANYKVMPDRIEA 3818

AKGET+L NCAREPEI+DLC CL MGA I+G T EI I G LK NY +MPDRIEA

Sbjct 180 AKGETILSNCAREPEIVDLCHCLNKMGAEINGIGTSEITIIGKSYLKHTNYSIMPDRIEA 239

Query 3817 GTYMIAAAITKGKLDILGIDYHIIENLGLKLIDAGVEVSPLENGVRLKYSGNIKSVDIQT 3638

GTYM+AAAITKG L+I GI+ +I+EN+ LKLI+AG++V P++NG+R+ Y + VDI T

Sbjct 240 GTYMMAAAITKGSLNIYGINGNIVENIVLKLIEAGIKVEPIDNGLRVSYVDKLNPVDIHT 299

Query 3637 EAYPGFSTDLQAQFMSLMTLAEGAAVITENIF*NRFMHIPELCRMGANITISGHSAMIRG 3458

YPGF+TD QAQFMSLMTL G++ ITE IF NRFMH+PELCRMGANI I G+SA+I G

Sbjct 300 SPYPGFATDFQAQFMSLMTLCSGSSTITETIFENRFMHVPELCRMGANIIIKGNSAIIHG 359

Query 3457 VEHLSGAEVMASDLRASVSLVLAGLAAKGETIIRRVYHLDRGYQQLEQKLSHCGADIKRI 3278

V L+GAEVMASDLRASVSLVLAGLAA T IRR+YHLDRGYQ LE+KLS+CGA+I R+

Sbjct 360 VPFLTGAEVMASDLRASVSLVLAGLAANDTTKIRRIYHLDRGYQTLEKKLSNCGANIVRV 419

Query 3277 LGESI 3263

G+++

Sbjct 420 TGDNV 424

#### C. C15:4762-6303, Acyl-CoA carboxylase subunit beta

acyl-CoA carboxylase subunit beta [Rickettsiaceae bacterium]
Sequence ID: [RYE05809.1](https://www.ncbi.nlm.nih.gov/protein/RYE05809.1?report=genbank&log$=protalign&blast_rank=1&RID=8W05EZAD016) Length: 514

Query 4762 MNQTILPDQDLLEEKRSIARAGGGHDRIALQHKKGKLTARQRVEAFLDPDSFEETGMFVE 4941

MNQ +L QDLLEEKRSIARAGGG +RIALQHKKG+LTAR+R+E LDPDSFEETGMFVE

Sbjct 1 MNQNVLQGQDLLEEKRSIARAGGGLERIALQHKKGRLTARERIEVLLDPDSFEETGMFVE 60

Query 4942 HRCDNFGMKDKKFAGDGVVTGHGTINGRLVFIYSQDFTVLGGSLGEYHAKKICNVIDSAL 5121

HRCDNFGMKDKK AGDGVVTGHGTINGRLVFIYSQDFTVLGGSLGEYHAKKIC+V+DSAL

Sbjct 61 HRCDNFGMKDKKTAGDGVVTGHGTINGRLVFIYSQDFTVLGGSLGEYHAKKICSVVDSAL 120

Query 5122 QVGAPVIGINDSGGARIQEGVDALGGYGELFQRNVIASGVIPQITLIMGPCAGGAVYSPA 5301

Q GAPVIGINDSGGARIQEGVDALGGYGELFQRNV+ASGVIPQI+LIMGPCAGGAVYSPA

Sbjct 121 QAGAPVIGINDSGGARIQEGVDALGGYGELFQRNVLASGVIPQISLIMGPCAGGAVYSPA 180

Query 5302 LTDFIFMVRDSSYMFVTGPDVVKTVTGEEVSQEKLGGARMHTTKSGVADLAFKNDIEALL 5481

LTDFIFMVRDSSYMFVTGP+VVK VTGEEVSQEKLGGAR+HTTKSG+ADL+FKNDIEALL

Sbjct 181 LTDFIFMVRDSSYMFVTGPEVVKAVTGEEVSQEKLGGARIHTTKSGIADLSFKNDIEALL 240

Query 5482 ETRRFFNFLPLSNRAPLPTRYTKDPADRVDMSLNTLVPLAANKSYDMKELIQRIVDEG*F 5661

ETRR FNFLPLSNRAPLP+R TKDPADRVDMSLNTLVP NKSYDMKELI+RIVDEG F

Sbjct 241 ETRRLFNFLPLSNRAPLPSRCTKDPADRVDMSLNTLVPNVPNKSYDMKELIKRIVDEGDF 300

Query 5662 FELQPDFAKNILIGFGYMEGRSIGFVANQPLYLAGCLDINASRKAARFIRFCDAFNIPIV 5841

FEL +FAKNI+IGFGYMEGR +GFVANQPLYLAGCLDIN+SRKAARFIRFCDAFNIPI+

Sbjct 301 FELHAEFAKNIIIGFGYMEGRPVGFVANQPLYLAGCLDINSSRKAARFIRFCDAFNIPII 360

Query 5842 SLVDVPGFLPGTAQEHDGIIKHGAKLLYAYAEATVPKITVITRKAYGGAYIVMNSKHLRG 6021

SLVDVPGFLPGT QE++G+IKHGAKLLYAYAEATVPKITVITRKAYGGAYIVMNSKHLRG

Sbjct 361 SLVDVPGFLPGTNQEYNGLIKHGAKLLYAYAEATVPKITVITRKAYGGAYIVMNSKHLRG 420

Query 6022 DVNYAWVNPEIAVMGAEGAAEIIFKEDCKDSDLKKKKIQEYKDTVTSLFVAASRGYLDDI 6201

DVNYAW+N EIAVMGAEGAAEIIFK +CKD + KK IQEYKDT+TS F+AASRGY+DDI

Sbjct 421 DVNYAWINSEIAVMGAEGAAEIIFKNECKDPEQKKILIQEYKDTITSPFIAASRGYIDDI 480

Query 6202 IKPQNTRWRICKALNFLQGKNTQMPWKKHDNLPL 6303

IKPQNTRWR+CKALNFL+ K Q+PWKKHDNLPL

Sbjct 481 IKPQNTRWRLCKALNFLREKKVQVPWKKHDNLPL 514

#### D. C15: 6336-8333

acetyl/propionyl/methylcrotonyl-CoA carboxylase subunit alpha [Rickettsiaceae bacterium]
Sequence ID: [RYE05810.1](https://www.ncbi.nlm.nih.gov/protein/RYE05810.1?report=genbank&log$=protalign&blast_rank=1&RID=8W20HP85014) Length: 665

Query 6336 MNKPLFDKVLIANRGEISLRIMRSLKKMGIKSVAVYSEADTGSKHVQEADEAYYVGNSPA 6515

M+KPLFDKVLIANRGEI+LRIMRSLKKMGIKSVA+YSEADT S HVQ ADEAYYVGNSPA

Sbjct 1 MSKPLFDKVLIANRGEIALRIMRSLKKMGIKSVAIYSEADTNSMHVQYADEAYYVGNSPA 60

Query 6516 TESYLSIKNIVNAARASGAQAVHPGYGFLAENFNFANILKREGITLIGPSAQAIKQMGDK 6695

TESYLSIKNI+NA RASGAQAVHPGYGFLAEN NFA+ LKREGITLIGPSA AIKQMGDK

Sbjct 61 TESYLSIKNIINAIRASGAQAVHPGYGFLAENANFASALKREGITLIGPSAGAIKQMGDK 120

Query 6696 IEAKKIATEAGVTTVPGYMGIIGNVNQAISIAEEIGFPVIVKAAAGGGGRGMRVVKNSSE 6875

IEAKKIATEAGVTTVPGYMGII N+ QAISIAEEIGFPVIVKAAAGGGGRGMRVVKNS+E

Sbjct 121 IEAKKIATEAGVTTVPGYMGIIRNITQAISIAEEIGFPVIVKAAAGGGGRGMRVVKNSAE 180

Query 6876 MAAAFESAKLEAENSFNDGRVFIEKLIENPRHIEIQLIADQFGNAVCLGERECSIQRHHQ 7055

MA AFESAK+EAENSF+DGRVFIE+LIE+PRHIEIQL+ADQFGNAVCLGERECSIQRHHQ

Sbjct 181 MAQAFESAKIEAENSFDDGRVFIERLIESPRHIEIQLLADQFGNAVCLGERECSIQRHHQ 240

Query 7056 KIIEEAPSSFITEEIRQKMYAEVIALTHKVGYYSAGTVEFIMDPDKHYYFMEMNTRLQVE 7235

KIIEEAPS FITEEIR++MYAEVIAL++KVGYYSAGTVEFIMD K YYFMEMNTRLQVE

Sbjct 241 KIIEEAPSVFITEEIRKQMYAEVIALSNKVGYYSAGTVEFIMDSKKQYYFMEMNTRLQVE 300

Query 7236 HPVTELVTGIDIVEEMIKIAAGEKLSFTQDDIKLKGWAIESRICSEDPTRGFLPSSGRIT 7415

HPVTELVTGIDI+EEMIKIAAGEKLSFTQ+DIK+KGWAIE+RICSEDPTRGFLPSSGRIT

Sbjct 301 HPVTELVTGIDIIEEMIKIAAGEKLSFTQEDIKIKGWAIEARICSEDPTRGFLPSSGRIT 360

Query 7416 GYAEPLKSPHIRIDSGVSIGGEVSMFYDPMIAKLCTYHDTREQAIEIMQTALSSYVIQGI 7595

YAEPLKSP+IR+DSGV GGEVSMFYDPMIAKLCTYHD R QAIE M++ALSSYVIQGI

Sbjct 361 EYAEPLKSPNIRVDSGVGAGGEVSMFYDPMIAKLCTYHDDRAQAIECMRSALSSYVIQGI 420

Query 7596 SHNISFLEAVMSHPRFISGNINTGFIAEEYPAGFSGASLTSEITDVFLATAIYTYITEQK 7775

SHNISFLEAV+SH RFI GNINTGFIAEEYPAGFSGA+LTSEITDVFL+TAI+ YITEQK

Sbjct 421 SHNISFLEAVISHQRFIDGNINTGFIAEEYPAGFSGATLTSEITDVFLSTAIFAYITEQK 480

Query 7776 RAASIEGQMVDQASKIGTRWVVSIDDRLFPVLIKPVPDGYNIRQGYTRIYIRSNWNLGSH 7955

RA+SI GQ++DQ S IGTRWVVSIDD+LFPVLIKPV +GYNIRQGYTRIYIRSNW++GSH

Sbjct 481 RASSISGQIIDQVSNIGTRWVVSIDDKLFPVLIKPVQEGYNIRQGYTRIYIRSNWHIGSH 540

Query 7956 LFSAVVNGRKVNVKIENISTGYRLTHSGITVKTFVRSPLMSELESIMPVKLQLDDLTELT 8135

LFSA +NGRKVNVKIE+I TGY+LTHSGITVK +VRSP MSELES+M K+ ++D ELT

Sbjct 541 LFSATINGRKVNVKIESILTGYKLTHSGITVKAYVRSPRMSELESVMLGKITVNDQAELT 600

Query 8136 APLAGQIIAVKVQEGDEVIVSQEIMILTAMKMENIILAECAGKIAKIFVKDKDQVSAGQV 8315

APLAGQIIAVK++E +EV+V QE+++LTAMKMENIILAE +GKIAKIF+++KDQVSAGQV

Sbjct 601 APLAGQIIAVKIKEQEEVVVGQELIVLTAMKMENIILAERSGKIAKIFIQEKDQVSAGQV 660

Query 8316 LLEFE 8330

L+EFE

Sbjct 661 LMEFE 665

#### E. C12: R629..1594, IS481 family transposase [Rickettsia bellii]

Sequence ID: [WP_045799898.1](https://www.ncbi.nlm.nih.gov/protein/WP_045799898.1?report=genbank&log$=protalign&blast_rank=28&RID=8Y4X001S016) Length: 364

Query 1594 GYSRD-YYRFKELYEAGGAEALY 1529

GYSRD YYRFKELYE GG EALY

Sbjct 32 GYSRDSYYRFKELYETGGEEALY 54

Query 1547 GCRSFIHEVSRKKPIIANRV---VEKAVLDMAIKYPAYGQLQVSNKLKKEGILVSPGRVR 1377

G ++E+SR+KPIIANRV +EKAV+DMAI+YPAYGQL+VSN+LKK GILVSPG VR

Sbjct 48 GGEEALYEISRRKPIIANRVDPSIEKAVMDMAIEYPAYGQLRVSNELKKSGILVSPGGVR 107

Query 1376 SIWLRNDLNSLKKHLIALETK-EQDGIILTEAQLRVLENRKNAKEAQGKIETEHPVILAV 1200

SIWLRNDLN++ K L ALE K QDGI+LTEAQL+VLE R+N KEA G+IET+HP L

Sbjct 108 SIWLRNDLNNISKRLKALEAKMAQDGIVLTEAQLQVLEKRRNEKEAHGEIETQHPGYLGC 167

Query 1199 KICIT*AVLKA*VKSLPKPLLIHIQG*QAQSFMLKRQLAITSADMLNDRVL----T*GIP 1032

+ K + + + I A + + + AIT+ADMLNDRVL T GIP

Sbjct 168 QDTYYVGNFKG-IGKVYSQVFIDSYTRVADAKLYTDKTAITAADMLNDRVLPWYETQGIP 226

Query 1031 YLEYLLIAVQNIRVT*RIMLLNCF*VLKG*STLLLK-HIPLKLTECVSDSIKL*NEFFDI 855

L L + F ++G K + P C + + EFFD

Sbjct 227 ILRILTDRGSEYKGNIEHHAFELFLSIEGIEHTTTKAYSPQTNGMCERFNKTMKQEFFDT 286

Query 854 VMRKKIYTSLKELQQDLDTWLHYYNYE 774

MRKKIYT L +LQ DLD WL Y+N E

Sbjct 287 AMRKKIYTELDDLQLDLDIWLEYFNNE 313

Query 1212 YLSCQDMYYVGSFKGIGKVFTQTFIDTYSRVAGAKLYAEKTASYYFC*YAK**GADLR-- 1039

YL CQD YYVG+FKGIGKV++Q FID+Y+RVA AKLY +KTA AD+

Sbjct 164 YLGCQDTYYVGNFKGIGKVYSQVFIDSYTRVADAKLYTDKTAIT---------AADMLND 214

Query 1038 ----------YTLLRILTDRGTEYKGNLENHAFELFLSIEGIEHTTTKAYSP*TNGMCER 889

+LRILTDRG+EYKGN+E+HAFELFLSIEGIEHTTTKAYSP TNGMCER

Sbjct 215 RVLPWYETQGIPILRILTDRGSEYKGNIEHHAFELFLSIEGIEHTTTKAYSPQTNGMCER 274

Query 888 FHKTIK 871

F+KT+K

Sbjct 275 FNKTMK 280

Query 772 RPHYGKYYYGKTPMQTFQDSKKLALEKNNEILYPEYLSDSQNSADNQI 629

RPH GKY YGKTPMQTFQDSKKLA+EKNNEILY EY SDSQN DNQ+

Sbjct 314 RPHSGKYCYGKTPMQTFQDSKKLAVEKNNEILYLEYSSDSQNLTDNQV 361

#### F. C12:1890-5078, isoleucine tRNA ligase [Rickettsia endosymbiont of Bemisia tabaci]

Sequence ID: [WP_095568341.1](https://www.ncbi.nlm.nih.gov/protein/WP_095568341.1?report=genbank&log$=protalign&blast_rank=9&RID=8Y4X001S016) Length: 1091

Query 5078 MKKQNIYPEIESGADFPLIEKKILAYWKEHNIFQKSIDNRPATGGIDRAANNEFIFYDGP 4899

M YPE+ S ADF IEK+IL +W+++NIFQKSID R ++EFIFYDGP

Sbjct 1 MTNTKYYPEVSSNADFATIEKEILKFWQDNNIFQKSIDIR--------EGDDEFIFYDGP 52

Query 4898 PFANGLPHYGHLLTGFIKDVYARYQATKGKKVERRFGWDCHGLPAEMGAEKELGFSGRLA 4719

PFANGL HYGHLLTGFIKDVYARYQ +GKKVERRFGWDCHGLPAEM +EKELG SGRLA

Sbjct 53 PFANGLTHYGHLLTGFIKDVYARYQTVRGKKVERRFGWDCHGLPAEMQSEKELGISGRLA 112

Query 4718 ITKFGIDKFNEHCRSSVMKYASEWEKYVNRQARWVDFNNSYKTMDLSFMESVLWAFKELY 4539

IT FGI+KFN HCR SVMKYA EWE+YV RQARWVDF NSYKTMD +FMESVLWAFKELY

Sbjct 113 ITNFGIEKFNAHCRDSVMKYADEWEQYVTRQARWVDFKNSYKTMDKNFMESVLWAFKELY 172

Query 4538 KKGLIYESMRVMPYSWACETPLSNFETRLDNSYRERVDKAVTCSFTLTDK-------PAK 4380

KGL+YESMRVMPYSWACETPLSNFETRLDNSYRER DKAVT SF L PAK

Sbjct 173 NKGLLYESMRVMPYSWACETPLSNFETRLDNSYRERADKAVTVSFELCHPENITDVIPAK 232

Query 4379 APAGFK----------EYKMLAWTTTPWTLPSNLALAVGPEVKYIFVPKEENCYIIAKFA 4230

A K EY++LAWTTTPWTLPSNLALAVG ++ Y VPK + CYIIA +

Sbjct 233 AGIQEKVDPPLRGDDIEYRVLAWTTTPWTLPSNLALAVGSDIDYALVPKGDVCYIIAASS 292

Query 4229 SYKYAKELGIEEGVKCQECRGNELDGITYKPVFDYFVDHPNSFRVLCAEFVAEGDGTGIV 4050

KYAKEL ++ + +G+EL G++YKP+FDYF DHPNSF++ +FV EGDGTG+V

Sbjct 293 VSKYAKELELKGDEQFTIIKGSELQGLSYKPLFDYFKDHPNSFKIFAGDFVVEGDGTGVV 352

Query 4049 HLAPGFGEDDQVVCVQAGIELVCPVDNAGKFTSEVYDFVGLNVFEANDNITIKLKEIGAW 3870

H+APGFGEDDQ++C GI+LVCPVDN+GKFT E+ D GL VF+AND I IKLKE G W

Sbjct 353 HMAPGFGEDDQILCESKGIKLVCPVDNSGKFTKEIPDLEGLQVFDANDKIIIKLKEQGNW 412

Query 3869 LKTEQVLHNYPHCWRTDTPLIYKAVSSWYVEVTKFKDRMVELNQEINWIPSNVKDNLFGK 3690

LKTEQ +HNYPHCWRTDTPLIYKAV SWYV+VT+FKDRMVELNQ+INWIP +VKDNLFGK

Sbjct 413 LKTEQYIHNYPHCWRTDTPLIYKAVPSWYVKVTEFKDRMVELNQQINWIPFHVKDNLFGK 472

Query 3689 WLENARDWSISRNRFWGTPIPIWRSNDPKYPRIDVYASVEELEKDFGVKVTDLHRPYIDQ 3510

WLENARDWSISRNRFWGTP+P+W+S+DP+YPRIDVY S+EELE DFGVK+TDLHRP+ID+

Sbjct 473 WLENARDWSISRNRFWGTPLPVWKSDDPQYPRIDVYGSIEELETDFGVKITDLHRPFIDE 532

Query 3509 LTRANPDDPTGKSIMQRVEDVFDCWFESGSMPYGQAHYPFENKEWFEKHFPADFIVEYSA 3330

LTR NP+DPTGKS M+R+EDVFDCWFESGSMPYGQAHYPF+NK+WFE HFPADFIVEYSA

Sbjct 533 LTRTNPNDPTGKSTMRRIEDVFDCWFESGSMPYGQAHYPFKNKQWFEDHFPADFIVEYSA 592

Query 3329 QTRGWFYTLMVLSTALFDRPPFLNCICHGVILDSSSQKLSKKLRNYADPLELFDKYGSDA 3150

QTRGWFYTLMVLSTALFDRPPFLNCICHGVILD++ QKLSK+L NYADPLELFDKYGSDA

Sbjct 593 QTRGWFYTLMVLSTALFDRPPFLNCICHGVILDATGQKLSKRLNNYADPLELFDKYGSDA 652

Query 3149 LRLTMLSSNVVKGQELLIDKEGKMVYETLRIFIKPIWSAYHFFTLYANADGIRGENILAA 2970

LR+TMLSSNVVKGQELLIDK+GKMV++TLR+FIKPIW+AYHFFT+YANAD I+GE

Sbjct 653 LRVTMLSSNVVKGQELLIDKDGKMVFDTLRLFIKPIWNAYHFFTMYANADHIKGE----- 707

Query 2969 VSNLNRNVLDQYILAKLKTAVSGIAQGLDNFNTQIAYSNIANFFEVLNNWYIRRSRHRFW 2790

++ + NVLD YIL+KLK AV I + LDNF+TQIAY ++ FFEVLNNWYIRRSR RFW

Sbjct 708 LNFTSENVLDVYILSKLKIAVEKIKESLDNFDTQIAYHAVSEFFEVLNNWYIRRSRARFW 767

Query 2789 KSEKDSDKRMAYNTLYTCLQMMCQAMSSLAPLIMEDIYLGL------------------- 2667

KSEKD DK+ AYNTLYTCL+ M AMSSL PLI E IYLGL

Sbjct 768 KSEKDLDKQNAYNTLYTCLETMAIAMSSLVPLISEAIYLGLHCHPRESGDPEKSVFLDSR 827

Query 2666 ------TGANNSGGSVHLTNFPDLNEIEVDPNLIKNMDLILDICNCALFIRSQENIRVRQ 2505

N SVHL N+P+L++ E++ L+ MD +LDIC+ +LFIRS EN RVRQ

Sbjct 828 LRGNDIKDVQNDSLSVHLCNYPNLSKFEINSELVDTMDTVLDICSNSLFIRSSENARVRQ 887

Query 2504 PLAQITLIIKEVNNLRVFEDIIKDEINIKSIVYYNNLENFAIRKLSINFQLVGKRLSNKI 2325

PL+ IT+I K + L+ FED+IKDEIN+KS++Y ++LEN+A++KLSINF ++GKRL K+

Sbjct 888 PLSSITIISKNNDKLKDFEDLIKDEINVKSVIYRDDLENYAVKKLSINFPMLGKRLPAKM 947

Query 2324 KNIITASKKGEWQLQGEQLEIAGEVLNSEEFSIVLEPKGDIKGAKALSDNSGLVILDLEI 2145

K+II ASKK EW+ L I E LNS+E+ ++LEP IKGA + + NS L+ILDLE+

Sbjct 948 KDIIAASKKNEWEATSGGLRICNETLNSDEYKLILEPHSHIKGASSFAHNSSLLILDLEL 1007

Query 2144 TKELEEEGVARDLIRFIQQARKDAGFNVSDRIELEIKGTLRIIQISKVYEQLITEQTLSK 1965

T EL +EG+ARD++R IQQARK+A F ++DRI ++I + +I +Y + I EQTL +

Sbjct 1008 TSELIDEGIARDIVRSIQQARKNADFAITDRILIDID----LPKIIDIYGEFIKEQTLGE 1063

Query 1964 FGSINVAAYECSVELMNEKVKLALK 1890

F + +EL N K++L +K

Sbjct 1064 FAKDFTPDHISEIELENHKLQLKIK 1088

#### G. C12:9205-11498 penicillin-binding protein 1A [Caedimonas varicaedens]

Sequence ID: [WP_082168465.1](https://www.ncbi.nlm.nih.gov/protein/WP_082168465.1?report=genbank&log$=protalign&blast_rank=14&RID=8Y4X001S016) Length: 824

Query 9205 ILALFSHYGSGLPDYQHLADYKPPVVTRLYANDGHMFAEYAWEKRIYVPIQSIPKKVIQA 9384

+LALF HYGSGLPDYQHLADYKPPVVTRLYANDGHMFAEYAWEKRIYVP+QSIPK+VIQA

Sbjct 1 MLALFYHYGSGLPDYQHLADYKPPVVTRLYANDGHMFAEYAWEKRIYVPVQSIPKRVIQA 60

Query 9385 QL 9390

L

Sbjct 61 FL 62

Query 9384 AAEDKNLYTHSGIDIPSIISAALTNIGRVAESKRPVGASTITQQVAKNFLLADIAHAVSY 9563

AAEDKN Y HSGIDIPSIISAALTNIGRVAESKRPVGASTITQQVAKNFLLADIAHAVSY

Sbjct 63 AAEDKNFYQHSGIDIPSIISAALTNIGRVAESKRPVGASTITQQVAKNFLLADIAHAVSY 122

Query 9564 ERKIKEAILAFRL*SAYSKDYILEFFMNEISLGASSYGVAAAALNYFNKSLDELTISEVA 9743

ERKIKEAILAFRL SAYSKDYILE F+NEI LG+SSYGVAAAALNYFNKSLDELTISE+A

Sbjct 123 ERKIKEAILAFRLESAYSKDYILELFLNEIYLGSSSYGVAAAALNYFNKSLDELTISEIA 182

Query 9744 YLAGLPKAPSRYHPARYP--AKIRRNYVIRRMLEDGYITMQEAKDAAAEPIILHERNPGD 9917

YLAGLPKAPSRYHP RYP AKIRRNYV+RRM EDGYIT QEAK+AAAEPI+LHERNPG+

Sbjct 183 YLAGLPKAPSRYHPVRYPEAAKIRRNYVVRRMFEDGYITAQEAKEAAAEPIVLHERNPGE 242

Query 9918 VVHGSYFAEEVRRELIEKFGEKALYQEGLVVRTSLDPRLQELAQQSLRQGLINYDRRHGW 10097

VVHGSYFAEEVRREL+EKFGEKALYQEGLVVRTSLDP+LQE+A+QSLRQGLI+YDRRHGW

Sbjct 243 VVHGSYFAEEVRRELMEKFGEKALYQEGLVVRTSLDPQLQEVARQSLRQGLIDYDRRHGW 302

Query 10098 RGAVLRLPLSKEEKILPEKGKESTALWIPKLKTVVEPSGIGEWHMAIVLSVDAQAVTIGF 10277

RGA+L L LSKEEK LPEK KE+ A WIPKLK VVEPSGIG WHM++VL+V +Q VTIGF

Sbjct 303 RGAILHLILSKEEKTLPEKAKEALAPWIPKLKAVVEPSGIGAWHMSVVLAVGSQEVTIGF 362

Query 10278 KDGTWGYITFKELQWARKYINEYALGGTITRPADVLAVGDVVLVEP-LNNQKQFSLCQIP 10454

KDGT G I+ KELQWARKYINEYALG + +P DVL+VGDV+LVEP LN++K+F LCQIP

Sbjct 363 KDGTSGQISLKELQWARKYINEYALGSGVVKPGDVLSVGDVILVEPSLNDKKKFKLCQIP 422

Query 10455 VVSGAMVVMDPHSGRVLALQGGFSFKISQFNRATQAMRQIGSTFKPFAYLAALEKGLTPS 10634

VSGA++VMDPH+GRVLA+ GGFSFKISQFNRATQAMRQIGSTFKPFAYLAALEKGLTPS

Sbjct 423 AVSGAIIVMDPHTGRVLAMHGGFSFKISQFNRATQAMRQIGSTFKPFAYLAALEKGLTPS 482

Query 10635 TLLYDGPFYLDLGYGLGIWKPRNYERDYLGSITLRRAFELSRNLAPVRMIHEYVGMQNVK 10814

TLLYDGPF +DLG GLG+W PRNYERDYLGSITLRRAFELSRNLAPVRM HEYVGM+NVK

Sbjct 483 TLLYDGPFSIDLGPGLGVWSPRNYERDYLGSITLRRAFELSRNLAPVRMTHEYVGMKNVK 542

Query 10815 QVAEKLGLVEHLPLQLATVLDAAESTILKVTTAYAMIANGGKKITPTLLDRVQDRHGKNL 10994

QVAEKLG+V+HLPLQLATVL AAEST+LKVTTAYAMIANGG+KITPTLLDRVQDRHGKNL

Sbjct 543 QVAEKLGVVDHLPLQLATVLGAAESTLLKVTTAYAMIANGGRKITPTLLDRVQDRHGKNL 602

Query 10995 YVNKGRLCEGCGNVYWMNSPPSLVDRRQQVIDPATAYQMISLLQGDVDRGTAKGIKDLNR 11174

YVN+GR CEGCGNVYWMNSPPSLVDRRQQVIDPATAYQMISLLQGDV+RGTAKG++DLNR

Sbjct 603 YVNQGRFCEGCGNVYWMNSPPSLVDRRQQVIDPATAYQMISLLQGDVERGTAKGVRDLNR 662

Query 11175 PLAGKTGTSNDYRDTWFMGFTPDLVVGVFVGFDALRDMGQHENGARVALPIFKAFMAEAL 11354

PLAGKTGTSNDYRDTWF+GFTPDLVVGVFVGFD RD+GQHENGARVALPIFK FMAEAL

Sbjct 663 PLAGKTGTSNDYRDTWFVGFTPDLVVGVFVGFDVPRDLGQHENGARVALPIFKTFMAEAL 722

Query 11355 KNVPAIPFRIPSGIKLIRVDAMTGLRASGSEPNVIFEAFKPGTDENSY 11498

KN PAIPFRIPSGIK +RVDAMTGL A+GSEPNVIFEAFKPGTDENS+

Sbjct 723 KNTPAIPFRIPSGIKFVRVDAMTGLPAAGSEPNVIFEAFKPGTDENSF 770

#### H. C12:8104-8664, ribonuclease E/G [Caedimonas varicaedens]

Sequence ID: [WP_062138834.1](https://www.ncbi.nlm.nih.gov/protein/WP_062138834.1?report=genbank&log$=protalign&blast_rank=27&RID=8Y4X001S016)Length: 766

Query 8664 MAKMMIIDATHSEETRVALIYEKNRLIDFDFESATKLTVKGNIYLAKVARVEPSLQAAFV 8485

M K MIIDATHSEETRVALI EKNRLIDFDFESATKLTVKGN+YLAK+ARVEPSLQAAFV

Sbjct 1 MTKRMIIDATHSEETRVALISEKNRLIDFDFESATKLTVKGNVYLAKIARVEPSLQAAFV 60

Query 8484 DYGGNRHGFLAFSEIHPDYFRIPVSDREALKEEMNKVSAEEKNPLKKQ*FKKKEAQQSFK 8305

DYGGNRHGFLAFSEIHPDYFRIPVSDREALKEEMNK S EE ++ +E Q+SFK

Sbjct 61 DYGGNRHGFLAFSEIHPDYFRIPVSDREALKEEMNKASLEEH--AEEIILPAQEEQKSFK 118

Query 8304 SPQGVT-------EESEESAFEMQIAILGGEAPPNDEDELRPSKRPLLHQMYKIQEVIHK 8146

+ T EESEESAFEM IAILGGEAP ND+DEL KRPLLHQMYKIQEVIHK

Sbjct 119 DEEKATEEGDEKQEESEESAFEMPIAILGGEAPLNDDDELTSPKRPLLHQMYKIQEVIHK 178

Query 8145 NQILFVQVVKEERG 8104

NQIL VQVVKEERG

Sbjct 179 NQILLVQVVKEERG 192

#### I. c12:7486-8016, aminopeptidase P family protein [Rickettsia hoogstraalii]

Sequence ID: [WP_040255827.1](https://www.ncbi.nlm.nih.gov/protein/WP_040255827.1?report=genbank&log$=protalign&blast_rank=30&RID=8Y4X001S016)Length: 579

Query 8016 TSTLAIGAPTLEEIRCYTNVLRGHINLSKIKFPRGI-TGANLDILARQYLWAESSDYPHS 7840

T T+ IG PT E+ + YT VL+GHI L+K KFP+ I TGANLDILARQYLW E DYPH

Sbjct 401 TRTIMIGTPTDEQKKRYTQVLKGHIALAKAKFPKNIVTGANLDILARQYLWQEMLDYPHG 460

Query 7839 TGHGVGSYLSVHEGPQGINLRNNVSLKPGMILSNEPGYYVPGKFGIRIENLMYVKAADHP 7660

TGHGVGS+LSVHEGPQ INLRN LK GMILSNEPG+Y+ GK+GIRIENLMYVK ++

Sbjct 461 TGHGVGSFLSVHEGPQSINLRNKTILKAGMILSNEPGFYISGKYGIRIENLMYVK--ENS 518

Query 7659 NFLQFETLSLVPYARKLIDFNMLDSSELEYLKQYYQKIHDNIYHLLSPLAKDWLKLQI 7486

+L+FETLSLVPYA KLID +L+ E+ Y+K+YY KI IY LLSP A+DWL +I

Sbjct 519 GWLEFETLSLVPYASKLIDTKLLNIDEINYIKEYYNKIRAKIYDLLSPQARDWLNNEI 576

#### J. COQ9 family protein [Rickettsiaceae bacterium]

Sequence ID: [RYE06526.1](https://www.ncbi.nlm.nih.gov/protein/RYE06526.1?report=genbank&log$=protalign&blast_rank=47&RID=8Y4X001S016) Length: 210

Query 5725 MILEIEQKYQAQRAELITALTELLPFYEWNEKIIHEAEEICGFLPEYTYIIFSSGLPEIR 5546

M EI +KYQ QR + + ELLP++ W+ ++I EAE +CGF Y +++F + I

Sbjct 1 MYTEITEKYQKQRIAIAKTIKELLPYFGWDHQLISEAEAVCGFDQGYCWLLFGKEIANII 60

Query 5545 DFHESQTDVLMVKYLQQEEEPLKI--RERISLALKIRIKKCVSKIICKKNRNYFLQPENL 5372

F+E+ D LM+ L +E KI R+ IS ALKIRIK C ++ KKN+ YFL+PEN

Sbjct 61 QFYENWHDQLMIDLLNLQESTTKIQVRQMISNALKIRIKSCDFQVNMKKNQLYFLKPENY 120

Query 5371 LEGARVAWRTCDLIWRYAGDDSTDFNHYSKRSLLTGVYLSSIMFYIKDESKDYIETDNYI 5192

G+++AW+TC+ IW YAGD S DFN+Y+KR LL VY+SSI +Y+ D+S + +TD +I

Sbjct 121 TLGSKLAWQTCNKIWYYAGDRSIDFNYYTKRGLLFPVYVSSINYYLYDQSDQHQKTDQFI 180

Query 5191 DRTLTKIINIASLKKFAKLPSIEDIPILRLF 5099

D+ L KIINIASLK K+P E+IPILRLF

Sbjct 181 DQALAKIINIASLKN--KIPKFENIPILRLF 209

#### K. 30S ribosomal protein S21 [Rickettsia endosymbiont of Culicoides newsteadi]

Sequence ID: [WP_094649364.1](https://www.ncbi.nlm.nih.gov/protein/WP_094649364.1?report=genbank&log$=protalign&blast_rank=89&RID=8Y4X001S016)Length: 66

Query 5867 VILVNVHAGNGEQAIKNLKRKMQRELVFRAMKMSRFYEPPSVKRVRKAQETERRKRKVAR 6046

+ILVNVHAGNGEQAIKNLKRKMQRELVFR+MKMSRFYEPPSVKRVRK QETERRKRKVAR

Sbjct 1 MILVNVHAGNGEQAIKNLKRKMQRELVFRSMKMSRFYEPPSVKRVRKDQETERRKRKVAR 60

Query 6047 KQMMES 6064

KQMME

Sbjct 61 KQMMEG 66

#### L. amino acid ABC transporter ATP-binding protein [Caedimonas varicaedens]

Sequence ID: [WP_062138842.1](https://www.ncbi.nlm.nih.gov/protein/WP_062138842.1?report=genbank&log$=protalign&blast_rank=1&RID=90WG4DXM016) Length: 241

Query 11906 LVKEVLSVIQDLATQHTMTLAIVTH*MSFAKKFSDMIWFLDAGELVETAEPEHFFSKPQT 11727

+VKEVLSVI+DLA QHTMTLAIVTH M+FAK+ SDMIWFLDAGELVE EP+HFF+KPQT

Sbjct 171 MVKEVLSVIKDLANQHTMTLAIVTHEMNFAKEVSDMIWFLDAGELVEITEPKHFFTKPQT 230

Query 11726 IRAQEFLDMVL 11694

+RAQ+FLDMVL

Sbjct 231 VRAQKFLDMVL 241

#### P. Type II secretion system protein D precursor XpsD and type IV leader (prepilin) peptidase [Caedimonas varicaedens]

Sequence ID: [GAO98863.1](https://www.ncbi.nlm.nih.gov/protein/GAO98863.1?report=genbank&log$=protalign&blast_rank=1&RID=9NV5M5US016) Length: 550

Query 1845 MNFSVRGCLIVLILFMLSSCRTWDTENDLALdpstkmtkqqieenLLRPTSTHIRKARPF 1666

MNF GC+ VL+LF LSSC TWD +NDLALDP TKMTK+QI ENL R + +K RPF

Sbjct 1 MNFPTIGCICVLMLFSLSSCSTWDPDNDLALDPVTKMTKKQIAENLSRTPPSIKKKTRPF 60

Query 1665 AKNSSVPLILKPSKN 1621

K S VP+I KN

Sbjct 61 IKKSDVPVIPAVLKN 75

Query 1684 QKSAPFCKK**CSPYPEALKKPITLSTTEEVPLKDVLFKIARQGGVDLSLDPEVRGGVAL 1505

+K+ PF KK P LK P+TLSTTE VPLKDV F+IARQ VDLS+DP V+GGVAL

Sbjct 55 KKTRPFIKKSDVPVIPAVLKNPVTLSTTESVPLKDVFFQIARQANVDLSIDPNVKGGVAL 114

Query 1504 HTTKRPLINIVKELCTLTCL*YKIENNILRIEPDKPY*INYNAQFLSLTRQNQSRISVAT 1325

H T RPLI+IV+ELCTL L YKIENNILRIEPDKPY +NYNAQFLSLTRQNQSRISVAT

Sbjct 115 HATHRPLIDIVRELCTLNRLRYKIENNILRIEPDKPYLLNYNAQFLSLTRQNQSRISVAT 174

Query 1324 DIFTANEGQAHTADNGSNTLLTGETKNDFWAELESNLSTILQDSEPKGGKQEKSAYSLHK 1145

DIFT NEGQ TADNGSNTLLTGETKNDFWAELESNLSTILQ+S+ K Q+KS+YSLHK

Sbjct 175 DIFTTNEGQGSTADNGSNTLLTGETKNDFWAELESNLSTILQNSDAKEVGQDKSSYSLHK 234

Query 1144 QAGIIYIHVTQAQHQQVEHF 1085

QAGII ++ TQAQHQQVEHF

Sbjct 235 QAGIISVYGTQAQHQQVEHF 254

Query 1088 FLKLLRLSTSSqvlieakivevilkDEFQAGINWNLLKGDLVLQSPMGDTITPGSFNKKA 909

FLKLLRLSTSSQVLIEAKIVEVILKDEF+AGINWNLLKGDLVLQSP+GDTITPGSFNK A

Sbjct 254 FLKLLRLSTSSQVLIEAKIVEVILKDEFKAGINWNLLKGDLVLQSPLGDTITPGSFNKNA 313

Query 908 TIVRDVFTFGASGKQLTEIVSLINKFGTVITLSSPRLTAMNNELGVIKVATNYVYFRRN* 729

T VRDVFTFG SGKQLTEIV LINKFGTV TLSSPRLTAMNNE GVIKVATNYVYFR N

Sbjct 314 TPVRDVFTFGGSGKQLTEIVGLINKFGTVRTLSSPRLTAMNNEPGVIKVATNYVYFRINY 373

Query 728 NRDYGYDSVREYEYVSSEVHTVSIGLIMVVHPSINIADGSIVLSLRPTISRVVDEKPDPA 549

NRDYGYDSVRE+EYVSSE+HTV IGLIMVVHPSIN+ADGSIVLSLRPTISRVVDEK DPA

Sbjct 374 NRDYGYDSVREHEYVSSEIHTVPIGLIMVVHPSINLADGSIVLSLRPTISRVVDEKADPA 433

Query 548 VAIVSKETQQSYVPVVRKQEFESLVCMNSGEFIVMGGLME*ISKNNQSGVLYISEVPLFG 369

VAIVSKE QQSYVPVVRKQEFESLVCMNSGEFIVMGGLME ++KNNQSGV Y+SEVPL G

Sbjct 434 VAIVSKEAQQSYVPVVRKQEFESLVCMNSGEFIVMGGLMEEVAKNNQSGVPYLSEVPLLG 493

Query 368 NLAKGKSDDRVVSELVIFLKATIVDNKADPFVPITESTFS 249

NLAK KS+DRVVSELVIFLKATIVDNKAD FVP+TEST +

Sbjct 494 NLAKAKSEDRVVSELVIFLKATIVDNKADTFVPVTESTVA 533

#### Q. prepilin peptidase [Caedimonas varicaedens]

Sequence ID: [WP_062141155.1](https://www.ncbi.nlm.nih.gov/protein/WP_062141155.1?report=genbank&log$=protalign&blast_rank=11&RID=9NV5M5US016)Length: 142

Query 2058 MSYDSllllllVMAWIGIIYYDMRYRIVPDFlvlsllilgilhyslTYGHFLCAVILGGG 2237

M YDSL LLLL++ W GIIYYDMRYRI+PD LVLSLLILG LHYSLTY HFLCA +LG G

Sbjct 2 MLYDSLALLLLLITWGGIIYYDMRYRIIPDGLVLSLLILGCLHYSLTYEHFLCAGVLGLG 61

Query 2238 GALLKISMEKIMNHPALGWGDVKLAGVLGIGMEPEQISLFLIYIGLVGCAWGGP*KNFER 2417

GALLKISMEK++N ALGWGDVKLA LGIGM PEQISLFLI G +GC WG K +

Sbjct 62 GALLKISMEKLLNRLALGWGDVKLAAALGIGMIPEQISLFLICAGFIGCIWGLFYKIILK 121

Query 2418 TSL--NSPTL 2441

SL +P+L

Sbjct 122 ESLFPFAPSL 131

#### R. tRNA (guanosine(37)-N1)-methyltransferase TrmD [Caedimonas varicaedens]

Sequence ID: [WP_062139502.1](https://www.ncbi.nlm.nih.gov/protein/WP_062139502.1?report=genbank&log$=protalign&blast_rank=1&RID=9P3DUV1P01R) Length: 236 204/231 identities (88%)

Query 24322 MTFEMKIFTLYPEHFPGPQQY*LVGKALQEKKWSLETVNIRDFAFDRHKTVDDTACGGGP 24501

MTF +KIFTLYPE FPGP QY LVG+ALQEKKWSLETVNIRDFAFDRHKTVDDTACGGGP

Sbjct 1 MTFAIKIFTLYPEIFPGPLQYSLVGRALQEKKWSLETVNIRDFAFDRHKTVDDTACGGGP 60

Query 24502 GMVMRPDVIDRALKFHYPTFSKSLIYLSPRGIPLTQEYVKKLARRPSLGLLCGRFEGIDQ 24681

GMVMR DVIDRALKFHYP FSKSLIYLSPRGIPLTQEYVKKLA+RPS+GLLCGRFEG+DQ

Sbjct 61 GMVMRADVIDRALKFHYPVFSKSLIYLSPRGIPLTQEYVKKLAQRPSVGLLCGRFEGVDQ 120

Query 24682 RILDAWEFEEVSIGDFILTGGELPAMALIDACVRILPGVIGSSESLEEESFSQGLLEYPQ 24861

R+LDAWEFEEVSIGDFILTGGELPAMALIDACVR+LPGVIGS+ESLEEESFSQGLLEYPQ

Sbjct 121 RVLDAWEFEEVSIGDFILTGGELPAMALIDACVRVLPGVIGSAESLEEESFSQGLLEYPQ 180

Query 24862 YTRPCTWEGREVPEVLLKGYHKHIRCwrkaqaEEITRTRRPDLWEKYCDAR 25014

YTRP WEGREVP++LL+G+H+ IR WR+AQAEEITR RRPDLW+KYCD R

Sbjct 181 YTRPRVWEGREVPDILLQGHHEQIRRWRQAQAEEITRVRRPDLWKKYCDTR 231

#### S. 16S rRNA processing protein RimM [Caedimonas varicaedens]

Sequence ID: [WP_062139500.1](https://www.ncbi.nlm.nih.gov/protein/WP_062139500.1?report=genbank&log$=protalign&blast_rank=6&RID=9P3DUV1P01R) Length: 179, 135/156/175 (77/89 %)

Query 23784 mdssskilmgFIMGAFGIRGGMRFKSYTESIGNLKTYKVLQDQTGHQRFKIIQLLPYKEN 23963

M+SSSKILMGFIMGA GIRGGMRFKSY ES +LK+YKV QDQTG Q+ KIIQ+LPYKEN

Sbjct 1 MNSSSKILMGFIMGACGIRGGMRFKSYGESPKDLKSYKVFQDQTGCQQLKIIQILPYKEN 60

Query 23964 IITLYLEGITTRSQAEALKGVSLYIDRVqlkkpsreefyyydlegLVVQDEHNCKRGHVK 24143

IITLYLEGITTRSQAEAL+G+SLYID QLKK S++EFYY+DLEGL+V++E NC G VK

Sbjct 61 IITLYLEGITTRSQAEALRGMSLYIDHTQLKKLSKDEFYYHDLEGLIVRNEENCVIGQVK 120

Query 24144 AVVNYGADSILSICLLEDLSAEILVPFRKEFVKevnqkekyiildTDYMRALLDL 24308

VV+YGADS+L +CLLED S+EIL+PFRKEFVK VNQ+EKYIILDTDY++A LDL

Sbjct 121 TVVSYGADSMLGVCLLEDPSSEILIPFRKEFVKAVNQQEKYIILDTDYVQAFLDL 175

#### T. 50S ribosomal protein L19 [Caedimonas varicaedens]

Sequence ID: [WP_062139504.1](https://www.ncbi.nlm.nih.gov/protein/WP_062139504.1?report=genbank&log$=protalign&blast_rank=10&RID=9P3DUV1P01R) Length: 141, 105/109/115 (91/94%)

Query 25038 MNILQQFEQEQLAKLAANSLVPQFSAGDTLRIhvkvvegerertqAYEGVCIAIKNAGIN 25217

MNILQQFEQEQ+ KLAAN+ VPQFSAGDTLRIHVKVVEGERERTQAYEG CIA KNAGIN

Sbjct 1 MNILQQFEQEQVTKLAANNPVPQFSAGDTLRIHVKVVEGERERTQAYEGFCIARKNAGIN 60

Query 25218 SSVTVRKLSFGEGVERvfplyspNIRIEVVRHGDvrraklyylreRTGKAARIAK 25382

SSVTVRKLSFGEGVERVFPLYSPNIRIEVVR+GDVRRAKLYYLR RTGKAARI +

Sbjct 61 SSVTVRKLSFGEGVERVFPLYSPNIRIEVVRYGDVRRAKLYYLRARTGKAARITE 115

#### U. transcription-repair coupling factor [Caedimonas varicaedens]

Sequence ID: [WP_082168494.1](https://www.ncbi.nlm.nih.gov/protein/WP_082168494.1?report=genbank&log$=protalign&blast_rank=18&RID=9P3DUV1P01R) Length: 1159, 82/90/105 (78/85 %)

Query 23457 DRYKIPGALPDEVSNLLKILTFKQLCRKAGIEKLDAGKKGCVLSFFNQSFINPLALVSYI 23636

DR+ G LPDEV NLLKIL KQLCRKAGIEKLDAG+KGCVLSF NQSF NPLALV+YI

Sbjct 1056 DRF---GTLPDEVGNLLKILALKQLCRKAGIEKLDAGEKGCVLSFSNQSFSNPLALVAYI 1112

Query 23637 NEHQGIIRLRPDHKLVFLKVWKTPLLKMEGVKKILQELVALATQK 23771

N+HQG IR RPD KLVFLKVWKTP LK+EGVKKILQEL++LA +

Sbjct 1113 NQHQGTIRFRPDQKLVFLKVWKTPSLKVEGVKKILQELISLADSR 1157

#### V. hypothetical protein BGO77_01970 [Caedibacter sp. 37-49]

Sequence ID: [OJX11001.1](https://www.ncbi.nlm.nih.gov/protein/OJX11001.1?report=genbank&log$=protalign&blast_rank=1&RID=9YPUKV4D014) Length: 642

Query 36642 ALSHFKKLEPVVKTPISCSRVGYWLGRTYEAMGKAKEARTSYQKAAQYKGTFYGQKAFSK 36821

A HF +L V +P S S+ YW GR +A +EA YQKAA Y T+YGQ A

Sbjct 334 AFHHFNQLYQKVTSPYSRSKAAYWAGRAAQAKKLPQEAHLWYQKAAYYPATYYGQLALKA 393

Query 36822 LGHTDKEIILETLRFTPAQQAKFESHKAVKLIRLLAKEEIDEHILSFAYVFAKQTASPLE 37001

L + + LE+L F ++ KFE+H+ V+ ++LL K + + +LSF Y++A QT+S E

Sbjct 394 L-NKPQTFRLESLTFPISEVRKFENHEFVRAVKLLHKAGLTDELLSFGYMYA-QTSSSHE 451

Query 37002 RKQILALVHELAPHYGVEIAQVIAPYQSTLYSEAFPRLKPIYLKHMEKVDAALAHAVIRK 37181

+L+L +AP + V +A ++ +T Y EA+P++ ++ A+ HA+IRK

Sbjct 452 ALMMLSLASTIAPQFAVGMADSMSILHNTHYKEAYPQVCRDNNSAQACLEDAMIHALIRK 511

Query 37182 ESKFNPKIVSWAGAQGLMQLMPETAQLMADQCGVTCSEKQLLTDPLLNVKLGTLYLKEQL 37361

ESKFNP+ S AGA G+MQ++P TA+++A + GV +E +L +D N+ +GT YLKE+L

Sbjct 512 ESKFNPRAKSEAGACGMMQVVPSTAKMVAQKMGVHFNEARLKSDMNYNMLIGTAYLKERL 571

Query 37362 EKYDHSFPLTLASYNAGPGTVSRWLDRFPDPRHPSINTIDWIEVLPYSETRDYIHRVLEN 37541

+ Y S LTLA+YNAGPG+V +W++R+ DPR P ++TIDW+E +PYSETR+YI RV+EN

Sbjct 572 QDYQGSIVLTLAAYNAGPGSVKKWIERYGDPRDPKVDTIDWVEKIPYSETRNYIQRVMEN 631

Query 37542 YTIYKAIL 37565

Y IY+AI

Sbjct 632 YIIYQAIF 639

#### W. DNA translocase FtsK [Rickettsia endosymbiont of Culicoides newsteadi]

Sequence ID: [WP_094649409.1](https://www.ncbi.nlm.nih.gov/protein/WP_094649409.1?report=genbank&log$=protalign&blast_rank=2&RID=A0MDC61S01R) Length: 739 475/576/745 (64/77 %)

Query 3381 mtdyirKIFAYTITQILLYTSLGLICSISLITHSPDDPSFNLVTQKDSSNTLGHFGSYLA 3560

M YI +I + L +G ++ L+T++PDDPSFN VT K SN LG+FG YLA

Sbjct 1 MLYYINRILTNNRVRSALLMLVGCFTTLLLVTYNPDDPSFNSVTAKYPSNLLGYFGCYLA 60

Query 3561 DFLYQLFGLASYIFPLCFFSFAFILFKRGTLSLFVLKAIGMILASIALSVTLAKLSFKSL 3740

D YQ FG+AS+I P+C F ++ ++ R ++ + M+L+ I+LS+ K+ L

Sbjct 61 DIFYQFFGIASFILPICCFFWSLSVW-RLEKKWVTIRIMVMLLSIISLSIVCTKIKIDYL 119

Query 3741 PAGGGGSFGLIIYPLVSQFGPLANIACGIFALIMIFLITGVTYENYLNFLQKVRGLLNFK 3920

PAG GG+ G IIYPLV Q A+ A I I++FL+ + + + N + K+ L+ +K

Sbjct 120 PAGAGGTVGTIIYPLVKQLDARADYALIIVTFILLFLLAEIKFSAFWNAIIKLLKLIPYK 179

Query 3921 --LPTPKLsqafinnpptivpqrttplvnseevfkipeiqsdlkvtsaaVTKSQAFKPVQ 4094

+ P +S + P ++ + T VN EE ++ DL + + +

Sbjct 180 KIISLPTISTNSNSTKPVLIKRAT--FVNQEEASYADKM--DLGKNNDPQSHYLKKQSAS 235

Query 4095 NTSNNEVASLPPIDLLKMPNNQHIKAESVAELKTNAENLISVLTDFGVKGQIVDVSQGPV 4274

N+++ LPPI+LLK ++Q+IK E+++EL+ NA+ L++VL DFGVKGQ+++++QGPV

Sbjct 236 RKENDQLPDLPPIELLKQADSQNIKVETLSELQHNAQILLTVLNDFGVKGQVININQGPV 295

Query 4275 VTLYELEPAPGTKSSRVVGLSDDIARSLSAFSTRIAVVPGRNALGIELPNKQRAFFCLRE 4454

VTLYE EPA GTKSSR++GLSDDIARSLSA STRIAV+PGRN LGIELPNKQRAFFCLRE

Sbjct 296 VTLYEFEPAAGTKSSRIIGLSDDIARSLSAISTRIAVIPGRNVLGIELPNKQRAFFCLRE 355

Query 4455 LIETPEYQDPNILLPLILGKDLAGKPYVADLAKMPHLLVAGTTGSGKSVAINAMIMSLLY 4634

LIETPEYQD +I+LPLILGKDLAGKP+VADLAKMPHLLVAGTTGSGKSVAINAMI+SLLY

Sbjct 356 LIETPEYQDNSIMLPLILGKDLAGKPFVADLAKMPHLLVAGTTGSGKSVAINAMIISLLY 415

Query 4635 RYTPAECRLIMIDPKMLELLVYDNiphlltpvVTGSGKAVVALKWAVREMENRYRLMSNV 4814

RYTP ECRLIMIDPKMLEL YD IPHLLTPVVT KAVVALKWAV+EMENRYR MSN+

Sbjct 416 RYTPEECRLIMIDPKMLELSTYDGIPHLLTPVVTEPNKAVVALKWAVKEMENRYRAMSNI 475

Query 4815 GVRNIAGYNAKIAESLKEGKSLECVVQTGFDPDTGKPIYKSIPIAMKKLPFIVVIVDEMA 4994

GVRNIAGYNAKI E+ GK LE +QTGFD +TGKPIY+ I + M+KLPFIVVIVDEMA

Sbjct 476 GVRNIAGYNAKILEAASSGKILERSIQTGFDSETGKPIYEKIEMNMEKLPFIVVIVDEMA 535

Query 4995 DLMIVAGKDIESSIQRLAQMARAAGIHIIMATQRPSVDVITGVIKANFPSRISFKvtski 5174

DLM+VAGKDIE SIQRLAQMARAAGIHIIMATQRPSVDVITGVIKANFPSRISFKVTSKI

Sbjct 536 DLMLVAGKDIELSIQRLAQMARAAGIHIIMATQRPSVDVITGVIKANFPSRISFKVTSKI 595

Query 5175 dsrtilgeqgseqlLGMGDMLYMGNSSRIIRVHGPFVDDKEVEKVTNYLSNTGTPDYVSA 5354

DSRTILGEQGSEQLLGMGDMLYMGNSS+I RVHGPFVDDKEVEKVT YL TGTP+Y+SA

Sbjct 596 DSRTILGEQGSEQLLGMGDMLYMGNSSKITRVHGPFVDDKEVEKVTEYLRATGTPEYLSA 655

Query 5355 VMESTDDDSINMEDFRDGDD---DETIYKKAKQIVKIERKVSISYIQRCLRIGYNRAANV 5525

V + DDD +N+E GDD DET+YK+A QIV++ERKVSISYIQRCLRIGYNRAA +

Sbjct 656 VTQQLDDDEVNVES--SGDDSTSDETLYKRAVQIVQLERKVSISYIQRCLRIGYNRAATL 713

Query 5526 IDRMERDGIISHPSHTGKREILIEE 5600

+++ME++G+IS P+HTGKREIL+ E

Sbjct 714 VEKMEQNGVISPPNHTGKREILLPE 738

#### X. NADH-quinone oxidoreductase subunit NuoF [Rickettsia rhipicephali]

Sequence ID: [WP_014408207.1](https://www.ncbi.nlm.nih.gov/protein/WP_014408207.1?report=genbank&log$=protalign&blast_rank=2&RID=A0JHK25Z01R) Length: 421 357/390/422 (85/92%)

Query 8232 MLQAQDKIFTNLYGEQGFDLVSSKARGDWDKTKDFIVKGRDWIIEEVKQSGLRGRGGAGF 8053

ML+ +DKIFTNL+G+Q DL SSK RGDW+ TK + KGRD+I+EEVK+SGLRGRGGAGF

Sbjct 1 MLKEEDKIFTNLHGQQSHDLKSSKKRGDWENTKALLDKGRDFIVEEVKKSGLRGRGGAGF 60

Query 8052 STGMKWSFMPKSSTKPSYLVVNADESEPGTCKDRDILRFEPHKLIEGCLLASHAVGAHVC 7873

STGMKWSFMPK+S KP YLVVNADESEPGTCKDRDILRFEPHKLIEGCLLAS A+GA+ C

Sbjct 61 STGMKWSFMPKNSEKPCYLVVNADESEPGTCKDRDILRFEPHKLIEGCLLASFAIGANNC 120

Query 7872 YIYIRGEFYNEASNIQIAIDESYGAGLIGKNACGSGYDLDIYLHRGAGAYICGEETALLE 7693

YIYIRGEFYNEASNIQ A+DE+Y GLIGKNACGSG+D +IYLHRGAGAYICGEETALLE

Sbjct 121 YIYIRGEFYNEASNIQRALDEAYKEGLIGKNACGSGFDCNIYLHRGAGAYICGEETALLE 180

Query 7692 SLEGKKGMPRLKPPFPAGTGLYGCPTTINNVESIAVVPTILRRGTSWFAGIGKPNNTGTK 7513

SLEGKKGMPRLKPPFPAG GLYGCPTTINNVESIAVVPTILRRG SWFAGIGKPNNTGTK

Sbjct 181 SLEGKKGMPRLKPPFPAGFGLYGCPTTINNVESIAVVPTILRRGASWFAGIGKPNNTGTK 240

Query 7512 LYCISGHVNQPCNIEEAMGIPLKELIEKYAGGVRGGWNNLKAIIPGGSSVPMIPKEMCET 7333

++CISGHVN+PCN+EEAMGI LKELIEKYAGGVRGGW+NLKAIIPGGSSVP++PK +CE

Sbjct 241 IFCISGHVNKPCNVEEAMGISLKELIEKYAGGVRGGWDNLKAIIPGGSSVPLLPKLLCE- 299

Query 7332 VTMDFDTLRALGSGLGTGGIIVMDKSTDVIYAIARLSKFYMYESCGQCTPCREGTGWMWR 7153

V MDFD+LR GSGLGTGGIIVMDKSTD+IYAIARLSKFYM+ESCGQCTPCREGTGWMWR

Sbjct 300 VEMDFDSLRTAGSGLGTGGIIVMDKSTDIIYAIARLSKFYMHESCGQCTPCREGTGWMWR 359

Query 7152 VMMRLVKGQAKMEEIDQLLDVTKQVEGHTICALGDAAAWPIQGLIHHFRHEIEDRIKAYS 6973

VMMRLVKG AK EID+LL+VTK++EGHTICALGDAAAWPIQGLI HFR EIE RIK+YS

Sbjct 360 VMMRLVKGNAKKSEIDELLNVTKEIEGHTICALGDAAAWPIQGLIRHFRSEIEARIKSYS 419

Query 6972 PI 6967

+

Sbjct 420 VV 421
