# Supplementary material for: Rickettsial DNA and a trans-splicing rRNA group I intron in the unorthodox mitogenome of the fern Haplopteris ensiformis
Source: Commun Biol. 2023 Mar 20;6:296. doi: 10.1038/s42003-023-04659-8 (PMC10027690; doi:10.1038/s42003-023-04659-8)
Supplement: Supplementary file 8 — Supplementary Data 5 [file 42003_2023_4659_MOESM8_ESM.docx]

### Supplementary data 5. Bacterial nucleotide sequence similarities^[[1]](#footnote-1)^ in the *Haplopteris ensiformis* mitogenome.

Alignments exemplarily displaying bacterial nucleotide sequence similarities in the *Haplopteris ensiformis* mitogenome.

#### A1 x1850 matches *serS-surE-nlpD* region, Caedibacter varicaedens WGS sequence

Sequence ID: [BBVC01000020.1](https://www.ncbi.nlm.nih.gov/nuccore/BBVC01000020) Length: 82203, 1496/1850, 81%

Query 1 GAAGGCCAAACCGATGCAGACAATATGGAAGTTCGAAGGTGGGGTTCACCACGTACTTTT 60

||||| | ||| ||| |||| || || ||| |||||| |||||| | |||||||| |||

Sbjct 25296 GAAGGACGAACAGATTCAGAGAACATAGAAATTCGAAAGTGGGGAACGCCACGTACGTTT 25237

Query 61 GATTTTACACCTTTACCCCATTATGAAATCGGTGAAAAATTACAAGAAATGGATTTTGAG 120

||||| ||||| |||||||||||||||||||||||||||||||| | |||||||||||

Sbjct 25236 GATTTCACACCCTTACCCCATTATGAAATCGGTGAAAAATTACAGGGCATGGATTTTGAT 25177

Query 121 AATGCCGCTAAAATTGCCGGCGCACGTTTTTTAATCCTCAAAGGAAATTTAGCGCGACTT 180

| ||| | |||||| |||| || |||||| | ||||||||||| |||||| || |||

Sbjct 25176 ACCGCCACAAAAATTTCCGGTGCTCGTTTTGTTGTCCTCAAAGGAGCTTTAGCTCGGCTT 25117

Query 181 GAACGGGCTTTAGGACAGTTTATGCTTGATTTACACACTCAATAATTTGGCTATCAAGAG 240

|| || ||| | |||||||||||||| ||| | || || || ||||||||||||| ||

Sbjct 25116 GAGCGCGCTCTGGGACAGTTTATGCTAGATCTGCATACCCAGGAATTTGGCTATCAGGAA 25057

Query 241 GTTTCTCCTCCGCTACTTGTTCGAGATGAAACGGTTTATGGCGTAGGACAATTACCTAAA 300

||||| ||||| || || || || ||||| | ||||||||||||||||| | || |||

Sbjct 25056 GTTTCCCCTCCACTGCTCGTGCGGGATGACGCTGTTTATGGCGTAGGACAGCTGCCCAAA 24997

Query 301 TTTAAAGAAAACCTTTTTCAAACTACCGATGGACGATGGCTTATTTCTACAGCAGAAGTT 360

|| | |||| | || || || || | || ||||||||||||||||||||||||||||||

Sbjct 24996 TTCAGAGAAGATCTGTTCCAGACCTCGGACGGACGATGGCTTATTTCTACAGCAGAAGTT 24937

Query 361 TCACTCACAAATCTTGTACGAGAAAAGATTGTAGATGAAGGAGTACTCCCTCTCCGTTTT 420

||||| || ||||| ||||| |||| || | ||||| ||| |||||||| || ||

Sbjct 24936 TCACTGACTAATCTGGTACGGGAAAGAATCCTGGATGAGGGAAGCCTCCCTCTTCGCTTC 24877

Query 421 ACAGCTCTTACCCCCTGTTTTCGTTCTGAAGCCGGCTCAGCAGGACGAGATACGCGCGGC 480

|||||| |||| ||||||||||| || ||||| || |||| || || ||||| || |||

Sbjct 24876 ACAGCTTTTACACCCTGTTTTCGGTCAGAAGCGGGTGCAGCCGGTCGGGATACTCGAGGC 24817

Query 481 ATGATTCGCCAGCATCAATTTCATAAAGTTGAACTGGTAAGCATCGTACATCCCGACGCC 540

||||| |||||||| ||||| |||||||||||||| || || ||||| |||||||| ||

Sbjct 24816 ATGATCCGCCAGCACCAATTCCATAAAGTTGAACTTGTCAGTATCGTTCATCCCGATGCT 24757

Query 541 GCTGAGGAAGAACATCAACGTATGGTAAATGCTGCGGAAACAGTCTTACAACTTCTTAAG 600

|| ||| ||||||||| || ||||| || || || ||||| || ||||||| || |

Sbjct 24756 GCAGAGACAGAACATCAGCGCATGGTGAACGCCGCAGAAACGGTTTTACAACGCCTGGAA 24697

Query 601 ATTCCTTATCGAATCATGTTACTATCATCGGGAGATACGGGTACTCAGTCTCGCCGAACT 660

||||| ||||| | ||||| | |||| || |||||| || ||| ||||||||

Sbjct 24696 ATTCCCTATCGCGTTATGTTGTTGTCATGTGGTGATACGAGTGGCGCGTCACGCCGAACC 24637

Query 661 TATGACCTTGACGTCTGGCTACCTGGATAAAATTTATATCGCGAGATCTCAAGCTGTTCT 720

||||| ||||| |||||| |||| ||| ||||| | ||||| ||||| |||||||||||

Sbjct 24636 TATGATCTTGAAGTCTGGTTACCGGGAGAAAATATGTATCGAGAGATTTCAAGCTGTTCA 24577

Query 721 AACTGTGGGGATTATCAAGCGCGTCGAATGCAAGCGCGATTTCGGCCTAAGAATGGTGCA 780

|| ||||| |||||||| || ||||| ||||| || || ||||| || || |||| ||

Sbjct 24576 AATTGTGGTGATTATCAGGCACGTCGGATGCAGGCACGCTTTCGCCCCAAAAATGACACA 24517

Query 781 GGAAATAAGAACTCACCCGAGTTTGTTTTTACTATCAATGGCTCGGGGGTAGCAGTAGGG 840

|| | ||| || | | |||||||| ||||| ||||||| || || || || ||||||

Sbjct 24516 GGGAGTAAAAATACTACTGAGTTTGTGTTTACCCTCAATGGATCAGGCGTTGCTGTAGGG 24457

Query 841 CGCGCACTTATTGCGGTCATAGAGAATTCTCAAAATAGCGACGGTTCAATAACCATTCCG 900

|| || || ||||| ||||| || || | |||||| || ||||||||||| || |||||

Sbjct 24456 CGTGCGCTCATTGCTGTCATTGAAAACTATCAAAACAGTGACGGTTCAATGACAATTCCA 24397

***> serS ****

Query 901 GATATTTTAAGACCTTATATGAAAGGCATGGAAAGGATTTCACTTCATGATTAAGATAAT 960

|| || ||||||||||||||||||||| |||||||||| ||||||||||| |||||

Sbjct 24396 GAAGCCTTGAGACCTTATATGAAAGGCATGAAAAGGATTTCTATTCATGATTAAAATAAT 24337

***> surE***

Query 961 GGATCAAAAGCCTCGCATTCTTATCTCTAATGATGACGGTATTCATGCCCCGGGTCTTAA 1020

| |||||||||| ||||||||||||||||||||||||||||| |||||| ||||||||||

Sbjct 24336 GCATCAAAAGCCGCGCATTCTTATCTCTAATGATGACGGTATCCATGCCGCGGGTCTTAA 24277

Query 1021 GGTTTTAGAAGATATTGCATCTACCATTACTGATGACATCTGGATTGTGGCACCCGAATG 1080

|| | |||||||||||||| | || || ||||||||||||||||| || || |||||

Sbjct 24276 AGTCCTCGAAGATATTGCATCCTCAATGACCGATGACATCTGGATTGTCGCGCCTGAATG 24217

Query 1081 GGAACAAAGTGGGGCGGCTCATTTTCTGTCGATCGGTCGCCCTCTTCGTTTCCGTGAATT 1140

||||||||| || |||||||||| ||| || |||||||| ||||||||||| || |||||

Sbjct 24216 GGAACAAAGCGGCGCGGCTCATTCTCTCTCTATCGGTCGTCCTCTTCGTTTTCGCGAATT 24157

Query 1141 GGGGCCGAAACGCTACACTGTCAACGGTACTCCTACAGATTGCGTCATGATTGCTGTTAA 1200

||| || || ||||| || |||||||| |||||||| ||||| || ||||| || || ||

Sbjct 24156 GGGTCCAAAGCGCTATACAGTCAACGGAACTCCTACGGATTGTGTAATGATCGCCGTCAA 24097

Query 1201 TAAACTTATGACGGCATGCCGTCCTGATTTGATGTTGTCAGGAGTTAACCATGGAGCTAA 1260

||||| ||||| | ||||| |||||||||||||| || || || || |||||||||||

Sbjct 24096 CAAACTGATGACCGAGTGCCGCCCTGATTTGATGTTATCCGGCGTCAATCATGGAGCTAA 24037

Query 1261 TTTAGGCGAAGATGTGACTTACTCGGGCACAGTAGCCGCCGCTATGGAAGCCACCCTTCT 1320

|||||| ||||| || |||||||| || ||||| || |||||||||||||| ||||||||

Sbjct 24036 TTTAGGAGAAGACGTTACTTACTCTGGGACAGTTGCAGCCGCTATGGAAGCAACCCTTCT 23977

Query 1321 TGGTATTCCCGCTATTGCCCTCAGTCAATCGCTCATTACAGACCATGAAAATGGTTATGA 1380

||||||||| ||||||||||||||||||||||| | || || |||||||||| |||||

Sbjct 23976 TGGTATTCCTGCTATTGCCCTCAGTCAATCGCTTTTGACTGATCATGAAAATGCCTATGA 23917

Query 1381 TCCTGCGCGTCATTATGGGCCTATTCTGATCAAAAAACTTATCTCTCAGACATGGCCGCG 1440

||| || ||||| ||||| || |||| ||||||| || || || || |||||||| ||

Sbjct 23916 TCCCGCTCGTCACTATGGCCCCCTTCTCGTCAAAAAGCTCATTTCCCAAACATGGCCTCG 23857

Query 1441 TCATGTCTTGATTAATATCAATTTCCCCGATATCAGTGTCGATAAAATTCAGGGAGCACG 1500

||||||| |||| ||||| ||||| || ||||||||||||||||||||||||||||| ||

Sbjct 23856 TCATGTCCTGATCAATATTAATTTTCCTGATATCAGTGTCGATAAAATTCAGGGAGCTCG 23797

Query 1501 CGTTGTGCACCAAGGATTGCGTAACATTAATGATAACCTTGTTAAATGGAAAGATCCCCA 1560

||||| | |||||||| || || ||||||||||| || || |||||||| || || ||

Sbjct 23796 TGTTGTACGTCAAGGATTACGCAATATTAATGATAATCTCGTCAAATGGAAGGACCCTCA 23737

Query 1561 TGGCAAGCCCTTTTTCTGGATTGGTGGAAATCGTGATGATTCACCTACAGAAGAGGAAAC 1620

|| || |||||||||||||||||||||||||| |||||||| ||||||||||| |||||

Sbjct 23736 CGGAAAACCCTTTTTCTGGATTGGTGGAAATCGCGATGATTCTCCTACAGAAGAAGAAAC 23677

Query 1621 AGACTTAGAAGCTATTCATCAAGGATCTATTTCTATTACACCTCTTCACCTGGATTTAAC 1680

|| || |||||||| || || || ||||||| ||||||||||||||| | || || ||

Sbjct 23676 CGATTTGGAAGCTATCCACCAGGGCGCTATTTCCATTACACCTCTTCACTTAGACTTGAC 23617

rA5**|**>rA6 (cp375)

***|*** rL1 **R295 *> surE ****

Query 1681 CCATCATTCAACTTTAGAAACCCTTAAACTTGCTTTTATATAATCTTATGAAAACTATTC 1740

||||||||||||||||||||||||||||||||||||||||| ||| ||||||||||||

Sbjct 23616 CCATCATTCAACTTTAGAAACCCTTAAACTTGCTTTTATATGATC--ATGAAAACTATTT 23559

***> nlpD***

Query 1741 CCGAACTCCTTGCTATCGTTATTCTTCTTACTTCCTGCGCTGAAAGAACTGCACCTCCCG 1800

|| || ||| || | | |||| || || |||| ||||||| | ||||| |

Sbjct 23558 CCTGTCTTCTTTCTGTTGCGTTTCTACTGACAGCCTGTTGGCAAAGAACGTCCCCTCCTG 23499

Query 1801 CACCTGTGTCATATGGTTACTATGAACCCTCAGCCCCAGCAACATCTCAA 1850

| |||||||| ||||||||| |||| || ||||| || || ||||||||

Sbjct 23498 CTCCTGTGTCTTATGGTTACCATGAGCCATCAGCAGCAACATCATCTCAA 23449

#### B1. chr.2 195,248..200,299 including x1993-x1442-x1246 *murA*-CoA-carboxylase subunits Rickettsiaceae bacterium isolate PMG_002 WGS sequence

Sequence ID: [SEEW01000001.1](https://www.ncbi.nlm.nih.gov/nuccore/SEEW01000001) Length: 1121132, 3776/5073, 74 %

Query 7 ATATCAGCGCCGCAGTGAGATAATTTTTGCTCAAGTTGTTGATATCCACGATCTAAATGA 66

||||| || ||||| ||||||||| || |||| |||||||| ||||| ||||||

Sbjct 206290 ATATCTGCTCCGCAAGAAGATAATTTATGTTCAAAAGCCTGATATCCTCGATCCAAATGA 206349

Query 67 TATACCCTTCGTATTATTGTTTCTCCTTTAGCGGCAAGGCCTGCCAAAACGAGAGAAACT 126

|| || |||| |||| |||| || || | || ||||| |||| || | || || |

Sbjct 206350 TAAACTCTTCCTATTCTTGTCTCACCCGTTGCAGCAAGTGCTGCTAATATTAGGCAACTT 206409

Query 127 GAAGCTCTAAGATCACTTGCCATGACTTCTGCCCCGCTTAAATGTTCAACTCCTCTAATC 186

||||| || | ||||| ||||| ||||| || || |||| | || |||||| | ||

Sbjct 206410 GAAGCCCTTAAGTCACTAGCCATTACTTCAGCGCCTTTTAAGTATTTTACTCCTTTGATT 206469

Query 187 ATTGCACTATGTCCACTAATAGTTATATTAGCACCCATTCGGCAGAGTTCAGGAATATGC 246

|| ||| | ||| | | ||| | ||||| || ||||| || ||||| || | |||

Sbjct 206470 ATAGCATTGTGTTCTTTTATAATGATATTTGCTCCCATACGATTTAGTTCTGGCACGTGC 206529

Query 247 ATAAAGCGATTTTAAAAGATATTTTCTGTAATAACAGCGGCTCCCTCAGCAAGAGTCATC 306

||| ||||||||| ||| || |||||||| || || | ||||| || |||| ||| |

Sbjct 206530 ATATAGCGATTTTCAAATATGTTTTCTGTGATTACCGATGCTCCATCTGCAATAGTGAGT 206589

Query 307 AGGCTCATAAACTGCGCTTGGAGATCAGTAGAAAAACCAGGATATGCTTCAGTTTGAATA 366

|| |||||||| || ||||| | |||||| | ||| || || || ||||||| | ||||

Sbjct 206590 AGACTCATAAATTGAGCTTGTAAATCAGTTGCAAATCCCGGGTACTCTTCAGTATTAATA 206649

Query 367 TCTACCGACTTTATATTACCGCTATACTTTAATCTTAC-TCCATTCTCTAAA---GGGC- 421

|| | || || ||| | | | | || || | |||| || || | ||

Sbjct 206650 TCAGTAGGTTTGATGA---CGCCAGAGTGT---CTGACATTTATTC-CTGAATTTGTGCG 206702

Query 422 --TAACTTCGACTCCAGCATCTA-TAAGCTTTAA-TCCTAAATTTTCAATAATATGGTAA 477

| ||| ||||| || | ||| ||||| | ||| |||||| | || | |||

Sbjct 206703 TGTTACTATTGTTCCAGACTCAAGTAA--TTTAAGTACTAGATTTTCTACTATTTTATAA 206760

Query 478 TCAATTCCAAGAATATCTAACTTTCCTTTAGTAATAGCAGCAGCAATCATGTAGGTCCCT 537

|| ||||||| ||||| | | ||||| ||||| |||||||||||||||||||| ||

Sbjct 206761 TCTATTCCAACTATATCAAGTTCACCTTTTGTAATTGCAGCAGCAATCATGTAGGTGCCA 206820

Query 538 GCTTCGATTCTATCAGGCATAACTTTATAATTTGCACCTTTTAAAGATTCAACGCCGGTA 597

||||| ||||| || ||||| || | ||| | | ||||||||||| ||| || |

Sbjct 206821 GCTTCTATTCTGTCTGGCATTACATCGTAAGTGACTCCTTTTAAAGAACTAACTCCCATG 206880

Query 598 ATTTTAATTTCTCCTGTGGAATCACCATCTATATTTGCTCCCATTGCAGTCAAACATTTA 657

||||| |||||| | || | || | || || ||||||||| | ||| | |||

Sbjct 206881 ATTTTTATTTCTGCAGTACCTGCTCCTTTGATTTTAGCTCCCATTTTACATAAATACTTA 206940

Query 658 CAAAGGTCAATAATTTCGGGTTCCCGCGCGCAATTAAGCAAAACAGTTTCTCCTTTTGCT 717

|| | || | || || ||||| ||||| || || |||||| |||| |||

Sbjct 206941 CACAAATCTACGATCTCTGGTTCTTTTGCGCAGTTTGTTAATGAAGTTTCACCTTCAGCT 207000

Query 718 AAAGTTGCAGCCATAATGGCATTGATAGTGGCACCAACGGAAATTTTATTAAAATTAAAG 777

|| | ||||||| || ||| ||||||| || || || |||||| ||| |||||||||

Sbjct 207001 AATGAAGCAGCCAATATCGCAGTGATAGTTGCTCCTACCGAAATTCTATGAAAATTAAAA 207060

Query 778 TGAACGCCCTTTAATCTACCTTTGCTTTTTGCTTTTATATAACCATGTGTAATTTCAATG 837

|| ||||||||| ||||| ||| | ||||| ||||| || ||| |||||||||

Sbjct 207061 TGGGTACCCTTTAATTGTCCTTTACTTGTAGCTTTAATATATCCTTGTTCTATTTCAATG 207120

Query 838 TCAGCTCCCATCGCTCTTAAGACATCTATATGCAAATCGACTTGTCTTGCTCCTATTGCG 897

| ||| ||| ||| ||| ||| || |||| ||| ||||||| || || || |||

Sbjct 207121 TGAGCATTCATGGCTTCTAAAACACTGATGTGCATATCAACTTGTCGAGCGCCAATAGCG 207180

Query 898 CATCCCCCTGGAAGGGAGACTTGCGCTTTCCCAAATCTTGCAAGCAGTGGTCCTAAAACC 957

||||| || || | || | || || | ||||||||||| || || || || | |||

Sbjct 207181 CATCCTCCAGGTAATGATATTTTAGCCTCACCAAATCTTGCTAGTAGCGGGCCAAGTACC 207240

Query 958 CAGATGGAGGCTCGCATTTTTCTGACAATATCATAAGGAGCTACAAAATTATCAACCTTA 1017

|| || || || |||||||| | ||||| ||||| || || | ||||| || ||

Sbjct 207241 CATATTGAAGCACGCATTTTACGTACAATCTCATATGGGGCAATAAAATCTGTAATTTTG 207300

Query 1018 TTACTATTAATCGAGAGTGCA----TAATAAT-CTCCTAAATCTT---TAATATCTATTA 1069

||| || ||| ||| | || | || || || ||| | || || ||||

Sbjct 207301 CTACAATCAAT-----GTGTAACTCTAGTGATTCTGCTTGATCATAGCTACTAACTATAG 207355

Query 1070 AAATTCCGTGATTTTGCAATAGAGTCTTCATAGTATAGATATCAGTAAGCTTTGGAATAT 1129

| || |||| | | ||| | ||||| || | | |||||| || ||||||||||

Sbjct 207356 CA---CCATGATGGCGTAGCAGACTATTCATTGTTTCTACATCAGTGAGTTTTGGAATAT 207412

Query 1130 TTATTAAGTTAAGATTTCCCTCTGCA---AGCAAAGCTGCAGTTATTATTGGAAGGGCAG 1186

| | || || ||| |||||| || | || || | |||||||| | ||||

Sbjct 207413 TAGTCAATACAA---TTCGATCTGCAGTTAGTAGTGCAGCTGCCATTATTGGTAAAGCAG 207469

Query 1187 CATTTTTGGCACCACTAATATTAACTTTGCCTATCAGCGGCTTGCCACCTTTAATTATTA 1246

||||||| ||||| |||| |||||| | ||| || ||| | ||||||||||| | ||

Sbjct 207470 CATTTTTAGCACCGTTAATTTTAACTATTCCTTGTAGAGGCGTTCCACCTTTAATGACTA 207529

**murA <**

Query 1247 TACTATCCATGTGCAATATATATTTCTAAATTCTTGACTATATTAGAGaaaaaaaTCATC 1306

|||||||||| ||||||| || || ||| | | |||| | ||||| |

Sbjct 207530 TACTATCCAT--------TATATTTATACATG-TTG--TTTGTTAG-GTTTTAAATCTTT 207577

Query 1307 TCAGACAAGTTTAAATCACTTATTTAAATTATCTAAAGGCTATAGTCCTCGCA-TAGAAT 1365

| | || |||||| ||| ||| | || || || | | || | | |||

Sbjct 207578 TAACACTT-TTTAAACGTAATATATAATTGATTTATTGGTAAAATAAATCATAATTAAAT 207636

Query 1366 CAT-TATTTCAATATTCTTTATAATAATTTTCTTGAACTAAATTGGTCTTTATACTTTCT 1424

|| |||| | |||| | |||| ||| || ||| |||||| | ||| | || |||

Sbjct 207637 AATGTATTCC--TATTAAGAACTATAACTTTTTT-AAC-AAATTG-TATTTGTGCTATCT 207691

>>

Query 1425 TTTATTAATTTTATTATTATACTAAAGAGCATTTTAATTGAATCAAAGATTAATTG--AT 1482

||| | |||| |||| |||||| || |||| || | | || ||| | || ||

Sbjct 207692 TTTTTCTATTTAATTAATATACTGTAG--CATTCAAAAATTAGCTAAAATTTAGTGAAAT 207749

**> Acyl-CoA-carboxylase**

Query 1483 GAATCAAACCATTTTACCAGATCAAGACTTACTTGAGGAAAAACGCAGTATAGCGCGCGC 1542

|||||||| | || ||| || |||||| | ||||| |||||| | |||||||| | ||

Sbjct 207750 GAATCAAAACGTTCTACAAGGTCAAGATCTTCTTGAAGAAAAAAGAAGTATAGCTAGAGC 207809

Query 1543 CGGTGGTGGACATGATCGGATAGCACTGCAACATAAGAAAGGAAAGCTAACAGCTCGTCA 1602

||||| || | ||| || || || || |||||||| || ||| || || ||||| |

Sbjct 207810 TGGTGGGGGCCTTGAGCGTATCGCTCTTCAACATAAAAAGGGACGACTCACGGCTCGCGA 207869

Query 1603 GAGAGTTGAAGCTTTCCTTGATCCTGATAGTTTTGAAGAAACGGGCATGTTTGTCGAACA 1662

||| |||||| ||| | || || || |||||||||||||| |||||||||||||| ||

Sbjct 207870 AAGAATTGAAGTTTTATTAGACCCAGACAGTTTTGAAGAAACTGGCATGTTTGTCGAGCA 207929

Query 1663 TCGCTGCGATAATTTCGGTATGAAAGATAAAAAGTTTGCAGGTGACGGGGTTGTCACAGG 1722

| ||||| ||||| ||||||||||||||||| ||| ||||| || || || |||||

Sbjct 207930 CAGGTGCGACAATTTTGGTATGAAAGATAAAAAAACTGCTGGTGATGGAGTGGTGACAGG 207989

Query 1723 GCATGGCACAATCAATGGTCGCTTGGTTTTTATCTATAGTCAGGATTTTACCGTTCTTGG 1782

||||| || || ||||| ||||| || ||||| |||||||| |||||||| ||||||||

Sbjct 207990 TCATGGTACGATAAATGGCCGCTTAGTATTTATATATAGTCAAGATTTTACAGTTCTTGG 208049

Query 1783 TGGCTCACTCGGTGAATATCATGCTAAGAAAATTTGTAATGTTATAGATTCGGCTCTGCA 1842

||| ||||| || |||||||||||||| || ||||||| |||| | ||||| ||| ||||

Sbjct 208050 TGGGTCACTTGGCGAATATCATGCTAAAAAGATTTGTAGTGTTGTTGATTCAGCTTTGCA 208109

Query 1843 AGTTGGAGCTCCTGTTATTGGAATTAACGATTCCGGTGGCGCCCGAATTCAAGAGGGAGT 1902

|| ||| ||||| || || || || |||||||| || || || |||||||||| |||||

Sbjct 208110 AGCTGGTGCTCCAGTAATAGGTATAAACGATTCTGGAGGAGCAAGAATTCAAGAAGGAGT 208169

Query 1903 TGATGCGCTCGGTGGTTACGGTGAATTATTCCAACGTAATGTCATAGCTTCAGGCGTGAT 1962

|||||| || ||||| || ||||| |||| ||||| ||||| | |||||||| |||||

Sbjct 208170 TGATGCTCTTGGTGGATATGGTGAGCTATTTCAACGCAATGTTCTTGCTTCAGGTGTGAT 208229

Query 1963 TCCTCAGATTACTCTAATTATGGGCCCCTGCGCAGGCGGTGCCGTTTACTCCCCTGCACT 2022

||||| |||| || || ||||| || |||||||| || || || ||||| ||||| ||

Sbjct 208230 ACCTCAAATTAGCCTGATCATGGGTCCATGCGCAGGAGGCGCAGTATACTCACCTGCTCT 208289

Query 2023 AACTGACTTTATATTCATGGTCCGAGATTCATCTTATATGTTTGTTACAGGACCGGATGT 2082

||||| || ||||| ||||| || || || |||||||||||||| ||||| || || ||

Sbjct 208290 GACTGATTTCATATTTATGGTGCGTGACTCTTCTTATATGTTTGTCACAGGGCCTGAAGT 208349

Query 2083 GGTCAAAACTGTAACAGGCGAAGAAGTCAGCCAAGAGAAATTAGGTGGTGCGAGGATGCA 2142

|| ||| | || || || || ||||| |||||||| || ||||||||||| | || ||

Sbjct 208350 TGTTAAAGCAGTTACTGGTGAGGAAGTAAGCCAAGAAAAGTTAGGTGGTGCTCGAATACA 208409

Query 2143 TACTACTAAAAGCGGAGTAGCAGATTTAGCTTTTAAAAATGATATTGAAGCATTGCTTGA 2202

|||||| |||||||| |||| |||||| | ||||||||||| || ||||||||| | ||

Sbjct 208410 TACTACAAAAAGCGGCATAGCTGATTTATCATTTAAAAATGACATAGAAGCATTGTTAGA 208469

Query 2203 GACTAGAAGATTTTTTAATTTTCTACCGCTATCTAATCGCGCCCCTCTTCCTACAAGATA 2262

|| ||| || | ||||||||||| || || ||||||||||| ||| | |||| | |

Sbjct 208470 AACCAGACGACTGTTTAATTTTCTTCCTCTTTCTAATCGCGCTCCTTTGCCTAGTCGTTG 208529

Query 2263 TACTAAAGACCCAGCGGACCGAGTCGATATGTCACTCAATACCTTAGTACCG--CTTGCT 2320

||||||||||| || || |||| |||||||| | ||||| || || || | |||

Sbjct 208530 CACTAAAGACCCTGCCGATAGAGTAGATATGTCTTTAAATACTTTGGTTCCTAACGTGC- 208588

Query 2321 GCTAATAAATCTTATGATATGAAAGAGTTAATCCAACGTATTGTTGATGAAGGATAATTT 2380

| |||||||| || || |||||||||||||| | ||||| ||||| |||||| | |||

Sbjct 208589 -CAAATAAATCGTACGACATGAAAGAGTTAATTAAGCGTATAGTTGACGAAGGAGATTTT 208647

Query 2381 TTTGAACTTCAACCTGATTTTGCTAAAAATATCCTAATTGGTTTTGGCTATATGGAGGGA 2440

||||| ||||| | || |||||||||||||| | ||||| ||||| |||||||| |||

Sbjct 208648 TTTGAGCTTCATGCCGAATTTGCTAAAAATATTATTATTGGCTTTGGTTATATGGAAGGA 208707

Query 2441 AGATCAATAGGATTCGTTGCTAATCAACCTCTTTATTTAGCTGGCTGCTTAGATATTAAT 2500

||| | | || || ||||| || ||||| ||||| |||||||||| ||||||||||||

Sbjct 208708 AGACCGGTGGGTTTTGTTGCCAACCAACCACTTTACCTAGCTGGCTGTTTAGATATTAAT 208767

Query 2501 GCTTCACGCAAAGCGGCTCGGTTCATTCGTTTTTGCGATGCTTTTAATATTCCAATAGTT 2560

||||| | ||||| ||| |||| || |||||||| || ||||||||||| || ||| ||

Sbjct 208768 TCTTCAAGAAAAGCAGCTAGGTTTATACGTTTTTGTGACGCTTTTAATATACCTATAATT 208827

Query 2561 AGCCTGGTTGACGTCCCAGGATTCCTACCAGGCACTGCTCAAGAGCATGATGGCATAATA 2620

|| | ||||| || |||||||| |||| || ||| |||||| | |||| | ||

Sbjct 208828 AGTTTAGTTGATGTACCAGGATTTTTACCTGGTACTAATCAAGAATACAATGGGCTGATT 208887

Query 2621 AAACATGGCGCAAAGCTGCTTTATGCTTATGCTGAGGCCACTGTACCAAAAATCACGGTC 2680

|| ||||| ||||| || | || |||||||| |||||||||||||| || || || |||

Sbjct 208888 AAGCATGGAGCAAAACTTTTATACGCTTATGCAGAGGCCACTGTACCCAAGATTACAGTC 208947

Query 2681 ATTACCAGAAAAGCTTATGGCGGAGCTTATATTGTTATGAATTCTAAGCATTTACGTGGA 2740

|| ||||||||||||||||| ||||||||||| || |||||||| || || | ||||||

Sbjct 208948 ATCACCAGAAAAGCTTATGGTGGAGCTTATATAGTAATGAATTCAAAACACCTGCGTGGA 209007

Query 2741 GATGTAAATTATGCATGGGTTAATCCCGAAATTGCTGTCATGGGAGCTGAAGGAGCAGCA 2800

||||| |||||||| ||| ||||| | ||||| ||||| ||||| || |||||||| ||

Sbjct 209008 GATGTCAATTATGCTTGGATTAATTCAGAAATAGCTGTTATGGGCGCAGAAGGAGCGGCT 209067

Query 2801 GAGATAATTTTTAAAGAAGATTGCAAAGATTCAGATTTAAAGAAGAAAAAGATACAAGAG 2860

|| || || |||||| | || ||||||||| | || ||| || | | |||||||||

Sbjct 209068 GAAATTATATTTAAAAACGAATGCAAAGATCCTGAACAAAAAAAAATATTGATACAAGAA 209127

Query 2861 TATAAAGATACGGTTACCTCACTTTTCGTTGCAGCATCTCGCGGTTATCTAGATGATATT 2920

||||| || || |||| || | || | ||||| ||| | || || ||||||| ||

Sbjct 209128 TATAAGGACACTATTACTTCTCCATTTATAGCAGCGTCTAGAGGATACATAGATGACATC 209187

Query 2921 ATAAAGCCACAAAATACGAGATGGCGTATTTGCAAAGCTTTGAATTTTTTACAGGGTAAA 2980

|| || ||||||||||| | ||||| |||||||||| || |||||||||| | |||

Sbjct 209188 ATTAAACCACAAAATACTCGTTGGCGACTTTGCAAAGCATTAAATTTTTTACGTGAGAAA 209247

**> Acyl-CoA-carboxylase ***

Query 2981 AATACTCAAATGCCATGGAAAAAACATGACAACCTTCCTTTATGATATGTTAATATGAAA 3040

|| ||| | |||||||||||||||||||| ||||| | | || | | ||||| |

Sbjct 209248 AAAGTACAAGTTCCATGGAAAAAACATGACAATCTTCCGCTGTAATGTTTCAATAT--AT 209305

**> APMC-CoA-carboxylase**

Query 3041 TTAAGAATTAA--TAAATGAACAAGCCATTATTTGATAAAGTTTTAATTGCTAACCGAGG 3098

||| | | ||| | |||| || || ||||||||||||||||| || ||||| || ||

Sbjct 209306 TTAGGTAATAAATTCAATGTCTAAACCTTTATTTGATAAAGTTTTGATCGCTAATCGTGG 209365

Query 3099 TGAGATCTCCCTGAGAATCATGCGTTCTCTAAAGAAAATGGGCATAAAATCAGTTGCAGT 3158

|||||| | | ||||| ||||| || ||||| || ||||| || ||||||||||| |

Sbjct 209366 TGAGATTGCATTAAGAATAATGCGCTCACTAAAAAAGATGGGTATCAAATCAGTTGCTAT 209425

Query 3159 GTATTCTGAGGCAGATACCGGTTCTAAGCACGTTCAAGAAGCGGACGAAGCTTATTATGT 3218

|| || ||||| ||||| || | ||| || || | || || ||||| ||||||||

Sbjct 209426 ATACTCAGAGGCTGATACAAACTCAATGCATGTGCAGTATGCTGATGAAGCCTATTATGT 209485

Query 3219 TGGTAATTCCCCAGCTACTGAGAGTTATTTATCTATTAAAAATATTGTCAATGCAGCAAG 3278

|||||||| ||||| ||||| ||||||||||| || |||||||| | ||||| ||

Sbjct 209486 GGGTAATTCTCCAGCAACTGAAAGTTATTTATCAATAAAAAATATCATTAATGCTATTAG 209545

Query 3279 GGCAAGTGGAGCGCAGGCAGTGCATCCAGGTTATGGTTTTTTAGCAGAAAATTTTAACTT 3338

|| ||||| || || || || ||||| || ||||| |||||||| |||||| || ||

Sbjct 209546 AGCGAGTGGTGCTCAAGCTGTTCATCCTGGCTATGGATTTTTAGCCGAAAATGCAAATTT 209605

Query 3339 TGCTAATATCTTAAAAAGAGAAGGAATCACTTTGATAGGCCCTAGCGCTCAAGCTATCAA 3398

|||| ||||||||||||| || || || || || || || ||| ||||| ||

Sbjct 209606 CGCTAGCGCTCTAAAAAGAGAAGGCATTACCTTAATTGGTCCAAGTGCTGGTGCTATAAA 209665

Query 3399 GCAAATGGGTGATAAGATTGAGGCTaaaaaaaTAGCTACTGAAGCTGGTGTTACTACAGT 3458

|||||||| ||||| ||||| || |||||||| |||||||||||||| ||||| || ||

Sbjct 209666 ACAAATGGGCGATAAAATTGAAGCAAAAAAAATTGCTACTGAAGCTGGAGTTACCACTGT 209725

Query 3459 TCCAGGATATATGGGAATTATTGGCAATGTTAATCAAGCTATCTCTATTGCTGAAGAAAT 3518

|||||| || ||||| |||||| | ||| ||| |||||| || ||||| || ||||| ||

Sbjct 209726 TCCAGGTTACATGGGCATTATTCGTAATATTACTCAAGCAATATCTATAGCAGAAGAGAT 209785

Query 3519 AGGTTTTCCGGTTATTGTTAAAGCGGCGGCAGGTGGCGGAGGTCGTGGGATGAGAGTAGT 3578

||| ||||| || ||||||||||| || || ||||| || ||| | || |||||||||||

Sbjct 209786 AGGATTTCCAGTCATTGTTAAAGCAGCAGCTGGTGGTGGTGGTAGAGGCATGAGAGTAGT 209845

Query 3579 TAAAAATTCCTCAGAGATGGCTGCGGCATTTGAATCAGCAAAATTAGAAGCCGAGAATAG 3638

||||||||| ||||||||||| |||||||||||||| ||| | ||||| || |||||

Sbjct 209846 TAAAAATTCTGCAGAGATGGCTCAAGCATTTGAATCAGCTAAAATTGAAGCAGAAAATAG 209905

Query 3639 CTTTAATGATGGCAGAGTCTTCATCGAAAAATTAATTGAAAACCCGCGTCATATAGAAAT 3698

||| |||| || ||||| ||||| || | ||||||||||| || ||||||||||||||

Sbjct 209906 TTTTGATGACGGTAGAGTATTCATTGAGAGATTAATTGAAAGTCCACGTCATATAGAAAT 209965

Query 3699 TCAGTTAATAGCTGATCAATTTGGTAACGCTGTGTGTTTGGGTGAGCGTGAATGTTCTAT 3758

|||||| ||||||||||||||||||| || || |||||||| || | ||||| |||||

Sbjct 209966 CCAGTTATTAGCTGATCAATTTGGTAATGCAGTATGTTTGGGCGAAAGAGAATGCTCTAT 210025

Query 3759 TCAACGGCATCATCAAAAAATCATTGAAGAAGCGCCTAGTTCTTTTATAACTGAAGAAAT 3818

||| || |||||||||||||| |||||||||||||| ||| ||||| |||||||||||

Sbjct 210026 TCAGCGTCATCATCAAAAAATTATTGAAGAAGCGCCAAGTGTATTTATCACTGAAGAAAT 210085

Query 3819 TCGGCAGAAAATGTATGCAGAAGTTATAGCTCTCACACACAAAGTTGGATATTATTCTGC 3878

|| || | |||||||||||||| |||||| | | | |||||||||||||| || ||

Sbjct 210086 ACGTAAGCAGATGTATGCAGAAGTGATAGCTTTATCCAATAAAGTTGGATATTACTCAGC 210145

Query 3879 TGGTACTGTAGAATTTATAATGGATCCTGATAAGCATTACTATTTCATGGAGATGAATAC 3938

|||||| || || || || |||||| | | || || || ||||| ||||| ||||||||

Sbjct 210146 TGGTACGGTGGAGTTCATTATGGATTCCAAGAAACAATATTATTTTATGGAAATGAATAC 210205

Query 3939 CAGGTTGCAAGTTGAGCATCCGGTAACTGAATTAGTTACCGGTATTGATATTGTTGAAGA 3998

||| | |||||||| ||||| ||||||||| ||||||||||||||||||| |||||||

Sbjct 210206 TAGGCTTCAAGTTGAACATCCAGTAACTGAACTAGTTACCGGTATTGATATCATTGAAGA 210265

Query 3999 AATGATAAAAATTGCGGCTGGCGAGAAATTATCTTTTACTCAAGATGATATTAAATTAAA 4058

|||||||||||||| ||||| |||||||||||||| || ||||| ||||| ||| | ||

Sbjct 210266 GATGATAAAAATTGCTGCTGGAGAGAAATTATCTTTCACCCAAGAAGATATAAAAATTAA 210325

Query 4059 AGGTTGGGCAATAGAGTCCAGAATATGTTCTGAAGATCCAACTAGAGGATTTCTACCTTC 4118

||| |||||||| || | ||||| || || |||||||||||| |||| || |||| ||

Sbjct 210326 AGGCTGGGCAATTGAAGCAAGAATTTGCTCAGAAGATCCAACTCGAGGCTTCTTACCCTC 210385

Query 4119 GAGTGGTAGAATTACTGGATATGCAGAACCATTAAAAAGCCCACATATTCGAATTGATAG 4178

|| ||||||||||| | |||||| || ||||||||||| || | ||||| | |||||

Sbjct 210386 TAGCGGTAGAATTACAGAATATGCTGAGCCATTAAAAAGTCCCAACATTCGTGTAGATAG 210445

Query 4179 CGGTGTGAGTATTGGCGGTGAGGTCAGCATGTTCTATGATCCAATGATTGCCAAGCTTTG 4238

||| || || ||||||||| || || ||||| || ||||||||||| || || | ||

Sbjct 210446 CGGAGTTGGTGCTGGCGGTGAAGTTAGTATGTTTTACGATCCAATGATAGCAAAATTGTG 210505

Query 4239 CACTTATCATGACACAAGAGAACAAGCGATTGAAATTATGCAAACAGCTTTAAGTTCTTA 4298

||||||||||| |||| ||| || || ||| ||||| | ||| | ||||||||

Sbjct 210506 TACTTATCATGATGATAGAGCACAGGCCATAGAATGTATGCGTTCTGCTCTTAGTTCTTA 210565

Query 4299 TGTTATCCAAGGGATTTCTCATAATATCAGCTTTTTAGAAGCCGTTATGTCACATCCACG 4358

||| || ||||| || || ||||||||||||||||||||||| ||||| || |||| | |

Sbjct 210566 TGTAATTCAAGGTATCTCGCATAATATCAGCTTTTTAGAAGCAGTTATCTCTCATCAAAG 210625

Query 4359 ATTCATTAGTGGTAATATTAATACCGGTTTCATTGCAGAAGAATATCCCGCTGGTTTTTC 4418

||| ||| ||||||||| || || ||||| || || |||||||| || |||||||||||

Sbjct 210626 ATTTATTGATGGTAATATAAACACAGGTTTTATAGCTGAAGAATACCCTGCTGGTTTTTC 210685

Query 4419 AGGAGCGAGCCTCACTTCTGAAATTACTGATGTTTTCCTAGCAACAGCAATTTACACTTA 4478

|| || | || ||||| ||||| ||||||||||| || | || || || | ||||

Sbjct 210686 TGGCGCTACTCTTACTTCCGAAATAACTGATGTTTTTCTCTCTACTGCCATATTTGCTTA 210745

Query 4479 TATTACTGAACAAAAAAGAGCTGCCTCAATTGAAGGACAAATGGTTGATCAAGCCAGTAA 4538

||| ||||| |||||||||||| | ||||| ||| ||||| ||||||||| |||||

Sbjct 210746 TATAACTGAGCAAAAAAGAGCTTCTTCAATATCAGGTCAAATTATTGATCAAGTAAGTAA 210805

Query 4539 AATCGGTACCCGATGGGTAGTATCTATTGATGATCGTTTATTCCCTGTTTTAATTAAACC 4598

|| ||||| | |||||||||||||| |||||| ||||| || || ||||| || ||

Sbjct 210806 CATAGGTACTAGGTGGGTAGTATCTATAGATGATAAATTATTTCCAGTATTAATCAAGCC 210865

Query 4599 CGTGCCAGATGGTTATAATATAAGACAAGGTTATACTAGGATTTATATCCGCAGCAATTG 4658

||| | ||| || ||||| || |||||||| |||||||| |||||||| || || || ||

Sbjct 210866 CGTCCAAGAAGGCTATAACATCAGACAAGGCTATACTAGAATTTATATACGTAGTAACTG 210925

Query 4659 GAATTTAGGCAGCCACCTTTTCTCTGCCGTAGTTAATGGGAGAAAGGTTAACGTCAAAAT 4718

| | |||| || || | || ||||| ||||||| ||||| || || ||||||||

Sbjct 210926 GCACATAGGTAGTCATTTATTTTCTGCTACGATTAATGGTAGAAAAGTCAATGTCAAAAT 210985

Query 4719 CGAGAATATCTCTACTGGTTATAGACTTACCCATTCCGGAATAACTGTTAAAACATTTGT 4778

|| | ||| ||| || |||| | | || || || || || || |||||| | | |||

Sbjct 210986 AGAAAGTATACTTACAGGATATAAATTAACTCACTCAGGTATTACAGTTAAAGCTTATGT 211045

Query 4779 CCGCTCGCCACTCATGAGTGAGCTTGAATCGATTATGCCT-GTGAAATTGCAACTTGATG 4837

||||| ||| |||||||| |||||||| | |||| | || ||||| | || |||

Sbjct 211046 ACGCTCACCAAGAATGAGTGAACTTGAATCAGTAATGCTTGGTAAAATTAC-TGTTAATG 211104

Query 4838 ACCTGACTGAGCTCACTGCCCCTCTTGCTGGCCAAATTATTGCGGTTAAAGTCCAAGAAG 4897

| | | || || || || ||||| || || ||||| || || || ||| || ||||

Sbjct 211105 ATCAAGCAGAACTTACAGCTCCTCTAGCGGGACAAATAATAGCTGTAAAAATCAAAGAGC 211164

Query 4898 GTGATGAAGTTATCGTCAGTCAAGAAATAATGATTTTAACGGCTATGAAGATGGAAAATA 4957

|| ||||| | || |||||||| | || | ||||| |||||||| ||||||||||

Sbjct 211165 AAGAGGAAGTAGTGGTGGGTCAAGAATTGATAGTCTTAACAGCTATGAAAATGGAAAATA 211224

Query 4958 TAATTTTAGCAGAGTGTGCAGGAAAAATAGCTAAAATATTTGTTAAAGATAAAGACCAAG 5017

|||| | || ||| | || |||||||||||||||||| || |||| ||||| ||||

Sbjct 211225 TAATCCTTGCTGAGAGAAGCGGCAAAATAGCTAAAATATTTATTCAAGAGAAAGATCAAG 211284

Query 5018 TCTCTGCTGGACAAGTATTACTAGAATTTGAGT 5050

| || ||||| ||||| || | ||||||||||

Sbjct 211285 TATCAGCTGGTCAAGTTTTGATGGAATTTGAGT 211317

#### A2 x2170 Caedibacter varicaedens DNA, WGS contig: Cva_contig000098 ; Length: 14326,

Sequence ID: [BBVC01000098.1](https://www.ncbi.nlm.nih.gov/nuccore/BBVC01000098), including XspD and prepilin peptidase CDS1700/2188(78%)

Query 1 GTGCTTTCGGTTATAGGAACGAAAGGGTCAGCTTTATTGTCTACAATTGTGGCTTTCAGG 60

|| ||||| |||| ||||| |||| || |||||||| || ||||| ||||||||||||

Sbjct 13019 GTACTTTCTGTTACGGGAACAAAAGTATCCGCTTTATTATCCACAATGGTGGCTTTCAGG 12960

Query 61 AAAATAACTAACTCTGACACAACCCGATCGTCCGATTTTCCTTTTGCAAGATTTCCGAAC 120

|||||||| | |||||||||||| ||||| || ||||| |||||||||||||||| ||

Sbjct 12959 AAAATAACCAGCTCTGACACAACGCGATCCTCAGATTTGGCTTTTGCAAGATTTCCCAAT 12900

Query 121 AAAGGGACTTCACTGATATACAGTACGCCCGATTGATTATTTTTTGAAATTTATTCCATC 180

|||||||||||||||| || | ||||| ||||| |||||||||| | | ||||||

Sbjct 12899 AAAGGGACTTCACTGAGGTAGGGGACGCCTGATTGGTTATTTTTTGCGACCTCTTCCATA 12840

Query 181 AGCCCACCCATAACAATGAATTCGCCGGAGTTCATGCAAACGAGAGATTCGAACTCTTGT 240

|| || ||||| |||||||||||||| |||||||| |||||||||||||| |||||||||

Sbjct 12839 AGTCCTCCCATCACAATGAATTCGCCCGAGTTCATACAAACGAGAGATTCAAACTCTTGT 12780

Query 241 TTACGAACAACGGGAACATAAGATTGTTGTGTTTCTTTAGAAACGATAGCAACGGCAGGA 300

|| |||||||| |||||||| |||||||| | |||||| ||||| || ||||| |||||

Sbjct 12779 TTGCGAACAACAGGAACATAGGATTGTTGCGCTTCTTTGGAAACAATGGCAACCGCAGGG 12720

Query 301 TCGGGTTTTTCATCAACCACCCGTGAAATAGTAGGACGTAAAGATAAAACAATAGAGCCA 360

|| | |||||||| || || |||||||| || ||||| || ||||||||||||||||||

Sbjct 12719 TCTGCCTTTTCATCCACAACGCGTGAAATGGTGGGACGCAAGGATAAAACAATAGAGCCA 12660

Query 361 TCTGCGATATTAATCGAGGGGTGAACAACCATGATAAGACCAATAGAAACGGTGTGAACT 420

|| || | ||| || || || ||||| ||||||||||||||||| | ||| || || | |

Sbjct 12659 TCCGCAAGATTGATTGAAGGATGAACGACCATGATAAGACCAATGGGAACAGTATGGATT 12600

Query 421 TCACTGGACACGTATTCATATTCTCGGACGGAGTCATAACCGTAATCCCTATTCTAATTT 480

|||||||| || ||||||| || ||||| |||||||| || |||||||| || || ||

Sbjct 12599 TCACTGGATACATATTCATGCTCGCGGACAGAGTCATAGCCATAATCCCTGTTATAGTTG 12540

Query 481 CTCCGAAAATATACATAATTTGTCGCTACTTTTATGACTCCGAGTTCGTTATTCATAGCG 540

| |||||||| |||||||| || || ||||||||||| ||| |||| || |||||||||

Sbjct 12539 ATTCGAAAATAGACATAATTGGTGGCAACTTTTATGACGCCGGGTTCATTGTTCATAGCG 12480

Query 541 GTTAATCGTGGGCTGGACAAAGTTATGACCGTCCCGAATTTATTGATTAAACTCACGATC 600

|| ||||| |||||||| | ||| |||| ||||| ||||||||||| | || || ||

Sbjct 12479 GTCAATCGCGGGCTGGAAAGCGTTCTGACTGTCCCAAATTTATTGATCAGACCTACTATT 12420

Query 601 TCTGTTAACTGTTTGCCACTTGCTCCAAAAGTAAAAACATCGCGTACGATAGTAGCTTTT 660

|| || | ||||||||| || | || || || |||||||| || ||| || || |||

Sbjct 12419 TCAGTGAGCTGTTTGCCGCTGCCCCCGAAGGTGAAAACATCTCGCACGGGGGTGGCGTTT 12360

Query 661 TTATTAAAAGATCCGGGTGTTATAGTGTCGCCCATAGGGCTTTGAAGTACAAGGTCTCCC 720

|| ||||| ||||||||||| || ||||| |||| |||||||||||| ||||||||||||

Sbjct 12359 TTGTTAAAGGATCCGGGTGTGATCGTGTCTCCCAGAGGGCTTTGAAGAACAAGGTCTCCC 12300

Query 721 TTCAGTAAATTCCAGTTAATACCTGCTTGAAATTCGTCTTTGAGGATAACTTCAACAATT 780

|| || |||||||||||||| || || | |||||| || |||||||| ||||| || |||

Sbjct 12299 TTGAGCAAATTCCAGTTAATGCCGGCCTTAAATTCATCCTTGAGGATGACTTCGACGATT 12240

Query 781 TTAGCCTCGATGAGTACTTGGCTTGAGGTGCTGAGGCGCAATAATTTT-AAAAATGTTCT 839

||||| ||||| | || |||||||| |||||||| ||||||| |||| ||||||||||

Sbjct 12239 TTAGCTTCGATCAAGACCTGGCTTGATGTGCTGAGTCGCAATAGTTTTAAAAAATGTTCG 12180

Query 840 ACTTGTTGGTGTTGAGCCTGAGTGACGTGAATATAAATAATTCCGGCTTGTTTATGAAGA 899

||||||||||||||||||||||| | | || | | ||||||||||| ||||| || |

Sbjct 12179 ACTTGTTGGTGTTGAGCCTGAGTCCCATAAACAGAGATAATTCCGGCCTGTTTGTGTAAG 12120

Query 900 GAGTACGCAGATTTTTCCTGTTTCCCTCCTTTAGGTTCAGAATCCTGTAGAATTGTTGAA 959

||||| | |||||| |||||| || | || | ||||||| ||||| ||| ||||||

Sbjct 12119 GAGTAGGAAGATTTATCCTGTCCAACTTCCTTGGCATCAGAATTCTGTAAAATGGTTGAA 12060

Query 960 AGATTACTCTCTAGTTCTGCCCAGAAATCATTTTTCGTCTCGCCCGTTAACAAGGTATTA 1019

|| |||||||| ||||||||||||||||||||||| || || || || | |||||| ||

Sbjct 12059 AGGTTACTCTCCAGTTCTGCCCAGAAATCATTTTTTGTTTCACCTGTCAGCAAGGTGTTG 12000

Query 1020 GATCCATTATCGGCTGTATGGGCTTGCCCCTCGTTGGCGGTAAAAATATCCGTTGCAACT 1079

|| |||||||| ||||| |||| || ||||| | ||||||||| |||||||| |||

Sbjct 11999 GAGCCATTATCTGCTGTGCTACCTTGTCCTTCGTTTGTGGTAAAAATGTCCGTTGCGACT 11940

Query 1080 GAAATTCGACTTTGATTCTGTCGTGTCAAAGAAAGGAACTGAGCATTGTAATTAATTCAA 1139

||||| || |||||||| ||||| |||| ||||| ||||| ||||||||||||| ||

Sbjct 11939 GAAATACGGCTTTGATTTTGTCGGGTCAGGGAAAGAAACTGGGCATTGTAATTAAGCAAA 11880

Query 1140 TAGGGTTTATCAGGTTCAATTCGCAAAATATTATTCTCAATTTTATATCAAAGGCAAGTA 1199

|| || |||||||||||||||||||||||||||||||||||||| |||| ||||| ||

Sbjct 11879 TAAGGCTTATCAGGTTCAATTCGCAAAATATTATTCTCAATTTTGTATCGAAGGCGGTTA 11820

Query 1200 AGAGTGCAGAGTTCCTTGACAATATTTATAAGAGGGCGTTTGGTTGTATGAAGCGCAACG 1259

||||| || |||||| |||| |||| |||||||| || | ||||| ||||||||||||

Sbjct 11819 AGAGTACAAAGTTCCCTGACGATATCAATAAGAGGACGATGGGTTGCATGAAGCGCAACA 11760

Query 1260 CCGCCTCTTACCTCAGGATCAAGGGAAAGATCAACACCTCCTTGACGAGCTATTTTAAAT 1319

|||||| | || | ||| |||| |||||| |||||| | | || || ||||||| |||

Sbjct 11759 CCGCCTTTCACATTAGGGTCAATGGAAAGGTCAACATTTGCCTGTCGGGCTATTTGAAAG 11700

Query 1320 AAAACGTCCTTCAAGGGTACTTCTTCCGTTGTTGAAAGTGTTATAGGTTTTTTGAGGGCT 1379

|| ||||| || | ||| ||| ||| ||||| ||||| |||| ||| ||||| || |

Sbjct 11699 AAGACGTCTTTTAGGGGAACTGATTCAGTTGTCGAAAGCGTTACAGGATTTTTAAGAACC 11640

Query 1380 TCAGG-ATAAGGGGAACA-CTACTATTTTTTGCAAAAGGGCGCG-CTTTT--CTGATGTG 1434

|||| |||| ||||||| || | |||||| |||||| || | ||||| ||||| |

Sbjct 11639 GCAGGAATAACGGGAACATCTGAT-TTTTTTATAAAAGGACGTGTCTTTTTCTTGATG-G 11582

Query 1435 AGTAGATGTGGGTCTGAGGAGATTCTCTTCGATCTGTTGTTTTGTCATTTTTGTCGACGG 1494

| | || | | || | ||||||| ||||||||| ||| |||||||| ||| ||

Sbjct 11581 A-TGGA-GGAGTCCTACTTAAATTCTCTGCGATCTGTTTTTTCGTCATTTTAGTCACAGG 11524

Query 1495 ATCAAGTGCCAGATCATTTTCAGTATCCCAGGTGCGGCAACTGGATAGCATAAAAAGTAT 1554

|||||| ||||| ||||| |||| |||||| |||| || || | | |||||| ||

Sbjct 11523 ATCAAGAGCCAGGTCATTGTCAGGATCCCATGTGCTACAGGACGACAACGAAAAAAGCAT 11464

Query 1555 TAAGACGATGAGACATCCTCTTACGGAAAAATTCATGTTATCTCTTAGTTTTGTTTATTT 1614

| |||| || ||||||| | | ||| ||||| ||||||||| ||||||||| ||

Sbjct 11463 CAGGACGCAGATACATCCTATGGTAGGAAAGTTCATTTTATCTCTTGGTTTTGTTTGTTG 1140

**XpsD <**

Query 1615 TGGGTTGAAAATACAGAAAAGTTTATTAAATTGCCAGCATAAATTACACAAAGGAAGTTT 1674

| | ||||||||||||| | |||||| |||| ||||| |||||||| | ||||||

Sbjct 11403 TTCGCTGAAAATACAGAAGAATTTATTGAATTACCAGCTTAAATTACTGGGAAGAAGTTG 11344

Query 1675 AGACATGCAAGTTATTCATCATTCATCCGGA---AGTGGTTATTTTGATGT---AGAAAA 1728

|| ||||||| || ||||| ||| ||| | || | || ||||| | | ||||

Sbjct 11343 AGGCATGCAATCTA---GTCATTGATCGGGATTTATTGATGATCTTGATATACAACAAAA 11287

Query 1729 CAGGGTTTGTGTTGGCATTTTGTGCCTTCGTGAAGTATAGT-AGCTTGCTTAATTTTCAC 1787

|| ||| |||||||| ||| | ||||||| ||| | | ||| | |||||||||||

Sbjct 11286 TAGCGTTATTGTTGGCA-TTTACACATTCGTGAGGTAGATTGAGCCTTCTTAATTTTCAG 11228

Query 1788 CAGGTTATGTCT---AGAATGTCGTATGATTCCTTGTTATTATTGTTGCTGGTGATGGCC 1844

| |||| ||| | |||| ||||| || || ||| ||||| || | || ||

Sbjct 11227 GATGTTAATTCTATCATGATGTTATATGACTCTCTGGCATTGCTGTTGTTGCTCATAACC 11168

**> prepilin peptidase**

Query 1845 TGGATAGGCATCATTTATTATGATATGCGCTATAGAATAGTTCCTGACTTTCTAGTTTTA 1904

||| ||| || |||||||||||||||||||||||||| |||| || || ||| |

Sbjct 11167 TGGGGAGGGATTATTTATTATGATATGCGCTATAGAATTATTCCGGATGGACTTGTTCTG 11108

Query 1905 TCTCTCTTGATCTTAGGAATTTTGCATTATTCTCTTACCTATGGGCATTTCTTATGCGCT 1964

|| | ||||| |||| |||||||||||| | ||||||| || || |||||||||

Sbjct 11107 TCCTTGTTGATTCTAGGGTGCTTGCATTATTCTTTAACCTATGAACACTTTTTATGCGCT 11048

Query 1965 GTTATTCTTGGTGGAGGAGGAGCACTTTTAAAAATAAGCATGGAGAAAATTATGAATCAT 2024

| |||| || || ||||| |||||||| ||||||||||||||| || |||| |

Sbjct 11047 GGCGTTCTGGGATTGGGGGGAGCGCTTTTAAAGATAAGCATGGAGAAACTTTTGAACCGC 10988

Query 2025 CCTGCATTAGGATGGGGAGATGTGAAGCTGGCAGGCGTATTAGGTATCGGGATGGAGCCC 2084

| ||| || ||||||||||| ||||| ||||| | | || ||||| |||||| ||

Sbjct 10987 CTTGCGTTGGGATGGGGAGACGTGAAACTGGCGGCAGCTTTGGGTATAGGGATGATTCCA 10928

Query 2085 GAGCAAATTTCCCTTTTCTTGATCTATATAGGACTTGTAGGATGTGCTTgggg--gggTC 2142

|| ||||||||||||||| |||||| ||| | |||| ||| |||||| ||

Sbjct 10927 GAACAAATTTCCCTTTTCCTGATCTGCGCAGGCTTCATAGGGTGTATTTGGGGTCTTTTC 10868

Query 2143 CATAAAAGAATTTTGAAAGAACCTCTCT 2170

|||||| ||||||||||||| | ||||

Sbjct 10867 TATAAAATAATTTTGAAAGAATCGCTCT 10840

#### B2 x1920 Caedibacter varicaedens, WGS contig: Cva_contig000019; mfd - rimM - trmD - rpl19

Sequence ID: [BBVC01000019.1](https://www.ncbi.nlm.nih.gov/nuccore/BBVC01000019), Length: 53002, 1550/1902 (81 %)

Query 25 TTGCCTGATGAAGTAAGTAATCTTCTGAAGATATTGACGTTCAAGCAACTTTGTCGAAAA 84

|||||||||||||| |||||||| | || |||||| || | |||||||||||||| |||

Sbjct 27793 TTGCCTGATGAAGTGGGTAATCTTTTAAAAATATTGGCGCTTAAGCAACTTTGTCGGAAA 27852

Query 85 GCCGGAATTGAAAAACTCGATGCAGGTAAAAAAGGATGTGTCTTATCATTTTTTAATCAA 144

|| || ||||| ||||| ||||| || ||||||||||||| ||||| |||| ||||||

Sbjct 27853 GCAGGCATTGAGAAACTGGATGCTGGCGAAAAAGGATGTGTTTTATCCTTTTCTAATCAG 27912

Query 145 AGTTTTATCAATCCGCTGGCTCTGGTCTCCTATATTAATGAGCATCAAGGGATTATTCGA 204

|||||| |||||| |||||| | ||| | ||||||||| ||||||| |||| ||||||

Sbjct 27913 AGTTTTTCCAATCCTCTGGCTTTAGTCGCTTATATTAATCAGCATCAGGGGACGATTCGA 27972

Query 205 TTGCGTCCTGATCACAAACTTGTTTTCCTGAAAGTATGGAAAACTCCTCTTCTTAAGATG 264

|| || |||||||| || ||||||||||||||||| ||||||||||| ||| ||| ||

Sbjct 27973 TTTCGCCCTGATCAAAAGCTTGTTTTCCTGAAAGTGTGGAAAACTCCCTCTCTCAAGGTG 28032

Query 265 GAGGGCGTTAAGAAGATTTTACAAGAACTTGTTGCTTTGGCCACTCAGAAGT-AAAGATA 323

|| || ||||| |||||||| || || | || || | || | || || ||| |||

Sbjct 28033 GAAGGGGTTAAAAAGATTTTGCAGGAGTTAATTTCTCTTGCAGAT-AGCAGAGAAATATA 28091

Query 324 A---AGGATGGATTCTTCTTCAAAGATCTTGATGGGTTTTATCATGGGCGCTTTCGGTAT 380

| || ||| ||||||||||||| || |||||||||||||| |||||||||| |||||

Sbjct 28092 AGGCAGAATGAATTCTTCTTCAAAAATTTTGATGGGTTTTATTATGGGCGCTTGTGGTAT 28151

Query 381 TCGAGGCGGGATGCGCTTCAAATCTTATACAGAGTCTATCGGAAACCTTAAAACTTATAA 440

||| || |||||||| ||||| |||||| || || | | | |||||| | |||||

Sbjct 28152 TCGCGGGGGGATGCGATTCAAGTCTTATGGGGAATCCCCCAAAGATCTTAAATCCTATAA 28211

Query 441 AGTATTGCAGGATCAAACAGGCCATCAGC-GCTTCAAAATAATCCAACTATTGCCGTATA 499

||||| |||||||||||||| ||||| |||| |||||||| || ||||||||||||

Sbjct 28212 GGTATTTCAGGATCAAACAGGTTGTCAGCAGCTT-AAAATAATTCAGATATTGCCGTATA 28270

Query 500 AAGAAAATATTATTACGCTTTACCTGGAAGGAATTACCACCAGAAGTCAGGCAGAAGCAC 559

|||| ||||| |||||||||||||||||||| |||||||| |||||||| |||||||| |

Sbjct 28271 AAGAGAATATCATTACGCTTTACCTGGAAGGGATTACCACAAGAAGTCAAGCAGAAGCGC 28330

Query 560 TTAAAGGAGTATCTCTCTATATTGATCGTGTTCAGCTTAAAAAGCCTTCAAGAGAAGAAT 619

||| ||| | || || |||||||||| | |||||||||||||| ||||| ||| ||||

Sbjct 28331 TTAGGGGAATGTCCCTTTATATTGATCATACTCAGCTTAAAAAGCTTTCAAAAGATGAAT 28390

Query 620 TTTATTACTATGATTTAGAAGGATTGGTTGTCCAAGATGAGCATAACTGTAAAAGAGGTC 679

||||||| ||||||| ||||| ||| |||||| |||| | ||||| | || |

Sbjct 28391 TTTATTATCATGATTTGGAAGGGTTGATTGTCCGGAATGAAGAAAACTGCGTTATTGGAC 28450

Query 680 ATGTGAAAGCAGTCGTTAATTATGGAGCTGATTCTATTCTGAGCATTTGTCTTTTAGAAG 739

| || ||| |||| || | ||||| ||||||||||| || | |||||||||||||||

Sbjct 28451 AGGTAAAAACAGTTGTGAGCTATGGCGCTGATTCTATGTTGGGTGTTTGTCTTTTAGAAG 28510

Query 740 ATCTTTCTGCGGAGATCCTGGTTCCTTTTCGTAAGGAATTTGTCAAAGAAGTTAATCAAA 799

||| ||| | ||||||||| |||||||||| || |||||||| |||| ||||||||||

Sbjct 28511 ATCCCTCTTCAGAGATCCTGATTCCTTTTCGGAAAGAATTTGTAAAAGCAGTTAATCAAC 28570

Query 800 AGGAAAAGTATATTATTCTTGATACAGATTATATGCGGGCTTTGCTTGATTTGAATAGGT 859

||||||| |||||||| ||||||||||||||| ||| ||| || ||||| || || ||

Sbjct 28571 AGGAAAAATATATTATCCTTGATACAGATTATGTGCAGGCATTTCTTGACTTAAAAGGGC 28630

Query 860 AATCTCA-TGACGTTTGAAATGAAAATTTTTACATTATATCCTGAACATTTCCCTGGTCC 918

||||| | |||| |||| ||| |||||||||||||| || |||||| ||| || || ||

Sbjct 28631 AATCTTAATGACTTTTGCAATTAAAATTTTTACATTGTACCCTGAAATTTTTCCAGGCCC 28690

Query 919 CCAGCAATACTAACTTGTAGGAAAAGCTCTTCAAGaaaaaaaGTGGTCCCTTGAAACTGT 978

| ||||||| ||||| || || ||||| |||||||| ||||| ||||| |||||

Sbjct 28691 TCTTCAATACTCGCTTGTCGGGCGGGCGCTTCAGGAAAAAAAATGGTCTCTTGAGACTGT 28750

Query 979 TAATATCCGTGATTTTGCCTTTGATAGGCATAAAACCGTTGATGATACGGCGTGTGGGGG 1038

||||| ||||||||||| |||||| |||||||||||||||||||||| || || || ||

Sbjct 28751 CAATATTCGTGATTTTGCTTTTGATCGGCATAAAACCGTTGATGATACAGCCTGCGGAGG 28810

Query 1039 AGGCCCGGGAATGGTGATGCGCCCGGATGTAATAGACCGTGCTTTAAAATTTCATTATCC 1098

|||||| || ||||||||||| | ||||| || ||||||||||||||||||||||||||

Sbjct 28811 AGGCCCTGGGATGGTGATGCGTGCTGATGTTATCGACCGTGCTTTAAAATTTCATTATCC 28870

Query 1099 GACATTTTCAAAATCCTTGATCTATTTGTCGCCTCGTGGCATACCATTGACACAAGAATA 1158

||||||||||||||||||||||||||||| |||||||||||||||||||| |||||

Sbjct 28871 AGTGTTTTCAAAATCCTTGATCTATTTGTCGCCGCGTGGCATACCATTGACACAGGAATA 28930

Query 1159 TGTAAAAAAGCTTGCGCGAAGACCCTCTCTAGGGTTACTGTGTGGTCGATTCGAAGGGAT 1218

||||||||||||||||| |||||||||| | ||||||||||||||||||||||||||| |

Sbjct 28931 TGTAAAAAAGCTTGCGCAAAGACCCTCTGTGGGGTTACTGTGTGGTCGATTCGAAGGGGT 28990

Query 1219 TGATCAACGCATTCTTGATGCTTGGGAATTTGAAGAAGTGAGTATAGGTGATTTCATCCT 1278

||| |||||| |||||||||| |||||||||||||||||||| |||||||||||||||||

Sbjct 28991 TGACCAACGCGTTCTTGATGCGTGGGAATTTGAAGAAGTGAGCATAGGTGATTTCATCCT 29050

Query 1279 TACGGGTGGTGAATTACCTGCTATGGCTTTGATAGATGCGTGTGTGCGAATTTTACCGGG 1338

|||||| || ||||| ||||||||||||||||| |||||||| || || || | || ||

Sbjct 29051 TACGGGGGGCGAATTGCCTGCTATGGCTTTGATTGATGCGTGCGTACGGGTTCTGCCAGG 29110

Query 1339 GGTAATAGGCTCAAGTGAATCATTAGAGGAAGAGAGCTTTTCTCAAGGACTTTTGGAGTA 1398

|| |||||||| |||||| | |||||||| |||||||||||||| |||||||| ||

Sbjct 29111 TGTTATAGGCTCGGCTGAATCTCTGGAGGAAGAAAGCTTTTCTCAAGGGCTTTTGGAATA 29170

Query 1399 TCCTCAATATACACGACCTTGTACATGGGAAGGCCGAGAAGTTCCCGAGGTGTTGCTAAA 1458

||| || ||||| || || || |||||||| || |||||||| || | ||||| |

Sbjct 29171 TCCCCAGTATACGCGGCCCCGTGTGTGGGAAGGGCGTGAAGTTCCTGACATATTGCTTCA 29230

Query 1459 AGGCTATCATAAGCACATACGGTGTTGGCGTAAAGCGCAAGCTGAAGAGATTACTCGTAC 1518

|| | || | || ||||| |||||||| | |||||||||||||||||||| ||

Sbjct 29231 GGGGCACCACGAACAAATACGTCGTTGGCGTCAGGCGCAAGCTGAAGAGATTACGCGCGT 29290

Query 1519 TCGCAGACCTGATTTATGGGAAAAATACTGTGATGCACGAGATAAAGATAAAGGGTAAAA 1578

||||||||| ||||| ||| |||| || |||||| | ||||| ||||| ||||| ||||

Sbjct 29291 TCGCAGACCCGATTTGTGGAAAAAGTATTGTGATACGCGAGACAAAGAGAAAGGATAAAG 29350

Query 1579 GAAATGAACATTCTTCAACAATTTGAACAGGAACAATTGGCTAAGCTTGCTGCAAATAGT 1638

|||||||||||||||||||||||| ||||||||| || | |||||||| || |||| |

Sbjct 29351 AGAATGAACATTCTTCAACAATTTGAGCAGGAACAAGTGACCAAGCTTGCGGCGAATAAT 29410

Query 1639 CTGGTTCCTCAATTTTCAGCGGGCGATACGTTACGTATCCACGTGAAAGTAGTAGAAGGC 1698

| ||||| ||||||||||| |||||||| |||||||||||||| || || || |||||

Sbjct 29411 CCTGTTCCCCAATTTTCAGCAGGCGATACATTACGTATCCACGTAAAGGTGGTTGAAGGA 29470

Query 1699 GAAAGAGAGAGAACTCAAGCTTATGAGGGGGTCTGTATCGCAATAAAAAATGCGGGAATT 1758

|| ||||||| || || |||||||| || ||||||| || | ||||||||| ||||||

Sbjct 29471 GAGCGAGAGAGGACACAGGCTTATGAAGGATTCTGTATTGCGAGAAAAAATGCAGGAATT 29530

Query 1759 AATTCATCTGTGACGGTTAGAAAGCTTTCATTTGGTGAAGGAGTAGAACGTGTATTTCCT 1818

||||| || || || || ||||||||||| ||||| ||||| || || || ||||||||

Sbjct 29531 AATTCTTCCGTAACTGTCAGAAAGCTTTCCTTTGGAGAAGGTGTGGAGCGCGTATTTCCA 29590

Query 1819 TTATATTCTCCTAATATTCGCATTGAAGTCGTACGGCATGGTGATGTAAGGCGAGCAAAG 1878

||||||||||| |||||||| |||||||| || ||| ||||||| || | || ||||||

Sbjct 29591 TTATATTCTCCCAATATTCGTATTGAAGTGGTGCGGTATGGTGACGTGCGACGGGCAAAG 29650

Query 1879 CTTTATTATCTGCGTGAGCGTACAGGAAAAGCTGCACGTATT 1920

||||||||| | || | ||||| || || ||||| ||||||

Sbjct 29651 CTTTATTATTTACGCGCTCGTACGGGCAAGGCTGCGCGTATT 29692

C1 **Query: 9405..18313 chr5-cA x462 ,x984, x225, x2223, x185, x1269 Length: 8909**

>MAG: Candidatus Megaira endosymbiont of Mesostigma viride isolate MegNEIS296 chromosome, complete genome

Sequence ID: CP084576.1 Length: 1448425

Range 1: 1229124 to 1230392

Score:873 bits(967), Expect:0.0,

Identities:970/1279(76%), Gaps:19/1279(1%), Strand: Plus/Minus

Query 7124 TTAATTCGATCTTCAATTTCATGGCGAAAGTGGTGAATAAGTCCCTGAATTGGCCAAGCT 7183

|| |||| ||||||||| ||||||||||| || || | ||| ||||| ||||| ||

Sbjct 1230392 TTGATTCTATCTTCAATCTCATGGCGAAAATGCCTAACTAATCCTTGAATAGGCCACGCA 1230333

Query 7184 GCAGCATCACCAAGAGCGCAAATAGTATGTCCCTCTACTTGCTTAGTCACGTCGAGTAGT 7243

|| ||||| || ||||||||||| ||||| ||||| | ||| || | | || || | |

Sbjct 1230332 GCTGCATCGCCGAGAGCGCAAATTGTATGCCCCTCAATTTGTTTGCTTATATCCAGCAAT 1230273

Query 7244 TGATCAATTTCTTCCATTTTTGCTTGGCCTTTTACTAAACGCATCATTACCCGCCACATC 7303

| |||||||||||| ||||| |||| || || | |||||||| | |||||||||

Sbjct 1230272 TCATCAATTTCTTCAATTTTAGCTTCCCCCTTACAGAGGCGCATCATGATTCGCCACATC 1230213

Query 7304 CAACCGGTACCTTCTCTACAAGGAGTGCATTGTCCGCAAGATTCATACATATAAAATTTG 7363

||||| |||||||||| || || |||||||| || || ||||||| ||||||||||||

Sbjct 1230212 CAACCAGTACCTTCTCGGCACGGTGTGCATTGACCACATGATTCATGCATATAAAATTTA 1230153

Query 7364 CTAAGCCTCGCAATAGCATAAATAACATCAGTAGACTTGTCCATTACAATAATCCCGCCA 7423

|| | | |||||||||||||| | ||||| || || |||||||| |||| |||||

Sbjct 1230152 CTTAAACGGGCAATAGCATAAATTATGTCAGTTGATTTATCCATTACTATAACTCCGCCT 1230093

Query 7424 GTGCCAAGTCCTGAGCCGAGAGCTCTCAGGGTATCAAAATCCATAGTAACTGTTTCGCAC 7483

|| ||||||||||| || ||| | || | |||||||||||||||||| ||||| |||

Sbjct 1230092 GTACCAAGTCCTGAACCAGCAGCACGAAGAGCATCAAAATCCATAGTAACAGTTTCACAC 1230033

Query 7484 ATTTCTTTAGGGATCATAGGTACAGATGAACCACCAGGAATGATAGCTTTTAAATTATTC 7543

|||||||| | ||||| ||||| ||||| ||||||||||| | |||||||||||||| |

Sbjct 1230032 ATTTCTTTTGTAATCATCGGTACGGATGACCCACCAGGAATTACAGCTTTTAAATTATCC 1229973

Query 7544 CACCCACCACGCACGCCACCAGCATATTTTTCAATTAGCTCTTTAAGTGGAATTCCCATT 7603

|| || ||||| | || ||||||| ||||||||||| |||||| | |||||||||||

Sbjct 1229972 CAGCCGCCACGAATACCTCCAGCATGTTTTTCAATTAACTCTTTTAAAGGAATTCCCATA 1229913

Query 7604 GCCTCTTCAATATTGCAAGGCTGATTCACATGTCCTGAAATACAGTAGAGCTTAGTTCCT 7663

|| ||||| ||||| ||| ||||||||| ||||| || || | | || |||||

Sbjct 1229912 GCTTCTTCTATATTCTTAGGTGCATTCACATGCCCTGAGATGCAAAATAATTTTGTTCCA 1229853

Query 7664 GTATTATTAGGTTTGCCAATTCCAGCAAACCAACTGGTGCCTC--GCCTTAAAATAGTTG 7721

|||||||||||||| || || | |||||||| || |||| | |||||||||| ||||

Sbjct 1229852 GTATTATTAGGTTTACCGATAGCGGCAAACCA--TGATGCCCCACGCCTTAAAATCGTTG 1229795

Query 7722 GCACCACAGCTATAGATTCAACATTATTAATTGTTGTAGGGCAGCCATAAAGCCCAGTAC 7781

| || || || ||||| || |||||||||||||| ||||| || ||||| | |||| ||

Sbjct 1229794 GTACTACCGCAATAGACTCTACATTATTAATTGTAGTAGGACATCCATATAAGCCAGCAC 1229735

Query 7782 CTGCTGGGAAAGGAGGCTTTAAGCGT-GGCATTCCTTTTTTGCCCTCTAAACTCTCAAGC 7840

| || || ||||||||||| |||| | ||| ||| ||||| ||||||| || ||||||

Sbjct 1229734 CAGCAGGAAAAGGAGGCTT-AAGCCTAGGCTGTCCCTTTTTTCCCTCTAGGCTTTCAAGC 1229676

Query 7841 AGTGCCGTCTCCTCTCCACAAATATATGCGCCTGCACCGCGGTGAAGATATATATCCAAA 7900

|| || || || ||||| |||||||| || || || || | |||||||| ||||| |

Sbjct 1229675 AGAGCTGTTTCTTCTCCGCAAATATAAGCTCCCGCCCCTCTATGAAGATAGATATCTAGG 1229616

Query 7901 TCATAACCGGAACCGCAAGCATTTTTGCCAATTAAGCCAGCTCCATATGACTCATCGATA 7960

|||||||| ||||| ||||||||||| |||||||| || || | ||||||||

Sbjct 1229615 TCATAACCAGAACCACAAGCATTTTTACCAATTAATTTTTCTTGGTAAGCTTCATCGATT 1229556

Query 7961 GCAATTTGTATATTTGAAGCTTCATTGTAAAACTCCCCTCTAATATATATATAACAAACA 8020

|| ||| | | |||||||| |||||||| ||||| ||||||||||||||| |

Sbjct 1229555 GCTCGTTGGACCGCCAATGCTTCATTATAAAACTCACCTCTTATATATATATAACAACTA 1229496

Query 8021 TGAGCACCAACTGCATGACTCGCAAGTAAACAGCCTTCAATAAGCTTATGAGGTTCAAAT 8080

|||||||| | ||| | ||||| | || ||||| ||||| ||||| || ||| |

Sbjct 1229495 TGAGCACCTATTGCGACAGAAGCAAGCACGCACCCTTCTATAAGTTTATGGGGCTCATAC 1229436

Query 8081 CTTAGAATATCTCGGTCTTTACAAGTCCCAGGCTCGGATTCATCAGCATTAACTACCAAA 8140

|| |||||||| || ||||| ||||| || ||||| |||||||||||||||||||| |

Sbjct 1229435 CTAAGAATATCCCGATCTTTGCAAGTTCCCGGCTCAGATTCATCAGCATTAACTACTAGG 1229376

Query 8141 TAACTCGGTTT-CGTTGAACTTTTTGGCATAAAAGACCACTTCATACCAGTGGAAAAACC 8199

||| ||||| | ||| | || |||||||| ||||| |||||||| || ||||| ||

Sbjct 1229375 TAAGAGGGTTTGGGATGATC-CTTGGGCATAAATGACCATTTCATACCGGTAGAAAACCC 1229317

Query 8200 TGCGCCTCCTCGCCCGCGAAGTCCAGATTGTTTAACTTCCTCTATAATCCAATCCCTACC 8259

|| ||||||| || || | ||||| ||||| |||||| || |||||||| | ||

Sbjct 1229316 AGCTCCTCCTCTACCCCGTAAACCAGAAGATTTAATTTCCTCAATGATCCAATCACGTCC 1229257

Query 8260 TTTTACAATGAA-GTCTTTAGTTTTATCCCAATCTCC-TCTAGCTTTACTACTTAC---T 8314

|| |||| || | |||| || ||||||||||| || | | ||| || | | |

Sbjct 1229256 CTTAGCAATTAATGACTTT-GTATTATCCCAATCACCGTGCATTTTTGCTGATGCCAGTT 1229198

Query 8315 AAGTCAAATCCTTGCTCGCCATATAGATTAGTAAAAATTTTATCTTGTGCTTGTAACATG 8374

|| ||| |||| || ||||| |||||||| ||||||||||| |||||||||

Sbjct 1229197 CAGGAGAAT-ATTGC---CCGTATAGGTTAGTAAAGATTTTATCTTGCTGTTGTAACATC 1229142

Query 8375 ATTAACTCTTAAATAAATA 8393

|| | || |||| |||||

Sbjct 1229141 ATCACCTTCTAAA-AAATA 1229124

Range 2: 1244069 to 1245384

Score:691 bits(765), Expect:0.0,

Identities:957/1329(72%), Gaps:17/1329(1%), Strand: Plus/Minus

Query 4326 GAACTAAAAACTAATGCTGAG-AATTTAATTAG-TGTACTTACGGATTTTGGTGTTAAAG 4383

||| || || | || |||||| || || |||| ||| || ||||| || || ||||

Sbjct 1245384 GAATTAGAAGCCAAGGCTGAGGAACTT--TTAGCTGTTCTCGGTGATTTCGGCGTCAAAG 1245327

Query 4384 GACAAATAGTTGATGTAAGTCAAGGGCCAGTTGTGACTCTTTATGAACTCGAACCTGCCC 4443

|| || | | ||| |||| ||||| || || || || | |||||| | ||||| ||

Sbjct 1245326 GAAAAGTTATAGATATAAGACAAGGTCCGGTAGTAACGATGTATGAATTTGAACCGGCAG 1245267

Query 4444 CTGGTACTAAGTCATCACGGGTTGTAGGACTTTCAGATGATATTGCTAGGTCTCTATCTG 4503

| |||||||| |||||| | || ||||| | || |||||||| || | || |||| |

Sbjct 1245266 CCGGTACTAAATCATCAAGAGTAATAGGATTGTCCGATGATATCGCCCGTTCATTATCGG 1245207

Query 4504 CTTTCTCAACTAGAATTGCAGTAGTGCCTGGCCGCAATGCATTAGGGATTGAATTACCAA 4563

|| || || | |||||||| | || ||| | || || | || ||||| | || |

Sbjct 1245206 CTCATTCTACCCGTATTGCAGTTATACCAGGCAGAAACGCTCTGGGTATTGAGCTGCCTA 1245147

Query 4564 ACAAACAGCGGGCTTTTTTCTGTTTAAGAGAGCTTATTGAAACTCCTGAATATCAGGATC 4623

| |||||||| ||||| |||||| ||||| ||||||| |||| |||||||

Sbjct 1245146 ATAAACAGCGTATGTTTTTTCGTTTAAAGGAGCTAGTTGAAACAGAAGAATTCCAGGATC 1245087

Query 4624 CAAATATATTGCTACCGTTAATTTTAGGTAAAGAT-TTAGCTGGTAAACCATATGTGGCT 4682

|| || | |||| | ||| | || || ||| ||||| || || | || |||

Sbjct 1245086 AGAACGTAATATTACCTATTATTCTTGGCAAGGATCTTAGC-GGACGGCCTTTAGTAGCT 1245028

Query 4683 GATCTTGCTAAAATGCCGCACTTGCTTGTCGCAGGAACTACGGGTTCTGGTAAATCTGTT 4742

||||| || |||||||| ||| ||||||| || ||||| || ||||| |||||||| |||

Sbjct 1245027 GATCTGGCCAAAATGCCTCACCTGCTTGTAGCGGGAACAACCGGTTCGGGTAAATCGGTT 1244968

Query 4743 GCAATAAATGCTATGATTATGTCCCTTCTGTATCGATATACACCAGCAGAATGTAGGTTA 4802

|| || || |||||||| |||||| | || ||| ||| || || || |||||||| |

Sbjct 1244967 GCTATTAACGCTATGATAATGTCCTTACTTTATAAATACACTCCCGCCGAATGTAGAATG 1244908

Query 4803 ATAATGATCGACCCTAAAATGCTTGAATTATTGGTTTACGACAATATTCCTCATCTTCTA 4862

|||||||| || ||||||||||||||||||| ||| || ||||| ||||| ||| |

Sbjct 1244907 ATAATGATTGATCCTAAAATGCTTGAATTATCTTCTTATGATAATATACCTCACCTTATG 1244848

Query 4863 ACTCCTGTAGTTACAGGATCTGGGAAGGCAGTGGTAGCTTTGAAATGGGCAGTAAGAGAA 4922

|| ||||| ||||| | | | || || ||||| || || | || ||||| || | ||||

Sbjct 1244847 ACCCCTGTTGTTACTGAACCCGGAAAAGCAGTAGTTGCGCTTAAGTGGGCTGTTAAAGAA 1244788

Query 4923 ATGGAAAATCGCTACCGGTTAATGTCTAATGTGGGAGTCAGGAATATAGCAGGTTATAAT 4982

||||||||| | || || ||||||||| || | ||||| |||||||| || || ||||

Sbjct 1244787 ATGGAAAATAGATATCGTTTAATGTCTCATTTAGGAGTGAGGAATATTGCTAATTTTAAT 1244728

Query 4983 GCCAAAATAGCAGAAAGCT-TAAAAGAAGGAAAAAGTTTAGAGTGTGTTGTGCAGACTGG 5041

|| ||||| | | |||| |||||||||| ||| | || | || || |||||

Sbjct 1244727 GCAAAAATT-CTGGAAGCGGTAAAAGAAGGCAAAGTGCTGGAACGAAGAGTACAAACTGG 1244669

Query 5042 CTTTGATCCTGATACTGGCAAACCAATTTATAAATCTATACCAATAGCTATGAAGAAACT 5101

||||| || || || || |||||| ||| | ||||| | || || | |||| | ||| |

Sbjct 1244668 TTTTGACCCGGAAACAGGGAAACCAGTTTTTGAATCTGTGCCCATTGATATGCAAAAAAT 1244609

Query 5102 ACCATTTATTGTGGTAATAGTTGATGAAATGGCTGACTTAATGATAGTGGCTGGTAAAGA 5161

|| ||||| || |||||||||||||||||||||||| ||||| | || || || |||||

Sbjct 1244608 GCCGTTTATAGTAGTAATAGTTGATGAAATGGCTGACCTAATGCTTGTTGCCGGGAAAGA 1244549

Query 5162 TATAGAATCTTCTATTCAGCGTCTTGCACAAATGGCTAGGGCTGCTGGGATCCACATTAT 5221

||| ||| |||||||||||||| | || || ||||| |||||||||| || ||||||||

Sbjct 1244548 TATCGAAACTTCTATTCAGCGTTTAGCCCAGATGGCACGGGCTGCTGGTATTCACATTAT 1244489

Query 5222 TATGGCAACCCAGCGCCCATCAGTTGATGTTATCACTGGTGTTATCAAAGCAAACTTTCC 5281

||||||||| || | ||||| || ||||| || ||||| || || ||||| ||||||||

Sbjct 1244488 TATGGCAACTCAAAGACCATCGGTAGATGTAATTACTGGCGTAATAAAAGCTAACTTTCC 1244429

Query 5282 AAGCCGCATTAGTTTTAAGGTAACTTCAAAAATTGATAGCAGAACAATTTTAGGAGAGCA 5341

|||| | |||||||| || |||||||||||||| || || ||||||||| |||| || ||

Sbjct 1244428 AAGCAGAATTAGTTTCAAAGTAACTTCAAAAATCGACAGTAGAACAATTCTAGGTGAACA 1244369

Query 5342 AGGTTCAGAACAGCTGCTTGGTATGGGGGACATGTTATACATGGGTAACTCTTCGCGAAT 5401

|| | ||||| | || || ||||| || ||| | || ||||| || | ||||

Sbjct 1244368 GGGAGCTGAACAATTACTCGGGATGGGAGATATGCTGTATATGGGCAATAGCGCTAGAAT 1244309

Query 5402 TATTCGGGTGCATGGACCTTTCGTTGATGATAAGGAAGTTGAAAAAGTAACCAATTATTT 5461

|||| || || |||||||| |||||||| ||||| || ||||| ||||| || |

Sbjct 1244308 ACTTCGTGTCCACGGACCTTTTGTTGATGACAAGGAGGTAGAAAAGGTAACATCTTTCCT 1244249

Query 5462 AAGCAATACTGGTACCCCTGATTATGTCTCAGCTGTAATGGAAAGTACTGACGATGATAG 5521

|| | ||||||| ||||| ||| | || || || | ||| |||| || ||

Sbjct 1244248 CAGAAGTACTGGTGTTCCTGAATATATTTCTGCAGTTACTGAA---TCTGAAGAGGA--- 1244195

Query 5522 TATTAATATGGAAGATTTCAGAGACGGCGATGATGATGAAACTATTTATAAAAAAGCCAA 5581

| || | || || | | || ||| |||| || ||| ||||| ||||

Sbjct 1244194 TTTTGCTGCTGATGAACTAGGTGAATCAGATAATGACGA---TATGTATAAGCAAGCATT 1244138

Query 5582 ACAGATAGTTAAGATAGAGCGTAAAGTTTCAATTAGCTATATTCAAAGATGCCTGCGCAT 5641

| | || || | || || |||| |||||| || ||||||||||||| ||| || ||

Sbjct 1244137 AAATATTGTCAGGAATGAAAAGAAAGCTTCAATCAGTTATATTCAAAGATCCCTTCGAAT 1244078

Query 5642 CGGTTACAA 5650

|| || ||

Sbjct 1244077 TGGGTATAA 1244069

Range 3: 330270 to 330775

Score:257 bits(284), Expect:5e-68,

Identities:367/514(71%), Gaps:16/514(3%), Strand: Plus/Minus

Query 1150 GGTAGCGTGACTTTTCCTTCTAAAAAATCATCACCAATATTTTTACCGATTTCTGCATCA 1209

|||| || || |||||||| ||||||||| || ||||| || || | || || |

Sbjct 330775 GGTAAAGTAACCTTTCCTTCCGCAAAATCATCTCCTATATTCTTGCCTCTAACTTCACCT 330716

Query 1210 CTGCTTATATAATCTAGTAAATCATCTGCGATTTGAAAAATATTGCCTAAATTTATGCCA 1269

| | | ||||||||| ||||| |||| |||||||| || | || | || ||||||

Sbjct 330715 TGGGTAAAATAATCTAGCAAATCGTCTGTTATTTGAAATATTTCTCCCAGTTTCATGCCA 330656

Query 1270 AAATTCTTTAATGCATTACATATTTTATTATTTTGGCCTGAAATAATAGCGCCAACCTCA 1329

||||| | | |||||||| ||| | ||| || |||||||| |||||||||

Sbjct 330655 AAATTTCTCATGGCATTACAATAATTAGGAGATTGATTTGCTATAATAGCACCAACCTCA 330596

Query 1330 CAAGCTGCACCAAAAAGTTCTGCTGTTTTGGCTAGAATAATTTCCTGATATTCATCAAAA 1389

|| ||||| |||||||| | |||||||| || ||| | || | ||| || || |

Sbjct 330595 CATGCTGCTCCAAAAAGAACCGCTGTTTTTGCATTAATTACTTTCAAATACTCTTCTTCA 330536

Query 1390 GTAATAA---TACG-TCTTTGATTTAATTTAGCTAGTTGAGAGACTTCACCTTCTGCAAT 1445

|| || | |||| |||| || || |||| || || || |||||||||||| |

Sbjct 330535 GTTATTAACCTACGCTCTTCGAGTT----TAGCAAGCTGCGATACTTCACCTTCTATGAC 330480

Query 1446 TATTGACGAGGCTTTAGATAATGATTCTAATGCAGGAATAGATTTAACAGAT----ACCA 1501

| || | ||||| ||||| || | || || || | | | | | || |

Sbjct 330479 GACTGCGCACGCTTTCGATAAAACGTCCATAGCCGG----GAGTAACCTGGTGGCAACTA 330424

Query 1502 TTAATTTAAATGATTGACTAAAAAGAAAATCTCCTACTAAAATACTTGCTTTATTTCCCC 1561

|||||||||||||||||||||| || ||||| ||||| || |||||||||||||| ||||

Sbjct 330423 TTAATTTAAATGATTGACTAAATAGGAAATCACCTACCAATATACTTGCTTTATTACCCC 330364

Query 1562 AAATAATATTAGCTGTTGGTTTGAATCGGCGCATTTTACTATCATCTACGACATCATCAT 1621

| |||| ||||||||| ||| || || ||||| ||||| ||||||| ||||||||||

Sbjct 330363 ATATAACATTAGCTGTGGGTAAAAAACGACGCATCTTACTGCCATCTACTACATCATCAT 330304

Query 1622 GAAGTAAAGTAGCAGTATGGATAAACTCAACAGC 1655

| | ||| ||||| |||| ||||| ||||||||

Sbjct 330303 GCAATAAGGTAGCCATATGAATAAATTCAACAGC 330270

Range 4: 696538 to 696725

Score:104 bits(115), Expect:7e-22,

Identities:137/188(73%), Gaps:4/188(2%), Strand: Plus/Plus

Query 2744 TTAATAATAAT-GCATTTCTAACTATTTCTCTAAGATTGAAGAGTTTTTTGCTAATGTGT 2802

||| || |||| ||| |||| || | | ||| |||||| |||| || |||||||| | |

Sbjct 696538 TTATTACTAATAGCACTTCTTACAACTACTCGAAGATTCGAGAGCTTCTTGCTAATTTTT 696597

Query 2803 TCAGAATTGATCCCATTAATTTGAATAACCTGAAATAACTCACCATTTTTAGTTAAAAGA 2862

|| ||||| || |||||||| |||| || ||| | ||| ||||| |||||||| | | |

Sbjct 696598 TCTGAATTAATACCATTAATCTGAAAAATCTGTAGTAATTCACCGTTTTTAGTAAGTAAA 696657

Query 2863 GTATTTTCATTATAGTGGCAGGCGATGGGAATAAAATCTTCAG---AATTATTATATAAG 2919

||||| | || ||| ||||| || || || | || || || | || ||||| | |

Sbjct 696658 GTATTCTTATCATAATGGCATGCAATAGGTACGAAGTCCTCTGATAAAGCGTTATAAAGG 696717

Query 2920 TCTTTATC 2927

|||||||

Sbjct 696718 CCTTTATC 696725

Range 5: 540170 to 540402

Score:98.7 bits(108), Expect:3e-20,

Identities:166/236(70%), Gaps:6/236(2%), Strand: Plus/Minus

Query 292 CCAGTAATTCTGCCTGCAATTCTGTGAATAAATTCTATCCAAAAGATTTGCTTAAACTCA 351

|| ||||| | |||||||||| |||||||||||||| ||| || || ||||| ||

Sbjct 540402 CCGGTAATGCGTCCTGCAATTCGGTGAATAAATTCTAGCCAGAAAATGGATTTAAATTCT 540343

Query 352 TCTAAAGCCATATTTTTATTATGCTGCG-CATATTCAGGAGTTGCCTTGTATTTATTAAA 410

||||| |||||| ||||| | || | |||||| ||||| ||||||||||| ||

Sbjct 540342 GCTAAATTCATATTATTATT-TACTTTGATATATTCGGGAGTCTGCTTGTATTTATCGAA 540284

Query 411 TTCCAGGACCCAT--TGCCTTTCGCTCAAAGGAGGAAGCACCCCTGTTACAGGCTTCCAC 468

|| | |||| ||| ||| | |||||||| || | || ||||| || |||||

Sbjct 540283 CTCGCTTATCCATGATGC--TTCAGTTAAAGGAGGGAGAATTCCGGTTACGGGTTTCCAT 540226

Query 469 TCAACAATAGATAAGCCTGCGTCAGTGAGTCTTGTTAACCCTCCTAGCAATATCAT 524

|| |||||| || || | ||||| | | |||| ||| || | || |||||

Sbjct 540225 TCTGTAATAGACAAACCAGAATCAGTTAAACGAGTTAGCCCGCCAATAAAAATCAT 540170

Range 6: 1409800 to 1409911

Score:74.3 bits(81), Expect:1e-12,

Identities:85/112(76%), Gaps:2/112(1%), Strand: Plus/Minus

Query 2590 TTTTTATACCAAAGTTCGAAACCTTTTAGAAAATCATCAACAAAACAAAATAATGTGTCA 2649

|| |||||||| | || || ||||| | ||||||| |||||||| ||||| | ||

Sbjct 1409911 TTGTTATACCATGGCTCAAATCCTTTATAACAATCATCGACAAAACAGAATAAACTATCG 1409852

Query 2650 AAATCTATATTCATTGGGGT-ATCTTTATTTGTAAAAAACAT-AGAATACCT 2699

|||||||| ||||| |||| |||||| || | ||||| | |||||||||

Sbjct 1409851 TAATCTATACTCATTAGGGTAATCTTTTTTCTTGAAAAATTTCAGAATACCT 1409800

Range 7: 1368286 to 1368396

Score:72.5 bits(79), Expect:4e-12,

Identities:84/111(76%), Gaps:2/111(1%), Strand: Plus/Minus

Query 2590 TTTTTATACCAAAGTTCGAAACCTTTTAGAAAATCATCAACAAAACAAAATAATGTGTCA 2649

|| |||||||| || || ||||| ||||||||| ||||| || ||||| | ||

Sbjct 1368396 TTGTTATACCATGACTCAAATCCTTTATAAAAATCATCGACAAATCAGAATAAACTATCG 1368337

Query 2650 AAATCTATATTCATTGGGGT-ATCTTTATTTGTAAAAAAC-ATAGAATACC 2698

|||||||||||||| |||| |||||| || | | |||| |||||||||

Sbjct 1368336 TAATCTATATTCATTAGGGTAATCTTTTTTGTTGAGAAACTCTAGAATACC 1368286

Range 8: 1274160 to 1274240

Score:70.7 bits(77), Expect:1e-11,

Identities:64/81(79%), Gaps:0/81(0%), Strand: Plus/Minus

Query 8457 CATTTTGCCGAGTTCCTTAGAGAAAGTTGTCTCGCGCCCCTAGGTATTCTCTACCTACCC 8516

|| |||||| |||||||| | ||| |||| || ||| ||||| |||||||| ||

Sbjct 1274240 CAATTTGCCTAGTTCCTTCAGCATCGTTCTCTCAAGCGCCTTGGTATACTCTACCTGTCC 1274181

Query 8517 ACCTGTGTCGGTTTCGGGTAC 8537

|||||||||||||| ||||||

Sbjct 1274180 ACCTGTGTCGGTTTAGGGTAC 1274160

Range 9: 1273970 to 1274060

Score:66.2 bits(72), Expect:2e-10,

Identities:69/91(76%), Gaps:0/91(0%), Strand: Plus/Minus

Query 8755 GGAATATTAACCCGTTGCCCATCGACTACGCCTCTCGGCCTGATCTTAGGACCTGACTCA 8814

|||||||||||| | | |||||||||||||||| || |||| |||||| | |||| |

Sbjct 1274060 GGAATATTAACCTGATTCCCATCGACTACGCCTTTCAGCCTCGCCTTAGGGGCCGACTAA 1274001

Query 8815 CCCTCCGTGGACGAACCTTGCGGAGGAACCC 8845

|||| || || | | || || ||||| ||

Sbjct 1274000 CCCTGCGCAGATTAGCTTTACGCAGGAAACC 1273970

Range 10: 139792 to 139885

Score:58.1 bits(63), Expect:9e-08,

Identities:72/95(76%), Gaps:3/95(3%), Strand: Plus/Minus

Query 2608 AAACCTTTTAGAAAATCATCAACAAAACAAAATAATGTGTCAAAATCTATATTCATTGGG 2667

|||| ||||| ||||||||| ||||| ||| | | || ||||||| | |||| | ||

Sbjct 139885 AAACTTTTTATAAAATCATCGACAAAGCAATAAATTGCTATAAAATCTTTTTTCA-TAGG 139827

Query 2668 GTATCTTTA-TTTGTAAAAAACATAGAAT-ACCTA 2700

||| |||| || || ||||||| | ||| |||||

Sbjct 139826 TTATTTTTAGTTGGTGAAAAACACAAAATAACCTA 139792

Range 11: 1245972 to 1246067

Score:58.1 bits(63), Expect:9e-08,

Identities:72/98(73%), Gaps:2/98(2%), Strand: Plus/Minus

Query 3676 TAGGTCACTTTGGTTCATatttagcagattttttatatcagttatttggccttgcctctt 3735

||||| | |||||||| ||| |||| ||| | || ||||| | ||||| |||| ||||

Sbjct 1246067 TAGGTTATTTTGGTTCTTATCTAGCTGATATATTTTATCAACTTTTTGGAGTTGCATCTT 1246008

Query 3736 atatttttccactttgttttttctcttttgcttttatc 3773

|||| ||||| |||| ||| | | || | |||||

Sbjct 1246007 TTATTATTCCA--TTGTCATTTATTGTCTGGTCTTATC 1245972

Range 12: 844712 to 844807

Score:52.7 bits(57), Expect:4e-06,

Identities:72/96(75%), Gaps:10/96(10%), Strand: Plus/Plus

Query 6490 ATTATAGCTG-TCATTGGT--GGTTTTCTTAACTCCCCGATCACCA--AAGAGATTTC-T 6543

|||||||||| || || || |||||| | | |||||||||||||| || | ||| |

Sbjct 844712 ATTATAGCTGCTCGTTTGTACGGTTTTTTCAGCTCCCCGATCACCAATAACCAAGTTCTT 844771

Query 6544 TTGGTGATCAAACTTAA----AAACTTTAAGTTTAA 6575

|||||| || ||||||| | |||||||||||

Sbjct 844772 TTGGTGGTCGAACTTAATTTCATTTTTTAAGTTTAA 844807

C2 **Query: 28139..52905 x306, x2293, x1435, x210, x633, x3215, x1066 chr2-cF Length: 24767**

>MAG: Candidatus Megaira endosymbiont of Mesostigma viride isolate MegNEIS296 chromosome, complete genome

Sequence ID: CP084576.1 Length: 1448425

Range 1: 1281770 to 1284658

Score:1263 bits(1400), Expect:0.0,

Identities:2052/2936(70%), Gaps:88/2936(2%), Strand: Plus/Minus

Query 7959 AATAATGAATTTATATTTTATGATGGCCCACCTTTTGCTAATGGATTGCCTCATTATGGT 8018

|||||||||||||| ||||| ||||| ||||||||||||||||| || |||||||||||

Sbjct 1284658 AATAATGAATTTATTTTTTACGATGGTCCACCTTTTGCTAATGGTTTACCTCATTATGGA 1284599

Query 8019 CATTTGCTAACTGGATTTATTAAAGACGTTTATGCAAGATACCAGGCAACTAAAGGCAAA 8078

||||| ||||||| ||||||||||| || | ||| | || || | || ||||| |||

Sbjct 1284598 CATTTATTAACTGGTTTTATTAAAGATGTATTTGCCCGTTATCAAACTACCAAAGGTAAA 1284539

Query 8079 AAAGTTGAGCGCCGCTTTGGATGGGATTGCCATGGGTTGCCTGCAGAAATGGGTGCAGAA 8138

|| || || | | || || |||||||| ||||| | || ||||||||||| ||||||

Sbjct 1284538 AAGGTAGAAAGGAGATTCGGGTGGGATTGTCATGGTCTTCCGGCAGAAATGGGGGCAGAA 1284479

Query 8139 AAGGAACTTGGTTTCTCAGGACGTTTAGCGATCACAAAATTTGGCATTGATAAATTTAAT 8198

|||||||| || || ||||| | |||| || || ||| ||| ||||||||||| ||

Sbjct 1284478 AAGGAACTGGGATTTTCAGGCAGAATAGCAATTTCAGAATATGGTATTGATAAATTCAAC 1284419

Query 8199 GAACATTGTCGCTCTTCTGTAATGAAATATGCTTCTGAGTGGGAGAAATATGTTAATAGA 8258

| ||||| |||| ||||| ||||||||| || ||| ||||| |||||||| || |

Sbjct 1284418 AATCATTGCCGCTTATCTGTCATGAAATATTCTAGTGATTGGGAAAAATATGTAAACCGC 1284359

Query 8259 CAAGCGCGTTGGGTAGATTTTAATAATTCTTATAAAACTATGGACTTAAG--TTTTATGG 8316

|| || | ||||| || || ||||||||||| ||||| ||||| |||| || |||||

Sbjct 1284358 CAGGCCAGGTGGGTTGACTTCAATAATTCTTACAAAACCATGGA--TAAGAATTATATGG 1284301

Query 8317 AATCAGTACTATGGGCTTTTAAGGAATTATATAAAAAAGGCCTTATCTATGAGTCAATGC 8376

|||| || || |||||||||||||| | | ||| ||||| | || ||||||||||||

Sbjct 1284300 AATCTGTCCTTTGGGCTTTTAAGGAGCTTTTTAATAAAGGGTTGATTTATGAGTCAATGA 1284241

Query 8377 GTGTGATGCCTTATTCATGGGCATGTGAAACGCCTCTATCTAATTTTGAAACTAGACTTG 8436

| || ||||| ||||| ||||| |||||||| ||| | |||||||||||||| ||| | |

Sbjct 1284240 GAGTAATGCCATATTCCTGGGCTTGTGAAACTCCTTTGTCTAATTTTGAAACGAGATTGG 1284181

Query 8437 ATAATTCGTATCGAGAGCGGGTAGATAAAGCTGTAACATGTAGCTTTACTCTAACAGATA 8496

||||||| || || || | ||||||||| || || ||||||| |||| ||||

Sbjct 1284180 ATAATTCTTACCGCCAGAGAACAGATAAAGCGGTTACTGTTAGCTTTGTACTAAGTGATA 1284121

Query 8497 AACCGGCAAAAGCACCAGCCGGCTTTAAAGAATATAAAATGCTTGCATGGACAACTACCC 8556

| || ||| ||||| | || |||| |||||||| ||| ||||| ||||| ||||| |

Sbjct 1284120 AGCCCCAAAATGCACCGGAAGGATTTAGAGAATATAGAATACTTGCCTGGACTACTACTC 1284061

Query 8557 CATGGACTCTTCCTTCAAATCTAGCGCTGGCAGTTGGGCCTGAGGTTAAATATATTTTCG 8616

| ||||| | |||||||||||||| || ||||| || |||| || || |

Sbjct 1284060 CTTGGACATTACCTTCAAATCTAGCCCTAGCAGTAAATAGCGATTTTAACTACGCATTTG 1284001

Query 8617 TCCCTAAAGAGGAGAATTGCTACATTATAGCAAAATTCGCTAGCTACAAATATGCGAAAG 8676

| || || | || | || ||||| || || || || | | ||||| ||||

Sbjct 1284000 TACCAAATGGAGATATCTGTTACATAATTGCTTCTTTTGCCGTTAATCACTATGCAAAAG 1283941

Query 8677 AGCTAGGCATCGAAGAAGG--AGTTAAATGTCAAGAATGT-AGAGGAAATGAATTAGATG 8733

| || || ||| ||| | | | | || | ||| | |||||| ||||||| |

Sbjct 1283940 AATTAAATATAGAAAAAGACAAAACAGACTTTAAAACTGTTAAAGGAAAGGAATTAGCAG 1283881

Query 8734 GTATAACTTATAAACCAGTATTTGATTATTTTGTGGATCATCCCAATAGCTTTAGAGTGC 8793

| ||| ||||| || | |||||||||||| | |||| || |||||||||||| |

Sbjct 1283880 GGTTAAAATATAAGCCTATCTTTGATTATTTTAAGAATCACCCAAATAGCTTTAGAATCT 1283821

Query 8794 TCTGTGCAGAATTTGTTGCTGAAGGTGATGGTACCGGTATTGTTCACTTAGCCCCAGGGT 8853

| | ||| ||||| | || || ||||| || || | || || | |||||||| |

Sbjct 1283820 TAACCGGAGATTTTGTAGTAGAGGGAGATGGCACAGGAGTCGTGCATATGGCCCCAGGTT 1283761

Query 8854 TTGGTGAAGATGATCAGGTAGTATGTGTGCAAGCTGGGATAGAATTAGTATGCCCAGTAG 8913

|||||||||| ||||| | |||| |||| || ||||| | || || || ||||

Sbjct 1283760 TTGGTGAAGACGATCAAATTTTATGCCAACAAGAGGGAATAGAGCTGGTTTGTCCGGTAG 1283701

Query 8914 ATAATGCTGGCAAATTTACCTCTGAAGTTTATGATTTTGTAGGCCTCAATGTTTTTGAGG 8973

||||||| || |||||||| ||||| ||| ||||||| | | | || ||||| |

Sbjct 1283700 ATAATGCCGGAAAATTTACTGCTGAAATTTCAGATTTTGCCGATATGCAAGTGTTTGAAG 1283641

Query 8974 CTAATGATAATATTACCATAAAGTTAAAAGAAATTGGCGCCTGGCTTAAAACTGAACAAG 9033

| | || ||||| ||||| ||||| | || ||||||||||| || |||

Sbjct 1283640 CAACCGACCCTATTATTATAAAATTAAAGAACCAAGGTAATTGGCTTAAAACCGAGCAAT 1283581

Query 9034 TTTTGCATAACTATCCTCATTGCTGGCGAACGGATACTCCGCTGATATATAAAGCAGTGT 9093

| | ||||| ||||| ||||||||||| || ||||| || |||| |||||||| ||

Sbjct 1283580 ATATTCATAATTATCCCCATTGCTGGCGGACAGATACCCCTTTGATTTATAAAGCCGTAC 1283521

Query 9094 CCTCATGGTATGTGGAGGTGACGAAATTCAAAGATAGAATGGTTGAATTAAACCAAGAAA 9153

| || |||||||| | || || |||| ||||||||||||||||||||||| ||| |||

Sbjct 1283520 CTTCTTGGTATGTCAAAGTTACAGAATTTAAAGATAGAATGGTTGAATTAAATCAACAAA 1283461

Query 9154 TCAACTGGATACCAAGTAACGTTAAGGATAATTTATTTGGCAAATGGTTAGAAAATGCCA 9213

| || ||||||||| | |||||||||||| || ||||| || ||| |||||||||| |

Sbjct 1283460 TTAATTGGATACCAGGCAACGTTAAGGATGGCTTGTTTGGTAAGTGGCTAGAAAATGCTA 1283401

Query 9214 GAGATTGGTCTATAAGTCGGAATAGATTCTGGGGTACACCAATTCCAATATGGCGTTCAA 9273

| || ||||||||||| | ||| | || ||||| ||||| ||||| ||||||| || |

Sbjct 1283400 GGGACTGGTCTATAAGCAGAAATCGTTTTTGGGGAACACCTATTCCGGTATGGCGGTCTA 1283341

Query 9274 ATGATCCTAAATATCCAAGAATTGATGTCTATGCCTCAGTTGAAGAACTCGAGAAAGATT 9333

||||||| ||||| || ||||||| || |||| ||| | ||| || || |||||||

Sbjct 1283340 ATGATCCCAAATACCCTCGAATTGACGTTTATGGCTCTTGCGCAGATCTTGAAAAAGATT 1283281

Query 9334 TTGGAGTAAAAGTTACTGACCTCCATAGGCCTTATATTGACCAGCTAACACGGGCAAATC 9393

||||||||||| || ||| | || | || | ||||| ||||| | ||||||

Sbjct 1283280 TTGGAGTAAAAATTGAAGACTTGCACAAACCCTTCATTGATGGATTAACAAGACCAAATC 1283221

Query 9394 CTGATGATCCAACTGGTAAATCAATTATGCAACGTGTTGAAGATGTTTTTGATTGTTGGT 9453

| ||||| ||||| || ||||| || ||| | | || || || || |||||||||||||

Sbjct 1283220 CGGATGACCCAACAGGCAAATCCATGATGGTAAGGGTAGAGGACGTATTTGATTGTTGGT 1283161

Query 9454 TTGAGAGTGGCTCTATGCCATATGGACAAGCACATTATCCATTTGAAAACAAAGAATGGT 9513

|||| || || || ||||| || || ||||| || ||||| |||||||||||||||||||

Sbjct 1283160 TTGAAAGCGGTTCGATGCCTTACGGGCAAGCTCACTATCCTTTTGAAAACAAAGAATGGT 1283101

Query 9514 TTGAGAAACATTTTCCTGCTGATTTTATCGTTGAATATTCAGCCCAAACTAGAGGCTGGT 9573

|||||| ||||||||||| |||||||| ||||| ||||| || ||||| || || ||||

Sbjct 1283100 TTGAGAGTCATTTTCCTGCCGATTTTATTGTTGAGTATTCTGCTCAAACCAGGGGTTGGT 1283041

Query 9574 TTTATACTTTAATGGTGCTATCAACCGCACTCTTTGATCGCCCACCATTCCTTAATTGTA 9633

||||||| |||||||| || || || ||||| ||||| ||||| |||||||| || || |

Sbjct 1283040 TTTATACATTAATGGTTCTCTCTACTGCACTTTTTGACCGCCCGCCATTCCTAAACTGCA 1282981

Query 9634 TTTGCCACGGGGTTATACTTGACTCAAGCAGCCAGAAATTGTCTAAAAAATTGCGTAACT 9693

| ||||||||||| ||||| ||||| | | |||| ||| | |||||| | ||| |

Sbjct 1282980 TCTGCCACGGGGTAATACTGGACTCTACCGGCCAAAAACTTTCTAAACGCCTCAATAATT 1282921

Query 9694 ACGCTGATCCACTTGAGCTTTTTGATAAATATGGCTCTGACGCTTTACGATTAACTATGC 9753

||||||| |||||||| | ||||||||||| ||| | || |||||| || | |||||||

Sbjct 1282920 ACGCTGACCCACTTGAATTGTTTGATAAATACGGCGCAGATGCTTTAAGAGTCACTATGC 1282861

Query 9754 TCTCATCAAATGTAGTTAAAGGACAGGAGCTATTGATCGATAAAGAAGGGAAAATGGTCT 9813

| || || ||||| || ||||| ||||| || | || |||||||| || |||||||| |

Sbjct 1282860 TTTCGTCTAATGTCGTCAAAGGGCAGGAATTACTTATTGATAAAGATGGAAAAATGGTTT 1282801

Query 9814 ACGAGACCTTAAGGATATTTATCAAACCTATTTGGAGCGCCTATCACTTTTTTACCCTGT 9873

|| |||| || |||||||||| || || |||| || ||||| ||||| | |

Sbjct 1282800 TTGATGCCTTGCGGTTATTTATCAAGCCAATATGGAATGCTTATCATTTTTTCTGTTTAT 1282741

Query 9874 ATGCTAATGCTGACGGAATT-AGGGGGGAGAATATATT---AGCAGCCGTCTCTAACTTA 9929

| || ||| ||||| ||| ||| || | ||||| ||||||

Sbjct 1282740 ACGCAAATCTCGACGGCATTAAGGCAAGATACGATATTTCAAGCAGC------------- 1282694

Query 9930 AATCGTAATGTTCTTGATCAGTATATTTTGGCTAAGCTGAAGACGGCAGTTAGCGGAATT 9989

||| | || ||||| || ||||| | ||||| || | | || || ||||

Sbjct 1282693 ------AATATCCTAGATCAATACATTTTATCCAAGCTTAAAATTACCGTAAGAAGAATA 1282640

Query 9990 G-CACAGGGATTAGATAATTTCAATACT-CAGATTGCATATTCAAATATCGCAAACTTTT 10047

| || | || | || | || ||||| ||| |||||| || || | | || |

Sbjct 1282639 GACAATAGTATGA-ATCAGTTTGATACTGTAGA-GGCATATGCAGCGATAACTGATTTCT 1282582

Query 10048 TCGAAGTACTAAACAATTGGTATATCCGGAGAAGCCG--GCATCGCTTTTGGAAAAGTGA 10105

| ||||| ||||| || |||||||| | |||| | ||| | || ||||||| |||

Sbjct 1282581 TTGAAGTGCTAAATAACTGGTATATTAGACGAAGTAGAAGCA--GGTTCTGGAAAACTGA 1282524

Query 10106 AAAAGATTCTGATAAGAGAATGGCTTATAATACGCTCTATACATGTTTGCAAATGATGTG 10165

| ||| | ||||| | ||||| ||||| ||||| ||||| | || | |||

Sbjct 1282523 GCAGGATGCGGATAAATTATCAGCTTACAATACTCTCTACACATGCCTTGAAGTTATGAT 1282464

Query 10166 CCAGGCTATGAGCTCACTAGCTCCTCTGATTATGGAGGATATTTATTTAGGATTAACAGG 10225

| || |||||||| || ||| | ||| || |||| ||| | |||||

Sbjct 1282463 GAAAGCAATGAGCTCGTTACTGCCTTTAATTTCAGAAAATATATATCGTAGCTTAAC--- 1282407

Query 10226 GGCAAATAATAGCGGAGGTAGTGTTCATCTTACTAATTTCCCTGACT-TAAATGAAATTG 10284

| |||| ||||||| ||| ||||| || || | || | || ||

Sbjct 1282406 ---------TGGCGGGCCATCTGTTCAT-TTAGAAATTTTCCCGAATGTAGAGGATATAA 1282357

Query 10285 AAGTAGACCCAAACCTGATTAAAAATATGGATCTCATACTTGATATTTGCAATTGTGCAC 10344

|| | || |||||| ||||| ||||||| || || ||||| || ||| |||

Sbjct 1282356 AAATTGAGGAAAACCTAGTTAAAGTAATGGATCAGATCCTAGATATCTGTAATGCTGCTT 1282297

Query 10345 TTTTTATTCGTAGTCAAGAAAATATACGTGTTAGACAACCTCTTGCGCAGATTACTCTT- 10403

| ||||| || ||| ||||||||| || | | ||||| || ||| |||| | |

Sbjct 1282296 TATTTATCCGCAGTGAAGAAAATACCCGCATCCGTCAACC-ATTAAGCAAATTATTAGTC 1282238

Query 10404 -ATCATTAAAGAA---------GTA--------AATAATTTAAGGGTTTTTGAAGATATA 10445

||| | | ||| ||| || || | | ||||||||| ||

Sbjct 1282237 CATCTTGAGGGAATAGATGAGGGTACTTACGATAACTATATTAAACAATTTGAAGATCTA 1282178

Query 10446 ATTAAAGATGAAATTAACATAAAATCAATAGTTTA-CTACAATAACTTAGAAAACTTCGC 10504

|||||||||||| |||| | ||||| ||| |||| | || | ||| || | | ||

Sbjct 1282177 ATTAAAGATGAAGTTAATGTCAAATCCATAATTTATCAGGAAGATGTTA-AAGAATATGC 1282119

Query 10505 CATCCGAAAATTATCAATTAACTTTCAACTAGTTGGCAAAAGATTGTCTAATAAAAT-AA 10563

| ||| | || ||||| || | | || | |||||||| || | | ||||| ||

Sbjct 1282118 AGACTTAAAGCTTTCTATTAATTTCCCATTACTGGGCAAAAGGTTACCAGAGAAAATGAA 1282059

Query 10564 AGAACATTATAACCGCTTCCAAAAAGGGTGAATGGCAACTCCAAG---GCGAGCAACTAG 10620

|||| || ||| | ||| ||| | || | ||||| | ||| ||| |||||

Sbjct 1282058 AGAA-ATAATAGCAGCTGCCAGAGCTGGGAACTGGCA---CGAAGCTAGCGGAAAACTAT 1282003

Query 10621 AGATCGCAGG---AGAAGTACTAAATTCAGAGGAATTTTCTATTGTTCTTGAACCTAAAG 10677

| | || ||| || || | || |||| | | | | | |||||||||

Sbjct 1282002 TTGTTTCTGGTATTGAATTATTA---CCGGAAGAATATACCCTGGCCATGGAACCTAAAA 1281946

Query 10678 GTGATATAAAAGGAGCTAAGGCATTGTCAGATAACTCTGGTTTAGTAATCCTGGATTTAG 10737

| |||||||| | || || || || ||| | |||||||| | || || | |

Sbjct 1281945 AAGGCATAAAAGGGGTAAAAGCTTTATCTAATAGCATAGGTTTAGTTAAGCTAGACCTTG 1281886

Query 10738 AGATTACAAAAGAATTGGAAGAAGAAGGCGTTGCTAGAGATTTAATCAGATTTATTCAAC 10797

| || || |||| | ||||||||||| | || | ||||| | |||||||| || |

Sbjct 1281885 AAATAACTAAAGGGCTTGAAGAAGAAGGAATAGCCCGTGATTTGGTTAGATTTATCCAGC 1281826

Query 10798 AAGCTAGGAAAGATGCTGGCTTTAATGTTTCAGATAGAATTGAGCTTGAAATTAAA 10853

|| | || ||||| || || ||| | || ||||| ||||| | || | ||||||

Sbjct 1281825 AAACAAGAAAAGAAGCAGGTTTTGAGGTATCAGACCGAATTAAACTAAATATTAAA 1281770

Range 2: 1229124 to 1230392

Score:873 bits(967), Expect:0.0,

Identities:970/1279(76%), Gaps:19/1279(1%), Strand: Plus/Minus

Query 19869 TTAATTCGATCTTCAATTTCATGGCGAAAGTGGTGAATAAGTCCCTGAATTGGCCAAGCT 19928

|| |||| ||||||||| ||||||||||| || || | ||| ||||| ||||| ||

Sbjct 1230392 TTGATTCTATCTTCAATCTCATGGCGAAAATGCCTAACTAATCCTTGAATAGGCCACGCA 1230333

Query 19929 GCAGCATCACCAAGAGCGCAAATAGTATGTCCCTCTACTTGCTTAGTCACGTCGAGTAGT 19988

|| ||||| || ||||||||||| ||||| ||||| | ||| || | | || || | |

Sbjct 1230332 GCTGCATCGCCGAGAGCGCAAATTGTATGCCCCTCAATTTGTTTGCTTATATCCAGCAAT 1230273

Query 19989 TGATCAATTTCTTCCATTTTTGCTTGGCCTTTTACTAAACGCATCATTACCCGCCACATC 20048

| |||||||||||| ||||| |||| || || | |||||||| | |||||||||

Sbjct 1230272 TCATCAATTTCTTCAATTTTAGCTTCCCCCTTACAGAGGCGCATCATGATTCGCCACATC 1230213

Query 20049 CAACCGGTACCTTCTCTACAAGGAGTGCATTGTCCGCAAGATTCATACATATAAAATTTG 20108

||||| |||||||||| || || |||||||| || || ||||||| ||||||||||||

Sbjct 1230212 CAACCAGTACCTTCTCGGCACGGTGTGCATTGACCACATGATTCATGCATATAAAATTTA 1230153

Query 20109 CTAAGCCTCGCAATAGCATAAATAACATCAGTAGACTTGTCCATTACAATAATCCCGCCA 20168

|| | | |||||||||||||| | ||||| || || |||||||| |||| |||||

Sbjct 1230152 CTTAAACGGGCAATAGCATAAATTATGTCAGTTGATTTATCCATTACTATAACTCCGCCT 1230093

Query 20169 GTGCCAAGTCCTGAGCCGAGAGCTCTCAGGGTATCAAAATCCATAGTAACTGTTTCGCAC 20228

|| ||||||||||| || ||| | || | |||||||||||||||||| ||||| |||

Sbjct 1230092 GTACCAAGTCCTGAACCAGCAGCACGAAGAGCATCAAAATCCATAGTAACAGTTTCACAC 1230033

Query 20229 ATTTCTTTAGGGATCATAGGTACAGATGAACCACCAGGAATGATAGCTTTTAAATTATTC 20288

|||||||| | ||||| ||||| ||||| ||||||||||| | |||||||||||||| |

Sbjct 1230032 ATTTCTTTTGTAATCATCGGTACGGATGACCCACCAGGAATTACAGCTTTTAAATTATCC 1229973

Query 20289 CACCCACCACGCACGCCACCAGCATATTTTTCAATTAGCTCTTTAAGTGGAATTCCCATT 20348

|| || ||||| | || ||||||| ||||||||||| |||||| | |||||||||||

Sbjct 1229972 CAGCCGCCACGAATACCTCCAGCATGTTTTTCAATTAACTCTTTTAAAGGAATTCCCATA 1229913

Query 20349 GCCTCTTCAATATTGCAAGGCTGATTCACATGTCCTGAAATACAGTAGAGCTTAGTTCCT 20408

|| ||||| ||||| ||| ||||||||| ||||| || || | | || |||||

Sbjct 1229912 GCTTCTTCTATATTCTTAGGTGCATTCACATGCCCTGAGATGCAAAATAATTTTGTTCCA 1229853

Query 20409 GTATTATTAGGTTTGCCAATTCCAGCAAACCAACTGGTGCCTC--GCCTTAAAATAGTTG 20466

|||||||||||||| || || | |||||||| || |||| | |||||||||| ||||

Sbjct 1229852 GTATTATTAGGTTTACCGATAGCGGCAAACCA--TGATGCCCCACGCCTTAAAATCGTTG 1229795

Query 20467 GCACCACAGCTATAGATTCAACATTATTAATTGTTGTAGGGCAGCCATAAAGCCCAGTAC 20526

| || || || ||||| || |||||||||||||| ||||| || ||||| | |||| ||

Sbjct 1229794 GTACTACCGCAATAGACTCTACATTATTAATTGTAGTAGGACATCCATATAAGCCAGCAC 1229735

Query 20527 CTGCTGGGAAAGGAGGCTTTAAGCGT-GGCATTCCTTTTTTGCCCTCTAAACTCTCAAGC 20585

| || || ||||||||||| |||| | ||| ||| ||||| ||||||| || ||||||

Sbjct 1229734 CAGCAGGAAAAGGAGGCTT-AAGCCTAGGCTGTCCCTTTTTTCCCTCTAGGCTTTCAAGC 1229676

Query 20586 AGTGCCGTCTCCTCTCCACAAATATATGCGCCTGCACCGCGGTGAAGATATATATCCAAA 20645

|| || || || ||||| |||||||| || || || || | |||||||| ||||| |

Sbjct 1229675 AGAGCTGTTTCTTCTCCGCAAATATAAGCTCCCGCCCCTCTATGAAGATAGATATCTAGG 1229616

Query 20646 TCATAACCGGAACCGCAAGCATTTTTGCCAATTAAGCCAGCTCCATATGACTCATCGATA 20705

|||||||| ||||| ||||||||||| |||||||| || || | ||||||||

Sbjct 1229615 TCATAACCAGAACCACAAGCATTTTTACCAATTAATTTTTCTTGGTAAGCTTCATCGATT 1229556

Query 20706 GCAATTTGTATATTTGAAGCTTCATTGTAAAACTCCCCTCTAATATATATATAACAAACA 20765

|| ||| | | |||||||| |||||||| ||||| ||||||||||||||| |

Sbjct 1229555 GCTCGTTGGACCGCCAATGCTTCATTATAAAACTCACCTCTTATATATATATAACAACTA 1229496

Query 20766 TGAGCACCAACTGCATGACTCGCAAGTAAACAGCCTTCAATAAGCTTATGAGGTTCAAAT 20825

|||||||| | ||| | ||||| | || ||||| ||||| ||||| || ||| |

Sbjct 1229495 TGAGCACCTATTGCGACAGAAGCAAGCACGCACCCTTCTATAAGTTTATGGGGCTCATAC 1229436

Query 20826 CTTAGAATATCTCGGTCTTTACAAGTCCCAGGCTCGGATTCATCAGCATTAACTACCAAA 20885

|| |||||||| || ||||| ||||| || ||||| |||||||||||||||||||| |

Sbjct 1229435 CTAAGAATATCCCGATCTTTGCAAGTTCCCGGCTCAGATTCATCAGCATTAACTACTAGG 1229376

Query 20886 TAACTCGGTTT-CGTTGAACTTTTTGGCATAAAAGACCACTTCATACCAGTGGAAAAACC 20944

||| ||||| | ||| | || |||||||| ||||| |||||||| || ||||| ||

Sbjct 1229375 TAAGAGGGTTTGGGATGATC-CTTGGGCATAAATGACCATTTCATACCGGTAGAAAACCC 1229317

Query 20945 TGCGCCTCCTCGCCCGCGAAGTCCAGATTGTTTAACTTCCTCTATAATCCAATCCCTACC 21004

|| ||||||| || || | ||||| ||||| |||||| || |||||||| | ||

Sbjct 1229316 AGCTCCTCCTCTACCCCGTAAACCAGAAGATTTAATTTCCTCAATGATCCAATCACGTCC 1229257

Query 21005 TTTTACAATGAA-GTCTTTAGTTTTATCCCAATCTCC-TCTAGCTTTACTACTTAC---T 21059

|| |||| || | |||| || ||||||||||| || | | ||| || | | |

Sbjct 1229256 CTTAGCAATTAATGACTTT-GTATTATCCCAATCACCGTGCATTTTTGCTGATGCCAGTT 1229198

Query 21060 AAGTCAAATCCTTGCTCGCCATATAGATTAGTAAAAATTTTATCTTGTGCTTGTAACATG 21119

|| ||| |||| || ||||| |||||||| ||||||||||| |||||||||

Sbjct 1229197 CAGGAGAAT-ATTGC---CCGTATAGGTTAGTAAAGATTTTATCTTGCTGTTGTAACATC 1229142

Query 21120 ATTAACTCTTAAATAAATA 21138

|| | || |||| |||||

Sbjct 1229141 ATCACCTTCTAAA-AAATA 1229124

Range 3: 1244069 to 1245384

Score:691 bits(765), Expect:0.0,

Identities:957/1329(72%), Gaps:17/1329(1%), Strand: Plus/Minus

Query 17071 GAACTAAAAACTAATGCTGAG-AATTTAATTAG-TGTACTTACGGATTTTGGTGTTAAAG 17128

||| || || | || |||||| || || |||| ||| || ||||| || || ||||

Sbjct 1245384 GAATTAGAAGCCAAGGCTGAGGAACTT--TTAGCTGTTCTCGGTGATTTCGGCGTCAAAG 1245327

Query 17129 GACAAATAGTTGATGTAAGTCAAGGGCCAGTTGTGACTCTTTATGAACTCGAACCTGCCC 17188

|| || | | ||| |||| ||||| || || || || | |||||| | ||||| ||

Sbjct 1245326 GAAAAGTTATAGATATAAGACAAGGTCCGGTAGTAACGATGTATGAATTTGAACCGGCAG 1245267

Query 17189 CTGGTACTAAGTCATCACGGGTTGTAGGACTTTCAGATGATATTGCTAGGTCTCTATCTG 17248

| |||||||| |||||| | || ||||| | || |||||||| || | || |||| |

Sbjct 1245266 CCGGTACTAAATCATCAAGAGTAATAGGATTGTCCGATGATATCGCCCGTTCATTATCGG 1245207

Query 17249 CTTTCTCAACTAGAATTGCAGTAGTGCCTGGCCGCAATGCATTAGGGATTGAATTACCAA 17308

|| || || | |||||||| | || ||| | || || | || ||||| | || |

Sbjct 1245206 CTCATTCTACCCGTATTGCAGTTATACCAGGCAGAAACGCTCTGGGTATTGAGCTGCCTA 1245147

Query 17309 ACAAACAGCGGGCTTTTTTCTGTTTAAGAGAGCTTATTGAAACTCCTGAATATCAGGATC 17368

| |||||||| ||||| |||||| ||||| ||||||| |||| |||||||

Sbjct 1245146 ATAAACAGCGTATGTTTTTTCGTTTAAAGGAGCTAGTTGAAACAGAAGAATTCCAGGATC 1245087

Query 17369 CAAATATATTGCTACCGTTAATTTTAGGTAAAGAT-TTAGCTGGTAAACCATATGTGGCT 17427

|| || | |||| | ||| | || || ||| ||||| || || | || |||

Sbjct 1245086 AGAACGTAATATTACCTATTATTCTTGGCAAGGATCTTAGC-GGACGGCCTTTAGTAGCT 1245028

Query 17428 GATCTTGCTAAAATGCCGCACTTGCTTGTCGCAGGAACTACGGGTTCTGGTAAATCTGTT 17487

||||| || |||||||| ||| ||||||| || ||||| || ||||| |||||||| |||

Sbjct 1245027 GATCTGGCCAAAATGCCTCACCTGCTTGTAGCGGGAACAACCGGTTCGGGTAAATCGGTT 1244968

Query 17488 GCAATAAATGCTATGATTATGTCCCTTCTGTATCGATATACACCAGCAGAATGTAGGTTA 17547

|| || || |||||||| |||||| | || ||| ||| || || || |||||||| |

Sbjct 1244967 GCTATTAACGCTATGATAATGTCCTTACTTTATAAATACACTCCCGCCGAATGTAGAATG 1244908

Query 17548 ATAATGATCGACCCTAAAATGCTTGAATTATTGGTTTACGACAATATTCCTCATCTTCTA 17607

|||||||| || ||||||||||||||||||| ||| || ||||| ||||| ||| |

Sbjct 1244907 ATAATGATTGATCCTAAAATGCTTGAATTATCTTCTTATGATAATATACCTCACCTTATG 1244848

Query 17608 ACTCCTGTAGTTACAGGATCTGGGAAGGCAGTGGTAGCTTTGAAATGGGCAGTAAGAGAA 17667

|| ||||| ||||| | | | || || ||||| || || | || ||||| || | ||||

Sbjct 1244847 ACCCCTGTTGTTACTGAACCCGGAAAAGCAGTAGTTGCGCTTAAGTGGGCTGTTAAAGAA 1244788

Query 17668 ATGGAAAATCGCTACCGGTTAATGTCTAATGTGGGAGTCAGGAATATAGCAGGTTATAAT 17727

||||||||| | || || ||||||||| || | ||||| |||||||| || || ||||

Sbjct 1244787 ATGGAAAATAGATATCGTTTAATGTCTCATTTAGGAGTGAGGAATATTGCTAATTTTAAT 1244728

Query 17728 GCCAAAATAGCAGAAAGCT-TAAAAGAAGGAAAAAGTTTAGAGTGTGTTGTGCAGACTGG 17786

|| ||||| | | |||| |||||||||| ||| | || | || || |||||

Sbjct 1244727 GCAAAAATT-CTGGAAGCGGTAAAAGAAGGCAAAGTGCTGGAACGAAGAGTACAAACTGG 1244669

Query 17787 CTTTGATCCTGATACTGGCAAACCAATTTATAAATCTATACCAATAGCTATGAAGAAACT 17846

||||| || || || || |||||| ||| | ||||| | || || | |||| | ||| |

Sbjct 1244668 TTTTGACCCGGAAACAGGGAAACCAGTTTTTGAATCTGTGCCCATTGATATGCAAAAAAT 1244609

Query 17847 ACCATTTATTGTGGTAATAGTTGATGAAATGGCTGACTTAATGATAGTGGCTGGTAAAGA 17906

|| ||||| || |||||||||||||||||||||||| ||||| | || || || |||||

Sbjct 1244608 GCCGTTTATAGTAGTAATAGTTGATGAAATGGCTGACCTAATGCTTGTTGCCGGGAAAGA 1244549

Query 17907 TATAGAATCTTCTATTCAGCGTCTTGCACAAATGGCTAGGGCTGCTGGGATCCACATTAT 17966

||| ||| |||||||||||||| | || || ||||| |||||||||| || ||||||||

Sbjct 1244548 TATCGAAACTTCTATTCAGCGTTTAGCCCAGATGGCACGGGCTGCTGGTATTCACATTAT 1244489

Query 17967 TATGGCAACCCAGCGCCCATCAGTTGATGTTATCACTGGTGTTATCAAAGCAAACTTTCC 18026

||||||||| || | ||||| || ||||| || ||||| || || ||||| ||||||||

Sbjct 1244488 TATGGCAACTCAAAGACCATCGGTAGATGTAATTACTGGCGTAATAAAAGCTAACTTTCC 1244429

Query 18027 AAGCCGCATTAGTTTTAAGGTAACTTCAAAAATTGATAGCAGAACAATTTTAGGAGAGCA 18086

|||| | |||||||| || |||||||||||||| || || ||||||||| |||| || ||

Sbjct 1244428 AAGCAGAATTAGTTTCAAAGTAACTTCAAAAATCGACAGTAGAACAATTCTAGGTGAACA 1244369

Query 18087 AGGTTCAGAACAGCTGCTTGGTATGGGGGACATGTTATACATGGGTAACTCTTCGCGAAT 18146

|| | ||||| | || || ||||| || ||| | || ||||| || | ||||

Sbjct 1244368 GGGAGCTGAACAATTACTCGGGATGGGAGATATGCTGTATATGGGCAATAGCGCTAGAAT 1244309

Query 18147 TATTCGGGTGCATGGACCTTTCGTTGATGATAAGGAAGTTGAAAAAGTAACCAATTATTT 18206

|||| || || |||||||| |||||||| ||||| || ||||| ||||| || |

Sbjct 1244308 ACTTCGTGTCCACGGACCTTTTGTTGATGACAAGGAGGTAGAAAAGGTAACATCTTTCCT 1244249

Query 18207 AAGCAATACTGGTACCCCTGATTATGTCTCAGCTGTAATGGAAAGTACTGACGATGATAG 18266

|| | ||||||| ||||| ||| | || || || | ||| |||| || ||

Sbjct 1244248 CAGAAGTACTGGTGTTCCTGAATATATTTCTGCAGTTACTGAA---TCTGAAGAGGA--- 1244195

Query 18267 TATTAATATGGAAGATTTCAGAGACGGCGATGATGATGAAACTATTTATAAAAAAGCCAA 18326

| || | || || | | || ||| |||| || ||| ||||| ||||

Sbjct 1244194 TTTTGCTGCTGATGAACTAGGTGAATCAGATAATGACGA---TATGTATAAGCAAGCATT 1244138

Query 18327 ACAGATAGTTAAGATAGAGCGTAAAGTTTCAATTAGCTATATTCAAAGATGCCTGCGCAT 18386

| | || || | || || |||| |||||| || ||||||||||||| ||| || ||

Sbjct 1244137 AAATATTGTCAGGAATGAAAAGAAAGCTTCAATCAGTTATATTCAAAGATCCCTTCGAAT 1244078

Query 18387 CGGTTACAA 18395

|| || ||

Sbjct 1244077 TGGGTATAA 1244069

Range 4: 330270 to 330775

Score:257 bits(284), Expect:1e-67,

Identities:367/514(71%), Gaps:16/514(3%), Strand: Plus/Minus

Query 13895 GGTAGCGTGACTTTTCCTTCTAAAAAATCATCACCAATATTTTTACCGATTTCTGCATCA 13954

|||| || || |||||||| ||||||||| || ||||| || || | || || |

Sbjct 330775 GGTAAAGTAACCTTTCCTTCCGCAAAATCATCTCCTATATTCTTGCCTCTAACTTCACCT 330716

Query 13955 CTGCTTATATAATCTAGTAAATCATCTGCGATTTGAAAAATATTGCCTAAATTTATGCCA 14014

| | | ||||||||| ||||| |||| |||||||| || | || | || ||||||

Sbjct 330715 TGGGTAAAATAATCTAGCAAATCGTCTGTTATTTGAAATATTTCTCCCAGTTTCATGCCA 330656

Query 14015 AAATTCTTTAATGCATTACATATTTTATTATTTTGGCCTGAAATAATAGCGCCAACCTCA 14074

||||| | | |||||||| ||| | ||| || |||||||| |||||||||

Sbjct 330655 AAATTTCTCATGGCATTACAATAATTAGGAGATTGATTTGCTATAATAGCACCAACCTCA 330596

Query 14075 CAAGCTGCACCAAAAAGTTCTGCTGTTTTGGCTAGAATAATTTCCTGATATTCATCAAAA 14134

|| ||||| |||||||| | |||||||| || ||| | || | ||| || || |

Sbjct 330595 CATGCTGCTCCAAAAAGAACCGCTGTTTTTGCATTAATTACTTTCAAATACTCTTCTTCA 330536

Query 14135 GTAATAA---TACG-TCTTTGATTTAATTTAGCTAGTTGAGAGACTTCACCTTCTGCAAT 14190

|| || | |||| |||| || || |||| || || || |||||||||||| |

Sbjct 330535 GTTATTAACCTACGCTCTTCGAGTT----TAGCAAGCTGCGATACTTCACCTTCTATGAC 330480

Query 14191 TATTGACGAGGCTTTAGATAATGATTCTAATGCAGGAATAGATTTAACAGAT----ACCA 14246

| || | ||||| ||||| || | || || || | | | | | || |

Sbjct 330479 GACTGCGCACGCTTTCGATAAAACGTCCATAGCCGG----GAGTAACCTGGTGGCAACTA 330424

Query 14247 TTAATTTAAATGATTGACTAAAAAGAAAATCTCCTACTAAAATACTTGCTTTATTTCCCC 14306

|||||||||||||||||||||| || ||||| ||||| || |||||||||||||| ||||

Sbjct 330423 TTAATTTAAATGATTGACTAAATAGGAAATCACCTACCAATATACTTGCTTTATTACCCC 330364

Query 14307 AAATAATATTAGCTGTTGGTTTGAATCGGCGCATTTTACTATCATCTACGACATCATCAT 14366

| |||| ||||||||| ||| || || ||||| ||||| ||||||| ||||||||||

Sbjct 330363 ATATAACATTAGCTGTGGGTAAAAAACGACGCATCTTACTGCCATCTACTACATCATCAT 330304

Query 14367 GAAGTAAAGTAGCAGTATGGATAAACTCAACAGC 14400

| | ||| ||||| |||| ||||| ||||||||

Sbjct 330303 GCAATAAGGTAGCCATATGAATAAATTCAACAGC 330270

Range 5: 1285988 to 1286138

Score:106 bits(117), Expect:6e-22,

Identities:114/151(75%), Gaps:0/151(0%), Strand: Plus/Minus

Query 6862 TCGTTCAGTTTCTTGCGCTTTACGAACGCGTTTTACTGAAGGGGGCTCATAGAAGCGAGA 6921

|| ||| ||||| ||| ||| ||||| ||||||||| || || ||||| || || ||

Sbjct 1286138 TCTTTCTGTTTCCTGCTTTTTGCGAACCTTTTTTACTGACGGTGGTTCATAAAAACGCGA 1286079

Query 6922 CATTTTCATTGCGCGAAAGACGAGCTCTCTTTGCATTTTCCTTTTCAGGTTTTTAATTGC 6981

||| ||||| || || ||||||||||| ||||| || |||||||| | | || ||

Sbjct 1286078 CATCTTCATCAAACGGAAAACGAGCTCTCTCTGCATCTTTCTTTTCAGATCTCGGATAGC 1286019

Query 6982 TTGCTCACCATTGCCAGCATGAACATTTACC 7012

|| ||||| || ||| |||||||||| |||

Sbjct 1286018 TTTTTCACCGTTTCCACCATGAACATTAACC 1285988

Range 6: 696538 to 696725

Score:104 bits(115), Expect:2e-21,

Identities:137/188(73%), Gaps:4/188(2%), Strand: Plus/Plus

Query 15489 TTAATAATAAT-GCATTTCTAACTATTTCTCTAAGATTGAAGAGTTTTTTGCTAATGTGT 15547

||| || |||| ||| |||| || | | ||| |||||| |||| || |||||||| | |

Sbjct 696538 TTATTACTAATAGCACTTCTTACAACTACTCGAAGATTCGAGAGCTTCTTGCTAATTTTT 696597

Query 15548 TCAGAATTGATCCCATTAATTTGAATAACCTGAAATAACTCACCATTTTTAGTTAAAAGA 15607

|| ||||| || |||||||| |||| || ||| | ||| ||||| |||||||| | | |

Sbjct 696598 TCTGAATTAATACCATTAATCTGAAAAATCTGTAGTAATTCACCGTTTTTAGTAAGTAAA 696657

Query 15608 GTATTTTCATTATAGTGGCAGGCGATGGGAATAAAATCTTCAG---AATTATTATATAAG 15664

||||| | || ||| ||||| || || || | || || || | || ||||| | |

Sbjct 696658 GTATTCTTATCATAATGGCATGCAATAGGTACGAAGTCCTCTGATAAAGCGTTATAAAGG 696717

Query 15665 TCTTTATC 15672

|||||||

Sbjct 696718 CCTTTATC 696725

Range 7: 540170 to 540402

Score:98.7 bits(108), Expect:8e-20,

Identities:166/236(70%), Gaps:6/236(2%), Strand: Plus/Minus

Query 13037 CCAGTAATTCTGCCTGCAATTCTGTGAATAAATTCTATCCAAAAGATTTGCTTAAACTCA 13096

|| ||||| | |||||||||| |||||||||||||| ||| || || ||||| ||

Sbjct 540402 CCGGTAATGCGTCCTGCAATTCGGTGAATAAATTCTAGCCAGAAAATGGATTTAAATTCT 540343

Query 13097 TCTAAAGCCATATTTTTATTATGCTGCG-CATATTCAGGAGTTGCCTTGTATTTATTAAA 13155

||||| |||||| ||||| | || | |||||| ||||| ||||||||||| ||

Sbjct 540342 GCTAAATTCATATTATTATT-TACTTTGATATATTCGGGAGTCTGCTTGTATTTATCGAA 540284

Query 13156 TTCCAGGACCCAT--TGCCTTTCGCTCAAAGGAGGAAGCACCCCTGTTACAGGCTTCCAC 13213

|| | |||| ||| ||| | |||||||| || | || ||||| || |||||

Sbjct 540283 CTCGCTTATCCATGATGC--TTCAGTTAAAGGAGGGAGAATTCCGGTTACGGGTTTCCAT 540226

Query 13214 TCAACAATAGATAAGCCTGCGTCAGTGAGTCTTGTTAACCCTCCTAGCAATATCAT 13269

|| |||||| || || | ||||| | | |||| ||| || | || |||||

Sbjct 540225 TCTGTAATAGACAAACCAGAATCAGTTAAACGAGTTAGCCCGCCAATAAAAATCAT 540170

Range 8: 771328 to 771587

Score:92.4 bits(101), Expect:1e-17,

Identities:178/260(68%), Gaps:6/260(2%), Strand: Plus/Minus

Query 4958 ATAAAATTTCC--ACGCGGCATT-ACAGGAGCTAACCTCGATATACTAGCCAGGCAATAT 5014

||||||||||| | | || | ||||| || || || ||||| || |||||||||| |

Sbjct 771587 ATAAAATTTCCGAAAGAATCAGTGACAGGTGCAAATCTTGATATCCTGGCCAGGCAATTT 771528

Query 5015 CTGTGGGCAGAAAGTTCAGATTATCCTCACTCAACCGGCCATGGCGTAGGAAGTTATTTA 5074

| ||| ||| | |||||||| || ||||||||||| || || |||| ||||

Sbjct 771527 TTATGGCAAGATGGGGACGATTATCCCCATGGTACCGGCCATGGAGTTGGTAGTTTTTTA 771468

Query 5075 AGCGTTCATGAGGGGCCACAGGGAATTAATTTAAGAAATAATG---TATCCCTTAAACCG 5131

|| ||||| || || ||||| || | |||| | | ||| | || | |

Sbjct 771467 AGTGTTCACGAAGGACCACAAAATATCAGTTTAGCCAGTTATGGCACAAAGCTAGCAGCT 771408

Query 5132 GGAATGATTCTATCCAATGAACCTGGGTATTATGTTCCGGGGAAGTTTGGCATAAGAATT 5191

|| ||| || | || |||||||| || | |||| || || | ||||| || |||||

Sbjct 771407 GGTATGGTTATCTCGAATGAACCAGGTTTTTATCAGCCAGGTGAATTTGGGATTAGAATA 771348

Query 5192 GAAAATTTAATGTATGTCAA 5211

|||||| | |||||||| ||

Sbjct 771347 GAAAATATGATGTATGTTAA 771328

Range 9: 1409800 to 1409911

Score:74.3 bits(81), Expect:3e-12,

Identities:85/112(76%), Gaps:2/112(1%), Strand: Plus/Minus

Query 15335 TTTTTATACCAAAGTTCGAAACCTTTTAGAAAATCATCAACAAAACAAAATAATGTGTCA 15394

|| |||||||| | || || ||||| | ||||||| |||||||| ||||| | ||

Sbjct 1409911 TTGTTATACCATGGCTCAAATCCTTTATAACAATCATCGACAAAACAGAATAAACTATCG 1409852

Query 15395 AAATCTATATTCATTGGGGT-ATCTTTATTTGTAAAAAACAT-AGAATACCT 15444

|||||||| ||||| |||| |||||| || | ||||| | |||||||||

Sbjct 1409851 TAATCTATACTCATTAGGGTAATCTTTTTTCTTGAAAAATTTCAGAATACCT 1409800

Range 10: 1265409 to 1265598

Score:72.5 bits(79), Expect:1e-11,

Identities:134/192(70%), Gaps:5/192(2%), Strand: Plus/Plus

Query 11976 AAACTAACGGAATGTGTGAGCGATTCCATAAAACTAT--AAAATGAATTCTTTGACATTG 12033

||||||| || || |||||||| ||||||||||| || || | || | | | || ||

Sbjct 1265409 AAACTAATGGCATTTGTGAGCGTTTCCATAAAACCATGCAAGAAGAGTGTTATCACCTTC 1265468

Query 12034 TCATGCGTAAAAAGATTTACACTTCTCTTAAAGAGCTACAACAGGATCTTGATACATGGC 12093

| | ||||||||| |||| | | | | | ||| |||| | ||| | ||||| |||

Sbjct 1265469 TATTCCGTAAAAAGTTTTATAATATTTTGGATGAGTTACATCTAGATGTAGATACCTGGA 1265528

Query 12094 TACATTA-TTATAACTATGAGGCGACCACATTATGGCAAGTATTACTATGGTAAAACTCC 12152

| |||| ||||| | || || | |||| || ||| ||| | ||| ||||||||

Sbjct 1265529 T-TATTAGCTATAATAACGA-AAGATCTCATTCAGGTAAGCATTGTTTTGGAAAAACTCC 1265586

Query 12153 TATGCAAACCTT 12164

|||||||| ||

Sbjct 1265587 AATGCAAACTTT 1265598

Range 11: 1368286 to 1368396

Score:72.5 bits(79), Expect:1e-11,

Identities:84/111(76%), Gaps:2/111(1%), Strand: Plus/Minus

Query 15335 TTTTTATACCAAAGTTCGAAACCTTTTAGAAAATCATCAACAAAACAAAATAATGTGTCA 15394

|| |||||||| || || ||||| ||||||||| ||||| || ||||| | ||

Sbjct 1368396 TTGTTATACCATGACTCAAATCCTTTATAAAAATCATCGACAAATCAGAATAAACTATCG 1368337

Query 15395 AAATCTATATTCATTGGGGT-ATCTTTATTTGTAAAAAAC-ATAGAATACC 15443

|||||||||||||| |||| |||||| || | | |||| |||||||||

Sbjct 1368336 TAATCTATATTCATTAGGGTAATCTTTTTTGTTGAGAAACTCTAGAATACC 1368286

Range 12: 1159594 to 1159721

Score:70.7 bits(77), Expect:4e-11,

Identities:93/128(73%), Gaps:1/128(0%), Strand: Plus/Plus

Query 4334 ACAG-TCAAGGGTAATATTTATCTTGCAAAAGTGGCTCGCGTAGAGCCCTCTCTTCAAGC 4392

|||| |||| || ||||| ||| | || ||||| || | ||||| || | | |||||

Sbjct 1159594 ACAGATCAAAGGAAATATATATTTAGCCAAAGTAACTAGAGTAGAACCTGCCTTGCAAGC 1159653

Query 4393 TGCTTTTGTTGATTATGGCGGCAATCGTCATGGTTTTTTGGCTTTTAGTGAAATTCATCC 4452

|||||||||||||||||||| | | || || | |||||| |||||| |||||

Sbjct 1159654 TGCTTTTGTTGATTATGGCGACGAAAAAAGCGGGTTCCTTCCTTTTAATGAAATCCATCC 1159713

Query 4453 GGACTATT 4460

|| ||||

Sbjct 1159714 TGATTATT 1159721

Range 13: 1274160 to 1274240

Score:70.7 bits(77), Expect:4e-11,

Identities:64/81(79%), Gaps:0/81(0%), Strand: Plus/Minus

Query 21202 CATTTTGCCGAGTTCCTTAGAGAAAGTTGTCTCGCGCCCCTAGGTATTCTCTACCTACCC 21261

|| |||||| |||||||| | ||| |||| || ||| ||||| |||||||| ||

Sbjct 1274240 CAATTTGCCTAGTTCCTTCAGCATCGTTCTCTCAAGCGCCTTGGTATACTCTACCTGTCC 1274181

Query 21262 ACCTGTGTCGGTTTCGGGTAC 21282

|||||||||||||| ||||||

Sbjct 1274180 ACCTGTGTCGGTTTAGGGTAC 1274160

Range 14: 1285254 to 1285505

Score:68.0 bits(74), Expect:1e-10,

Identities:173/262(66%), Gaps:20/262(7%), Strand: Plus/Minus

Query 7543 ACTTGCGATTTAATCTGGCGATATGCTGGCGATGATTCTACGGACTTTAATCATTATAGT 7602

||||| || |||| ||||| ||||| || ||| | || ||| |||||| |||||||

Sbjct 1285505 ACTTGTGACCTAATTTGGCGCTATGCCGGTGATAAGTCCGAGGATTTTAATTATTATAGC 1285446

Query 7603 AAAAGAAGTTTACTGACCGGTGTTTACCTCTCTTCAATAATGTTTTATATAAAAGATGAA 7662

||| || | | || || ||||| ||||| | ||||| || |||| |

Sbjct 1285445 AAACGAGCTCTTTTGCTTGGCGTTTATACATCTTCTCGGTTATTTTACTTATCAGATAAT 1285386

Query 7663 TCTAAAGATTACATTGAAACTGATAATTATATTGACCGAACG--CTTACAAAA-ATTATA 7719

|| ||||||| | |||| | | | ||||| ||| | || ||| ||||||

Sbjct 1285385 TCAAAAGATTTTACCAAAACCAAAGAATTTATTGC---AACTTCCCTAGAAAGGATTATA 1285329

Query 7720 AATATAGCT-------AGTTTaaaaaaaTTTGCTAAATTACCTTCTATTGAAGATATACC 7772

|||||||| || ||||| || || | |||||| |||||||||||||

Sbjct 1285328 AATATAGCCCAAACCAAGAGTAAAATAA-------AACTTCCTTCTGTTGAAGATATACC 1285276

Query 7773 AATACTAAGACTATTTTCTTaa 7794

|||| |||||||| | || |||

Sbjct 1285275 AATATTAAGACTAATGTCGTAA 1285254

Range 15: 1273970 to 1274060

Score:66.2 bits(72), Expect:5e-10,

Identities:69/91(76%), Gaps:0/91(0%), Strand: Plus/Minus

Query 21500 GGAATATTAACCCGTTGCCCATCGACTACGCCTCTCGGCCTGATCTTAGGACCTGACTCA 21559

|||||||||||| | | |||||||||||||||| || |||| |||||| | |||| |

Sbjct 1274060 GGAATATTAACCTGATTCCCATCGACTACGCCTTTCAGCCTCGCCTTAGGGGCCGACTAA 1274001

Query 21560 CCCTCCGTGGACGAACCTTGCGGAGGAACCC 21590

|||| || || | | || || ||||| ||

Sbjct 1274000 CCCTGCGCAGATTAGCTTTACGCAGGAAACC 1273970

Range 16: 913030 to 913107

Score:65.3 bits(71), Expect:2e-09,

Identities:61/78(78%), Gaps:0/78(0%), Strand: Plus/Minus

Query 3426 AATATTTGTCAATGCAGCTGAAATAATGCTGGGAATATCAATGCCAGAATGCGTATAAAG 3485

|| ||||| ||||| ||| ||||| ||| |||||||| || | ||| ||||||

Sbjct 913107 AACATTTGCAAATGCCGCTCTGATAATACTGAATATATCAATACCGGGATGACTATAAAA 913048

Query 3486 ATTTTTATCCTCAGCTGC 3503

||||||||||||||||||

Sbjct 913047 ATTTTTATCCTCAGCTGC 913030

Range 17: 1056809 to 1056912

Score:60.8 bits(66), Expect:2e-08,

Identities:77/105(73%), Gaps:1/105(0%), Strand: Plus/Plus

Query 877 TCAGGAGGTCAAAAACAGCGTGTGGCCATTGCACGCGCACTGATGATGGATCCGCCAGAT 936

|| || || ||||| || | || || || ||||| || |||||||| |||| | ||

Sbjct 1056809 TCCGGCGGCCAAAAGCAAAGGGTTGCAATAGCACGTGCCTTGATGATGAATCCTGAA-AT 1056867

Query 937 TATCCTATTTGACGAGCCTACCTCTGCACTTGACCCCGAAATGGT 981

||| |||||||| || ||||| ||||| | || || ||| ||||

Sbjct 1056868 TATGCTATTTGATGAACCTACTTCTGCGTTAGATCCTGAAGTGGT 1056912

Range 18: 139792 to 139885

Score:58.1 bits(63), Expect:3e-07,

Identities:72/95(76%), Gaps:3/95(3%), Strand: Plus/Minus

Query 15353 AAACCTTTTAGAAAATCATCAACAAAACAAAATAATGTGTCAAAATCTATATTCATTGGG 15412

|||| ||||| ||||||||| ||||| ||| | | || ||||||| | |||| | ||

Sbjct 139885 AAACTTTTTATAAAATCATCGACAAAGCAATAAATTGCTATAAAATCTTTTTTCA-TAGG 139827

Query 15413 GTATCTTTA-TTTGTAAAAAACATAGAAT-ACCTA 15445

||| |||| || || ||||||| | ||| |||||

Sbjct 139826 TTATTTTTAGTTGGTGAAAAACACAAAATAACCTA 139792

Range 19: 1245972 to 1246067

Score:58.1 bits(63), Expect:3e-07,

Identities:72/98(73%), Gaps:2/98(2%), Strand: Plus/Minus

Query 16421 TAGGTCACTTTGGTTCATatttagcagattttttatatcagttatttggccttgcctctt 16480

||||| | |||||||| ||| |||| ||| | || ||||| | ||||| |||| ||||

Sbjct 1246067 TAGGTTATTTTGGTTCTTATCTAGCTGATATATTTTATCAACTTTTTGGAGTTGCATCTT 1246008

Query 16481 atatttttccactttgttttttctcttttgcttttatc 16518

|||| ||||| |||| ||| | | || | |||||

Sbjct 1246007 TTATTATTCCA--TTGTCATTTATTGTCTGGTCTTATC 1245972

Range 20: 1159982 to 1160050

Score:53.6 bits(58), Expect:3e-06,

Identities:53/69(77%), Gaps:0/69(0%), Strand: Plus/Plus

Query 4715 TATAAGATTCAAGAGGTTATACATAAAAATCAAATTCTTTTTGTACAAGTCGTTAAAGAA 4774

||||| || || ||||||||| | ||| || |||||||| |||||||| ||||||

Sbjct 1159982 TATAAAATACAGGAGGTTATAAAAAAAGGACAGATTCTTTTGGTACAAGTTACAAAAGAA 1160041

Query 4775 GAACGCGGC 4783

|| || |||

Sbjct 1160042 GAGCGGGGC 1160050

1. [↑](#footnote-ref-1)
