# Supplementary material for: Rickettsial DNA and a trans-splicing rRNA group I intron in the unorthodox mitogenome of the fern Haplopteris ensiformis
Source: Commun Biol. 2023 Mar 20;6:296. doi: 10.1038/s42003-023-04659-8 (PMC10027690; doi:10.1038/s42003-023-04659-8)
Supplement: Supplementary file 11 — Reporting Summary [file 42003_2023_4659_MOESM11_ESM.pdf]

nature portfolio

Corresponding author(s): COMMSBIO-22-3386B

Last updated by author(s): Jan 5, 2023

Reporting Summary

Nature Portfolio wishes to improve the reproducibility of the work that we publish. This form provides structure for consistency and transparency in reporting. For further information on Nature Portfolio policies, see our Editorial Policies and the Editorial Policy Checklist.

Statistics

For all statistical analyses, confirm that the following items are present in the figure legend, table legend, main text, or Methods section.

n/a	Confirmed	
☒	☐	The exact sample size (*n*) for each experimental group/condition, given as a discrete number and unit of measurement
☒	☐	A statement on whether measurements were taken from distinct samples or whether the same sample was measured repeatedly
☒	☐	The statistical test(s) used AND whether they are one- or two-sided<br>*Only common tests should be described solely by name; describe more complex techniques in the Methods section.*
☒	☐	A description of all covariates tested
☒	☐	A description of any assumptions or corrections, such as tests of normality and adjustment for multiple comparisons
☒	☐	A full description of the statistical parameters including central tendency (e.g. means) or other basic estimates (e.g. regression coefficient) AND variation (e.g. standard deviation) or associated estimates of uncertainty (e.g. confidence intervals)
☒	☐	For null hypothesis testing, the test statistic (e.g. *F*, *t*, *r*) with confidence intervals, effect sizes, degrees of freedom and *P* value noted<br>*Give P values as exact values whenever suitable.*
☒	☐	For Bayesian analysis, information on the choice of priors and Markov chain Monte Carlo settings
☒	☐	For hierarchical and complex designs, identification of the appropriate level for tests and full reporting of outcomes
☒	☐	Estimates of effect sizes (e.g. Cohen's *d*, Pearson's *r*), indicating how they were calculated

Our web collection on statistics for biologists contains articles on many of the points above.

Software and code

Policy information about availability of computer code

Data collection	Data has been collected from and compared to the National Center for Biotechnology Information Nucleotide Archive (NCBI; https://www.ncbi.nlm.nih.gov/).
Data analysis	We used published open source software to analyze data, as indicated and cited in the manuscript:<br>FastQC v0.11.9 (http://www.bioinformatics.babraham.ac.uk/projects/fastqc/); MEGAHIT v1.2.9 software (10.1093/bioinformatics/btv033); NOVOPlasty 2.3.2 (10.1093/nargab/lqz011); Trinity v2.8.2 software (10.1038/nprot.2013.084); BLAST 2.9.0+ (10.1016/S0022-2836(05)80360-2)

For manuscripts utilizing custom algorithms or software that are central to the research but not yet described in published literature, software must be made available to editors and reviewers. We strongly encourage code deposition in a community repository (e.g. GitHub). See the Nature Portfolio guidelines for submitting code & software for further information.

Data

Policy information about availability of data

All manuscripts must include a data availability statement. This statement should provide the following information, where applicable:

- Accession codes, unique identifiers, or web links for publicly available datasets
- A description of any restrictions on data availability
- For clinical datasets or third party data, please ensure that the statement adheres to our policy

Haplopteris ensiformis primary nucleotide sequence reads are submitted to the sequence read archive (SRA) under BioProject accession number PRJNA862965. The assembled chloroplast genome is deposited under accession number OM867544 and the assembled mitogenome chromosomes are available under accession numbers OM867545 to OM867553.

Human research participants

Policy information about studies involving human research participants and Sex and Gender in Research.

Reporting on sex and gender	This study did not involve human research participants.
Population characteristics	*Describe the covariate-relevant population characteristics of the human research participants (e.g. age, genotypic information, past and current diagnosis and treatment categories). If you filled out the behavioural & social sciences study design questions and have nothing to add here, write "See above."*
Recruitment	*Describe how participants were recruited. Outline any potential self-selection bias or other biases that may be present and how these are likely to impact results.*
Ethics oversight	*Identify the organization(s) that approved the study protocol.*

Note that full information on the approval of the study protocol must also be provided in the manuscript.

Field-specific reporting

Please select the one below that is the best fit for your research. If you are not sure, read the appropriate sections before making your selection.

☐ Life sciences ☐ Behavioural & social sciences ☒ Ecological, evolutionary & environmental sciences

For a reference copy of the document with all sections, see nature.com/documents/nr-reporting-summary-flat.pdf

Ecological, evolutionary & environmental sciences study design

All studies must disclose on these points even when the disclosure is negative.

Study description	We present the organelle genomes of Haplopteris ensiformis (Pteridaceae, Polypodiales). Pteridaceae are a taxonomically and ecologically rich family of ferns. No complete organellar genomes have been sequenced yet. De novo genome and transcriptome assemblies were generated from nucleic acid preparations. The assembly results were compared to different nucleic acid preparations from the same species that grow in the botanical garden Bonn.
Research sample	Haplopteris ensiformis is a species of fern in the family Pteridaceae (Taxonomy ID: 38644). Haplopteris ensiformis is cultivated in the botanic garden Bonn. Taxonomical determination of the sample followed an inspection of morphology and sequencing of chloroplast genes atpB and rbcL for which reference sequences are available at the NCBI nucleotide archive. Genomic DNA and RNA were extracted from the complete, adult plant. In the botanical garden, Bonn the fern Vittaria lineata grows as a close relative of Haplopteris ensiformis. We investigated distinct loci of VIttaria lineate exemplarily for comparison with Haplopteris ensiformis.
Sampling strategy	Previous studies indicated that Pteridaceaen ferns feature highly interesting organellar genomes. In contrast to most fern organisms, vittariacean ferns, including Haplopteris, have comparable small nuclear genomes. From previous experimentation we achieved high quality DNA and RNA isolations for Haplopteris ensiformis.
Data collection	I, Simon Maria Zumkeller, collected data from public database NCBI nucleotide archive for sequence comparison. The assembly results were verified by PCRs that were performed by myself and co-author Monika Polsakiewicz.
Timing and spatial scale	First samples of Haplopteris ensiformis were processed 2018-01-11. Last samples were processed 2020-07-17
Data exclusions	No data was excluded.
Reproducibility	The de novo assemblies of Haplopteris ensiformis organellar DNAs were controlled via PCRs on other nucleic acid isolations from different time points. The sequencing results of multiple loci over two different samples of Haplopteris ensiformis were uniform.

Randomization	Our choice for Haplopteris ensiformis was driven by a profound investigation of literature, sample availability and sample handling during experimentation.
Blinding	*Describe the extent of blinding used during data acquisition and analysis. If blinding was not possible, describe why OR explain why blinding was not relevant to your study.*

Did the study involve field work? ☐ Yes ☒ No

Reporting for specific materials, systems and methods

We require information from authors about some types of materials, experimental systems and methods used in many studies. Here, indicate whether each material, system or method listed is relevant to your study. If you are not sure if a list item applies to your research, read the appropriate section before selecting a response.

Materials & experimental systems

n/a	Involved in the study	
☒	☐	Antibodies
☒	☐	Eukaryotic cell lines
☒	☐	Palaeontology and archaeology
☒	☐	Animals and other organisms
☒	☐	Clinical data
☒	☐	Dual use research of concern

Methods

n/a	Involved in the study	
☒	☐	ChIP-seq
☒	☐	Flow cytometry
☒	☐	MRI-based neuroimaging
